# Predictors of glaucoma knowledge and its risk factors among Jordanian patients with primary open angle glaucoma at a tertiary teaching hospital: A cross-sectional survey

**DOI:** 10.1371/journal.pone.0285405

**Published:** 2023-05-18

**Authors:** Sana’ Muhsen, Leen Al-Huneidy, Ward Maaita, Lina AlQirem, Zaid Madain, Jaleel Sweis, Raya Abu Tawileh, Yazan Al-Huneidy, Amro Alkhatib, Abdallah Al-Ani

**Affiliations:** 1 Special Surgery Department/Ophthalmology Division, School of Medicine, University of Jordan, Amman, Jordan; 2 School of Medicine, University of Jordan, Amman, Jordan; 3 Office of Scientific Affairs and Research, King Hussein Cancer Center, Amman, Jordan; Aravind Eye Hospital and Post Graduate Institute of Ophthalmology, INDIA

## Abstract

**Purpose of study:**

To assess and compare glaucoma knowledge between Jordanian patients with glaucoma and non-glaucoma ophthalmic patients.

**Methods:**

A cross-sectional survey was developed after an extensive literature search to investigate glaucoma-related knowledge among participants with glaucoma visiting the Jordan University Hospital clinics from October 2021 to February 2022. Responses were compared to a sample of ophthalmic participants with eye conditions other than glaucoma visiting the ophthalmology clinics at the same time frame.

**Results:**

A total of 256 participants filled out the survey, of which 53.1% were diagnosed with glaucoma while 46.9% had ophthalmic conditions other than glaucoma. Our sample of participants is characterized by a mean age of 52.2 ± 17.8 years and a male-to-female ratio of 1.04:1. Overall, participants with glaucoma were more aware of their disease than participants with other ophthalmic conditions. Compared to their ophthalmic non-glaucoma counterparts, those diagnosed with glaucoma face significantly more daily life difficulties due to their ophthalmic disease (p <0.001). Results of the independent sample *t*-test demonstrate that participants with glaucoma have significantly higher knowledge scores (p <0.001) and were able to recognize more glaucoma symptoms than their non-glaucoma counterparts (p = 0.002). Similarly, those with a positive family history of glaucoma displayed higher knowledge (p = 0.005). Multivariate linear regression demonstrates that family history of glaucoma, higher symptom recognition score, reliance on ophthalmologists, and the internet for glaucoma-related information are positive predictors of higher knowledge scores.

**Conclusion:**

We have demonstrated that both glaucoma and non-glaucoma ophthalmic patients display average levels of glaucoma knowledge. Raising awareness through various interventions may improve the lifestyles of patients with glaucoma and alleviate the economic burden associated with treating the disease.

## Introduction

Glaucoma encompasses a spectrum of diseases characterized by degeneration of the retinal ganglion cells leading to optic disc damage and irreversible vision loss [1]. Being the second most common cause of blindness worldwide, glaucoma affected 64.3 million adults in 2013 and is expected to affect 111.8 million by 2040 [2,3]. Due to its asymptomatic nature, lack of awareness, and low levels of referral, the prevalence of glaucoma is under-reported [4]. Additionally, the chronicity of the disease implies that patients with glaucoma may be subjected to various quality of life and therapeutic concerns due to long-term noncompliance, gender discrimination, and health literacy, all of which are influenced by the patient’s awareness towards their disease [4,5].

Despite the discrepancies between developed and developing nations in terms of knowledge of patients with glaucoma [6,7], the overall level of knowledge is poor [8,9]. Throughout the literature, factors such as socioeconomic status, short disease duration, and advanced age were negative predictors of glaucoma knowledge [8,10–12]. Poor glaucoma knowledge influences the clinical progression of the disease as it contributes to a lack of adherence to palliative drug regimes, and the underestimation of regular ophthalmic examinations [2,5,13]. Subsequently, those poor clinical presentations are associated with worse treatment outcomes as cases might be treated with more complex surgical options to halt the rapidly progressive and irreversible retinal damage [5,14]. Therefore, knowledge of glaucoma could be of vital importance as a target of interventions aiming to improve the lifestyles among those with glaucoma, particularly high-risk groups (e.g., geriatrics). Gatwood et al., (2022) demonstrate that disease-related educational interventions improve adherence to glaucoma treatment [15].

The literature evaluating glaucoma knowledge within the Middle East and therefore its treatment implications, is scarce. In fact, an accurate estimation of knowledge is tricky to calculate due to the juxtaposition with the concept of awareness. It should be noted that awareness refers to the generalized or diffuse knowledge of a particular concept (e.g., have you heard of glaucoma?), while knowledge refers to any information that is factual in nature (e.g., prevalence, etiology, risk factors, etc.) [16].

Among Jordanians, glaucoma is the third leading cause of severe bilateral blindness [17]. Glaucoma awareness throughout the population was estimated to be approximately 38.8% [18]. However, there exist no reports evaluating glaucoma knowledge among Jordanians. Thus, this study aims to assess the glaucoma knowledge and adherence to care seeking behaviors among Jordanian patients with glaucoma to formulate relevant educational policies and compliance assuring strategies.

## Materials and methods

We conducted this cross-sectional study using a self-administered questionnaire on patients visiting the ophthalmology clinics at Jordan University Hospital from October 2021 to February 2022. The Jordan University Hospital is the largest tertiary and academic health provider in Jordan. In addition, it is the largest referral center for all of central Jordan serving over four million patients.

Patients were enrolled into two groups: The first group consisted of participants with a clinically proven diagnosis of primary open angle glaucoma of at least 6 months and are on medications. Diagnosis of glaucoma was established using slit lamp examination, visual field testing, gonioscopy, and imaging (i.e., Optical Coherence Tomography). Patients with secondary glaucoma (e.g., pseudoexfoliation syndrome, pigment dispersion syndrome, neovascular glaucoma, inflammatory glaucoma, and medication-induced glaucoma) were excluded. The second group of participants consisted of those who were diagnosed with any vision limiting ophthalmic conditions with the exception of long-standing glaucoma. Such conditions include cataracts, diabetic retinopathy, macular degeneration, optic neuritis/atrophy, cerebrovascular disease, and hereditary retinal disorders. Participants that were younger than 18 years of age, suffered from mental/psychiatric illnesses, and were unable to consent to participation in the survey were excluded from the study. Moreover, participants that failed to complete at least 80% of the questionnaire were also excluded. Systemic random sampling was adopted to recruit every 4^th^ patient from the ophthalmology clinics.

### Instrument development

The study’s questionnaire was developed based on an extensive literature review of the most relevant articles reporting on the perceived knowledge of glaucoma by both patients with or without glaucoma [12,19–25]. The questionnaire is composed of the following sections: demographics (5 items), medical history (10 items), perceived glaucoma knowledge (31 items), and a miscellaneous section (4 items). The first 27 statements of the glaucoma knowledge section were presented as Likert-scales with 1 being strongly disagree and 5 being strongly agree. Statement 28 (signs and symptoms of glaucoma), statement 29 (treatment options for glaucoma), and statement 30 (complications of glaucoma treatment) were presented as non-mutually exclusive multiple choices. Miscellaneous items included statements exploring sources of glaucoma-related information, daily life difficulties due to ophthalmic conditions, ways on how to improve glaucoma awareness, and impact of surgical treatment on patients undergoing glaucoma surgery.

The questionnaire items were extracted from the literature, therefore, preserving their external validity. Furthermore, a panel of two expert ophthalmologists and four senior residents have approved the content of the questionnaire and slightly edited some of its statements to fit ophthalmic practice at the Jordan University Hospital. A pilot test on 15 patients with glaucoma and 15 patients with ophthalmic disorders other than glaucoma yielded a Cronbach alpha of 0.704 for the 27 knowledge Likert-scales.

The questionnaire underwent back-to-back translation as it was translated from English to Arabic by an expert ophthalmologist and then back to English by an official translator in order to ensure the validity of the initial translation.

### Statistical analysis

All statistical analyses and data cleaning were conducted on SPSS version 24 (SPSS Inc., Chicago, IL, USA). Categorical data were reported as frequencies (n (%)), while continuous data were presented as means ± standard deviations. Mean differences among different categories were examined by the independent sample *t*-test or ANOVA. Statistical associations between categorical variables were assessed using the chi-square test. Upon conducting chi-square testing, the 5-point Likert-scales were condensed into three categories (strongly disagree and disagree as one category; neutral as one category; strongly agree and agree as one category). Knowledge scores were computed by adding how many correct answers that participants had out of the first 27 knowledge items (1 point per each correct statement resulting in a total score of 27). Symptoms and adverse effects recognition scores were computed as the sum of participants’ chosen categories. Pearson’s correlation testing was conducted to assess the correlation between continuous variables and knowledge scores. Moreover, a multivariate linear regression model was computed to assess predictors of knowledge score. All statistical tests were conducted with a 95% confidence interval and a 5% error margin. A p-value of less than 0.05 was considered statistically significant.

### Ethical considerations

The study’s protocol was approved by the Jordan University Hospital Institutional Review Board (ID:212/2022/09) and was issued on the 1^st^ of September 2021 by the aforementioned body. All participants had read and signed an informed written consent form before continuing to complete the questionnaire. The consent form included the participants’ right to anonymity, confidentially of their data, right to leave the study, and reassurance that their participation was completely voluntary, was not associated with any kind of short-term benefit or rewards, and did not affect the quality of their received care.

## Results

A total of 256 participants filled out the survey, of which 53.1% were diagnosed with glaucoma while 46.9% had ophthalmic conditions other than glaucoma. Our sample of participants was characterized by a mean age of 52.2 ± 17.8 years and a male to female ratio of 1.04:1. The majority of the sample resided in the capital city of Amman (66.8%), had a bachelor’s degree or higher (50.8%), and earned less than 500 JDs in monthly income (64.5%). Characteristics of participants with glaucoma were similar to those without glaucoma with the exception of age (p = 0.009), clinic visits per month (p <0.001), clinic visits per year (p <0.001), and family history of glaucoma (p = 0.006). **Table 1** describes the sociodemographic characteristics of the recruited cohort.

**Table 1:** Sociodemographic characteristics of the studied population.

	Ophthalmic non-glaucoma patients (n = 120)	Glaucoma patients (n = 136)	p-value*
Age in years [Mean ± SD (Median)]	48.1 ± 16.4 (49.5)	54.6 ± 18.2 (57.0)	**0.009**
Clinic visits per month [Mean ± SD (Median)]	0.5 ± 0.8 (0.0)	0.9 ± 0.7 (1.0)	**<0.001**
Clinic visits per year [Mean ± SD (Median)]	3.6 ± 5.6 (2.0)	7.0 ± 6.8 (4.0)	**<0.001**
**Biological Sex**			0.133
Male	55 (45.8)	76 (55.9)	
Female	65 (54.2)	60 (44.1)	
**Residence**			0.832
Amman (Capital)	80 (66.7)	91 (66.9)	
Central	29 (24.2)	30 (22.1)	
South	5 (4.2)	9 (6.6)	
North	6 (5.0)	6 (4.4)	
**Education**			0.415
Illiterate	8 (6.7)	12 (8.8)	
Primary	10 (8.3)	21 (15.4)	
Highschool	37 (30.8)	38 (27.9)	
Bachelors	52 (43.3)	54 (39.7)	
Master’s +	13 (10.8)	11 (8.1)	
**Income**			0.091
< 499	69 (57.5)	96 (70.6)	
500–999	39 (32.5)	30 (22.1)	
1000–1999	8 (6.7)	9 (6.6)	
> 2000	4 (3.3)	1 (0.7)	
**Comorbidities**			0.133
No	63 (52.5)	58 (42.6)	
Yes	57 (47.5)	78 (57.4)	
**Smoking history**			0.373
No	89 (74.2)	108 (79.4)	
Yes	31 (25.8)	28 (20.6)	
**Family history of glaucoma**			**0.006**
No	90 (77.6)	83 (61.5)	
Yes	26 (21.7)	52 (38.5)	

*Student’s t-test was used to test for differences among continuous variables (e.g., age); Chi-square test was used to text for associations between categorical variables (e.g., income); a p-value of less than 0.05% was considered statistically significant.

Amongst participants with glaucoma, the mean time since glaucoma diagnosis was 9.7 ± 9.4 years, and the mean time since the start of glaucoma treating drops was 9.1 ± 8.7 years. Participants with glaucoma used a mean of 2.4 ± 1.4 medications and a mean of 3.3 ± 2.9 drops per day for the treatment and/or control of their glaucoma. Furthermore, participants were subjected to a mean of 1.6 ± 2.7 surgeries for the treatment of glaucoma in their lifetime.

**Table 2** demonstrates the responses of participants towards glaucoma knowledge items. Overall, participants with glaucoma were more aware of their disease than participants with other ophthalmic conditions. Participants with glaucoma were significantly more likely than their non-glaucoma counterparts to recognize that glaucoma is a predominantly ophthalmic disease (*X*^2^(2) = 11.16, p = 0.004), is the result of increased intraocular pressure (*X*^2^(2) = 19.56, p <0.001), can progress to optic nerve damage (*X*^2^(2) = 29.41, p <0.001), and is more likely to increase in incidence with age (*X*^2^(2) = 9.13, p = 0.010). Moreover, they were more likely to think that stress (*X*^2^(2) = 6.05, p = 0.048), prolonged computer usage (*X*^2^(2) = 10.05, p = 0.007), prolonged smartphone usage (*X*^2^(2) = 11.59, p = 0.003), and prolonged reading (*X*^2^(2) = 9.15, p = 0.010) may worsen symptoms of glaucoma. Compared to ophthalmic non-glaucoma participants, those diagnosed with glaucoma were familiar with the nature of their treatment as they are vigilant of the ability of medications to control glaucoma (*X*^2^(2) = 19.98, p <0.001), and that glaucoma treatment is lifelong (*X*^2^(2) = 32.97, p <0.001).

**Table 2:** Responses of participants to knowledge items.

	Ophthalmic non-glaucoma patients			Glaucoma patients			
Items	Disagree	Neutral	Agree	Disagree	Neutral	Agree	p-value
Glaucoma is a disease that affects the eyes and no other part of the body.	4 (3.3)	34 (28.3)	82 (68.3)	5 (3.7)	16 (11.8)	115 (84.6)	**0.004**
Glaucoma is caused by raised intraocular pressure.	7 (5.8)	54 (45.0)	59 (49.2)	7 (5.1)	27 (19.9)	102 (75.0)	**0.000**
Glaucoma can lead to optic nerve damage and visual disorders.	1 (0.8)	47 (39.2)	72 (60.0)	1 (0.7)	14 (10.3)	121 (89.0)	**0.000**
Glaucoma has more than one type (e.g., open angle, closed angle, secondary, developmental)	3 (2.5)	92 (76.7)	25 (20.8)	3 (2.2)	93 (68.4)	40 (29.4)	0.290
Glaucoma can be inherited in the family.	19 (15.8)	55 (45.8)	46 (38.3)	25 (18.4)	47 (34.6)	64 (47.1)	0.182
Glaucoma is more common as you get older.	3 (2.5)	35 (29.2)	82 (68.3)	6 (4.4)	19 (14.0)	111 (81.6)	**0.010**
High blood pressure and diabetes increase the risk of glaucoma.	2 (1.7)	42 (35.0)	76 (63.3)	11 (8.1)	39 (28.7)	86 (63.2)	0.050
People with high myopia or hyperopia are more likely to get glaucoma.	20 (16.7)	58 (48.3)	42 (35.0)	16 (11.8)	72 (52.9)	48 (35.3)	0.507
The use of steroid /eye drops can cause glaucoma.	9 (7.5)	76 (63.3)	35 (29.2)	13 (9.6)	70 (51.5)	53 (39.0)	0.160
Medicines other than eye drops can influence intraocular pressure.	6 (5.0)	51 (42.5)	63 (52.5)	15 (11.0)	50 (36.8)	71 (52.2)	0.187
Stress can make glaucoma worse.	6 (5.0)	41 (24.2)	73 (60.8)	7 (5.1)	28 (20.6)	101 (74.3)	**0.048**
Using a computer will make glaucoma worse.	4 (3.3)	40 (33.3)	76 (63.3)	13 (9.6)	25 (18.4)	98 (72.1)	**0.007**
Using a smartphone will make glaucoma worse.	4 (3.3)	38 (31.7)	78 (65.0)	14 (10.3)	22 (16.2)	100 (73.5)	**0.003**
Fluorescent lights will make glaucoma worse.	10 (8.3)	59 (49.2)	51 (42.5)	20 (14.7)	62 (45.6)	53 (39.7)	0.286
A lot of reading may make glaucoma worse	11 (9.2)	47 (39.2)	62 (51.7)	13 (9.6)	30 (22.1)	93 (68.4)	**0.010**
High intraocular pressure must be addressed.	0 (0.0)	13 (10.8)	107 (89.2)	0 (0.0)	4 (2.9)	132 (97.1)	**0.021**
Glaucoma can be controlled by treatment.	4 (3.3)	34 (28.3)	82 (68.3)	8 (5.9)	10 (7.4)	118 (86.8)	**0.000**
Early detection and treatment will not slow the course of glaucoma.	70 (58.3)	28 (23.3)	22 (18.3)	82 (60.3)	15 (11.0)	39 (28.7)	**0.013**
It is possible to completely lose vision as a result of laser treatment or surgery for glaucoma.	24 (20.0)	58 (48.3)	38 (31.7)	34 (25.0)	58 (42.6)	44 (32.4)	0.558
The use of eye drops will be redundant if one has had a laser treatment or surgery for glaucoma.	32 (26.7)	60 (50.0)	28 (23.3)	34 (25.0)	60 (44.1)	32 (30.9)	0.393
Follow-up visit to the clinic is not necessary after the laser treatment or surgery for glaucoma.	81 (67.5)	19 (15.8)	20 (16.7)	91 (66.9)	13 (9.6)	32 (23.5)	0.175
Regular check-ups are not necessary for glaucoma patients.	89 (74.2)	17 (14.2)	14 (11.7)	106 (77.9)	7 (5.1)	23 (16.9)	**0.032**
Even if the intraocular pressure is under control, the visual field has to be checked.	1 (0.8)	34 (28.3)	85 (70.8)	2 (1.5)	18 (13.2)	116 (85.3)	**0.011**
A patient should always tell the ophthalmologist which other medicines (s)he is taking.	2 (1.7)	8 (6.7)	110 (91.7)	0 (0.0)	4 (2.9)	132 (97.1)	0.114
A patient should always tell the ophthalmologist which other diseases (s)he has.	2 (1.7)	9 (7.5)	109 (90.8)	0 (0.0)	3 (2.2)	133 (97.8)	**0.041**
Treatment for glaucoma is lifelong.	23 (19.2)	69 (57.5)	28 (23.3)	13 (9.6)	43 (31.6)	80 (58.8)	**0.000**
Vision loss is reversible after initiating glaucoma treatment.	21 (17.5)	57 (47.5)	42 (35.0)	60 (44.1)	42 (30.9)	34 (25.0)	**0.000**

Nonetheless, both groups of participants portrayed variability in their awareness towards types of glaucoma, certain risk factors associated with glaucoma (i.e., myopia), effect of certain medications on glaucoma development (e.g., corticosteroid containing drops), and the post-operative implications of the surgical management of glaucoma.

Most participants were aware of glaucoma symptoms with the exception of convulsive seizures (9.0%). Moreover, only 27.3% were aware that glaucoma can manifest as an asymptomatic entity. Participants with glaucoma were significantly more likely to report vision loss (p = 0.008), and pain, redness, nausea, and vomiting (*X*^2^(1) = 12.11, p = 0.001) as symptoms of glaucoma than their ophthalmic non-glaucoma counterparts. Similarly, the treatment of glaucoma with medications is significantly more recognized by participants with glaucoma (*X*^2^(1) = 8.11, p = 0.006). The least reported modality of treatment among both groups was surgery (57.5%, and 69.1%).

The least commonly recognized complications of glaucoma treatment were dyspnea (10.9%), bradycardia (12.1%), and elongation of eyelashes (18.0%). In terms of sources of information, participants with glaucoma were significantly more dependent on ophthalmologists for disease-specific information (*X*^2^(1) = 80.62, p < 0.001). On the other hand, ophthalmic non-glaucoma participants were significantly more likely to rely on close social circles (*X*^2^(2) = 15.28, p < 0.001). **Table 3** describes knowledge about glaucoma presentation and its treatment.

**Table 3:** Awareness of risk factors, treatment options, and difficulties associated with glaucoma.

	Ophthalmic non-glaucoma patients	Glaucoma patients	p-value*
**Signs & symptoms of glaucoma**			
Asymptomatic	29 (24.2)	41 (30.1)	0.326
Reduced visual acuity	106 (88.3)	123 (90.4)	0.685
Narrow field of vision	62 (51.7)	85 (62.5)	0.100
Vision loss	55 (45.8)	85 (62.5)	**0.008**
Convulsive seizures	11 (9.2)	12 (8.8)	1.000
Pain, redness, nausea, and vomiting	41 (34.2)	76 (55.9)	**0.001**
**Treatment options for glaucoma**			
Medications	75 (62.5)	107 (78.7)	**0.006**
Surgical procedures	93 (77.5)	118 (86.8)	0.070
Laser therapy	69 (57.5)	94 (69.1)	0.068
**Complications of treatment**			
Stinging and burning	90 (75.0)	96 (70.6)	0.483
Blurred vision	87 (72.5)	96 (70.6)	0.782
Discoloration of iris	29 (24.2)	29 (21.3)	0.654
Dyspnea	10 (8.3)	18 (13.2)	0.234
Tachypnea	15 (12.5)	16 (11.8)	1.000
Elongation of lashes	9 (7.5)	37 (27.2)	**0.000**
Redness, dryness, and itching	64 (53.3)	100 (73.5)	**0.001**
**Source of information**			
Ophthalmologists	35 (29.2)	115 (84.6)	**0.000**
Non-ophthalmologists	10 (8.3)	8 (5.9)	0.472
Friends and family	53 (44.2)	29 (21.3)	**0.000**
TV/Newspapers	32 (26.7)	27 (19.9)	0.234
Internet	45 (37.5)	56 (41.2)	0.609
Social Media	21 (17.5)	23 (16.9)	1.000
**Difficulties due to ophthalmic conditions**			
Identifying persons	27 (22.5)	54 (39.7)	**0.005**
Reading	39 (32.5)	73 (53.7)	**0.001**
Using stairs	21 (17.5)	44 (32.4)	**0.009**
Watching TV	31 (25.8)	54 (39.7)	**0.024**
Driving	30 (25.0)	52 (38.2)	**0.031**
Use social media	23 (19.2)	25 (25.7)	0.233
Walking alone	14 (11.7)	32 (23.5)	**0.015**
Differentiating colors and clothes	20 (16.7)	28 (20.6)	0.521
No difficulties	70 (58.3)	43 (31.6)	**0.000**

*Chi-square test was used to text for associations between categorical variables (e.g., income); a p-value of less than 0.05% was considered statistically significant.

Compared to their ophthalmic non-glaucoma counterparts, those diagnosed with glaucoma faced significantly more daily life difficulties due to their ophthalmic disease (p <0.001). Such difficulties included identifying persons (p = 0.005), reading (p = 0.001), using the stairs (p = 0.009), driving (p = 0.031), walking alone (p = 0.015), among others. Overall, most participants believed that more information on the pathophysiology/progression (83.6%), treatment (68.4%) of glaucoma, and other practical advice on how to manage disease fluctuations (71.1%) are needed to improve awareness and knowledge of glaucoma.

Univariate analysis shows that participants with glaucoma have significantly higher knowledge scores (t(254) = 4.31, p <0.001) and were able to recognize more glaucoma symptoms than their non-glaucoma counterparts (t(254) = 3.18, p = 0.002). Similarly, those with a positive family history of glaucoma displayed higher knowledge (t(249) = 2.81, p = 0.005) and a relatively higher ability to recognize glaucoma symptoms (t(249) = 2.07, p = 0.039). Knowledge scores did not differ between genders, area of residence, educational status, income level, or medical history (refer to **Table 4**). Moreover, the ability to recognize more symptoms and more treatment-related complications was positively correlated with higher knowledge scores (r = 0.302, p <0.001; r = 0.285, p <0.001, respectively). However, age (p = 0.130), number of clinic visits per month (p = 0.410), and number of clinic visits per year (p = 0.412) were not significantly correlated with knowledge scores.

**Table 4:** Factors affecting glaucoma knowledge scores.

	Knowledge Score	p-value	Symptom recognition	p-value	Adverse effect recognition	p-value
**Disease Status**		**0.000**		**0.002**		0.074
Ophthalmic non-glaucoma patients	14.6 ± 6.1		2.5 ± 1.4		2.5 ± 1.6	
Glaucoma patients	17.5 ± 4.1		3.1 ± 1.4		2.9 ± 1.5	
**Biological Sex**		0.442		**0.028**		0.204
Male	16.4 ± 5.3		3.0 ± 1.5		2.8 ± 1.7	
Female	15.8 ± 5.4		2.6 ± 1.4		2.6 ± 1.4	
**Residence**		0.856		0.929		0.642
Amman (Capital)	16.1 ± 5.3		2.8 ± 1.4		2.7 ± 1.6	
Central	15.8 ± 6.1		2.8 ± 1.4		2.7 ± 1.4	
South	17.1 ± 2.8		2.6 ± 1.3		2.3 ± 1.1	
North	16.6 ± 4.1		2.6 ± 1.7		2.3 ± 1.1	
**Education**		0.076		0.440		0.114
Illiterate	16.3 ± 6.0		3.1 ± 1.4		3.3 ± 1.6	
Primary	16.2 ± 4.6		3.1 ± 1.3		2.6 ± 1.3	
Highschool	14.7 ± 6.2		2.7 ± 1.6		2.6 ± 1.6	
Bachelors	16.8 ± 4.6		2.7 ± 1.4		2.5 ± 1.5	
Master’s +	17.4 ± 5.1		3.0 ± 1.4		3.2 ± 1.7	
**Income**		0.077		0.460		0.110
< 499	16.0 ± 5.4		2.9 ± 1.5		2.7 ± 1.5	
500–999	16.4 ± 5.1		2.6 ± 1.4		2.5 ± 1.5	
1000–1999	15.1 ± 5.3		2.9 ± 1.1		2.4 ± 1.2	
> 2000	22.0 ± 1.0		3.4 ± 0.8		4.2 ± 1.8	
**Comorbidities**		0.346		0.448		0.938
No	15.8 ± 5.9		2.9 ± 1.4		2.7 ± 1.6	
Yes	16.4 ± 4.7		2.7 ± 1.4		2.7 ± 1.5	
**Smoking history**		0.699		0.754		0.471
No	16.1 ± 5.3		2.8 ± 1.5		2.7 ± 1.5	
Yes	16.4 ± 5.6		2.9 ± 1.4		2.8 ± 1.5	
**Family history of glaucoma**		**0.005**		**0.039**		0.154
No	15.5 ± 5.8		2.7 ± 1.4		2.6 ± 1.5	
Yes	17.5 ± 3.9		3.1 ± 1.5		2.9 ± 1.6	

Multivariate linear regression demonstrates that a family history of glaucoma (B: 1.539; 95%CI: 0.078–3.000; p-value: 0.039), higher symptom recognition score (B: 0.624; 95%CI: 0.083–1.164, p-value: 0.024), relying on ophthalmologists (B: 2.584; 95%CI: 0.983–4.185; p-value: 0.002), and the internet (B: 2.364; 95%CI: 0.845–3.883: p-value: 0.002) for glaucoma-related information are positive predictors of higher knowledge scores. **Table 5** delineates the linear regression model for knowledge scores.

**Table 5:** Predictors of knowledge score for the studied sample (n = 256).

	Linear regression for knowledge score				
	p-value	B	ß	Lower 95% CI for (B)	Upper 95% CI for (B)
Age	0.390	0.019	0.067	-0.025	0.064
Female gender	0.334	0.672	0.065	-0.696	2.041
Male gender (**Reference**)	—	—	—	—	—
Family history of Glaucoma	**0.039**	1.539	0.138	0.078	3.000
No family history of Glaucoma (**Reference**)	—	—	—	—	—
Positive presence of comorbidities	0.712	0.290	0.028	-1.259	1.840
Smoking	0.425	0.651	0.052	-0.957	2.260
No smoking (**Reference**)	—	—	—	—	—
Clinic Visit Per Year	0.724	-0.019	-0.025	-0.122	0.085
Symptom recognition score	**0.024**	0.624	0.175	0.083	1.164
Adverse effects recognition score	0.568	0.163	0.048	-0.399	0.724
Number of faced difficulties due to ophthalmic disease	0.445	0.108	0.055	-0.170	0.386
**Sources of information**					
Ophthalmologists	**0.002**	2.584	0.239	0.983	4.185
Non-Ophthalmologists	0.376	1.324	0.060	-1.621	4.268
Friends and Family	0.832	-0.174	-0.015	-1.783	1.436
TV/Newspapers	0.696	-0.344	-0.027	-2.081	1.392
Internet	**0.002**	2.364	0.227	0.845	3.883
Social Media	0.518	-0.631	-0.047	-2.554	1.292

## Discussion

Our study demonstrated that glaucoma knowledge among Jordanian ophthalmic patients is defective and extremely variable. Knowledge deficiencies are most pronounced in areas such as risk factors, neurological symptoms, adverse effects of over-the-counter medications of glaucoma, and post-operative management and expectations. Additionally, it appears that the extent of knowledge of recruited participants is only influenced by their glaucoma diagnosis and family history of glaucoma. Conversely, all other sociodemographic factors did not significantly impact overall knowledge.

The level of knowledge of glaucoma portrays significant variability across different reports throughout the globe. Jordanian reports show that only 34.1% of Jordanian patients were able to accurately define glaucoma despite their acceptable levels of awareness [26]. In terms of regional literature, a Saudi Arabian report demonstrates that patients with glaucoma exhibit poor knowledge, attitudes, and practices toward their disease [27]; a finding that was similarly observed among Iraqi ophthalmic patients [28]. An Iranian population-based survey demonstrated that only 46.6% demonstrated glaucoma awareness while only 19.2% were able to correctly define the disease [29]. Asian countries, the likes of Nepal and India, demonstrated low rates of glaucoma knowledge among hospital presenting patients ranging from 1.9% to 27% [30–32]. China, on the other hand, demonstrated that 51.6% of glaucoma patients visiting a tertiary university hospital were aware of their disease [33].

Great variance is noted among patients with glaucoma from African nation with rates ranging from 74% for Ghana, 36.8% for Nigeria, to 46.7% for Ethiopia [34–37]. On the other hand, Western nations such as Austria, Germany, the United States, Australia, and the United Kingdom display far superior rates of glaucoma knowledge ranging from 51% to 93% among both the public and patients visiting eye clinics [19,38–40].

While such variance among different studies could be attributed to differences between studied populations in terms of sociodemographic or clinical characteristics, inherent methodological differences make direct comparisons between reports extremely challenging. There seems to be an overlap, or rather a failure of distinction, between knowledge and awareness [22]. Across the aforementioned studies, measures of knowledge assessment were dramatically different in terms of content, format (e.g., number and type of questions), and definitions of proper knowledge, most of which were made based on arbitrary and/or convenient basis [41]. Furthermore, questionnaire-based measures were not always developed rigorously. Such discrepancies in development (e.g., presence or absence of pilot testing or expert group consultation) could have led to the observed heterogeneity of glaucoma-related knowledge.

We demonstrated that glaucoma patients are relatively more knowledgeable about glaucoma in comparison to ophthalmic non-glaucoma counterparts, yet the knowledge among the two groups was relatively poor. Such observation is fairly established within the literature and is expected as those with established glaucoma diagnosis may have received a greater amount of disease-specific knowledge from their doctors or close social circles at far higher frequencies of contact [23]. Literature shows that durations of glaucoma and follow-up are significantly associated with improved levels of knowledge [12,42]. Another plausible explanation is the reliance of ophthalmic non-glaucoma patients on friends and family for glaucoma-related information may predispose them to inaccurate or outdated information; a finding documented within a German survey [40]. Such reliance may indicate a gap of trust between patients and healthcare providers. On the other hand, the poor level of knowledge among patients with glaucoma could be attributed to the congestion of the Jordanian primary healthcare system which renders doctor-patient encounters less effective [26]. Increasing the number of physicians and fortifying their communication skills may ensure the appropriate delivery of vital glaucoma-related information to patients; however, this solution might be over-ambitious in resource-scarce institutions and developing nations.

Amongst the literature, several factors were implicated to have influenced glaucoma-related knowledge such as age, education, occupation, income, accessibility of ophthalmic care, duration of glaucoma, compliance to medications, family history of glaucoma, and type of glaucoma [20,22,33]. Chen et al., (2022) demonstrated a comprehensive review of such factors and showed that lack of conformity between the impact of different factors on glaucoma knowledge can be explained by differences in study design, characteristics of study populations, and methods of analysis [33]. In agreement with our investigation, several studies reported that participants with a family history of glaucoma were significantly more knowledgeable about their condition [12,26,40]. Such might be the case because having a family member with glaucoma may provoke the individual to search for more information and/or gather information during their appointment with the eye care professional [30]. Contrary to the literary consensus [21,25,33], educational level was not significantly associated with glaucoma knowledge among both groups of studied participants. This can be attributed to weakness in the Jordanian health-related educational programs for secondary and higher levels of education [26]. Additionally, while age did not significantly predict knowledge scores among included participants, it was a negative predictor of glaucoma knowledge within the literature. The latter could be attributed the low levels of health literacy in older individuals, neurological disease progression, or the inherent technological bias exhibited by younger participants [33,43].

Improving awareness was shown to help in early glaucoma detection, thus better control and recognition of its complications [25], which in turn will assist in mitigating healthcare costs and the overall clinical burden of disease [24]. We propose a number of evidence-based recommendations that may improve patients’ awareness to glaucoma. First, the implementation of a patient-held record (e.g., ‘Glaucoma Logbook) which encompasses everything related to glaucoma from its most common facts to the most relevant management details [44]. Second, the creation of a glaucoma-centric social club which was shown to predispose patients to higher levels of glaucoma knowledge, improved disease cognition, and improved compliance [45]. This can be administered through social media as services such as ‘WeChat’ was effective in improving patients’ education towards glaucoma and enhancing their willingness to utilize electronic services for their follow-up [23]. Third, the implementation of a telemedicine program which can both follow-up on patients with glaucoma and educate them at an extremely low direct and indirect cost [46]. Finally, the formulation of a patient-centered educational program was proven effective in enhancing the knowledge of patients with congenital and secondary glaucoma [47]. Such a program may take different forms ranging from seminars, written publications (e.g., brochures and leaflets), and video materials, to theory-based practical sessions given throughout a patient’s clinical visit. Among our participants, less than 50% of the proposed adverse effects of glaucoma medications were identified. Considering the wide spectrum of systemic side effects associated with beta-blockers and alpha-agonists, the aforementioned targeted solutions should focus on such deficiency [12].

The burden of ameliorating glaucoma awareness and knowledge should also be shared with governing bodies and health policymakers. Our multivariate analysis demonstrated that ophthalmologists and the internet are associated with increased levels of knowledge; an observation that was documented in China [33]. Concerned governmental authorities and health policymakers should increase the public’s awareness and knowledge by allocating more resources towards enhancing ophthalmology practice and utilizing internet-based solutions to reach a wide audience.

This study is not devoid of limitations. The small sample size might have impacted the statistical robustness by which knowledge scores are differentiated. Moreover, the study examines glaucoma knowledge in a different, yet more comprehensive, manner than the available literature thus it might underestimate its rates of glaucoma awareness. Moreover, data may be subjected to recall bias due to the self-reporting nature of the questionnaire. Finally, due to the lack of a standardized scoring system for glaucoma-related knowledge and awareness, direct comparisons with other studies proved difficult. Nonetheless, the strength of the study lies in its usage of a rigorously designed and validated questionnaire to evaluate glaucoma-related knowledge. Also, the study’s sample of glaucoma patients closely resembles the overall number of glaucoma patients treated at the JUH ophthalmology clinics thus high internal validity could be expected.

## Conclusion

In light of this investigation, we have demonstrated that ophthalmic patients display average levels of glaucoma knowledge. Raising awareness through various programs and interventions may improve the lifestyles of patients with glaucoma, which may result in long-term mitigation of the economic burden associated with the disease and its complications. Concerned authorities should project their interventions through ophthalmologists and internet-based platforms due to their profound effect in shaping glaucoma-related knowledge.

## Supporting information

S1 Data Data from the administered survey. (XLSX)

S1 File Data collection instrument (questionnaire). (PDF)
