# Supplementary material for: Predictors of glaucoma knowledge and its risk factors among Jordanian patients with primary open angle glaucoma at a tertiary teaching hospital: A cross-sectional survey
Source: PLoS One. 2023 May 18;18(5):e0285405. doi: 10.1371/journal.pone.0285405 (PMC10194903; doi:10.1371/journal.pone.0285405)
Supplement: S1 File — (PDF) [file pone.0285405.s002.pdf]

Questionnaire

Demographics

1. Age
2. Area of residence
3. Degree of education
4. Biological sex
5. Income

Medical history

1. Past medical history
2. Duration since glaucoma diagnosis
3. Duration since start of drops for glaucoma treatment
4. Times per day using drops for glaucoma
5. Number of clinic visits per month
6. Number of clinic visits per year
7. Number of glaucoma medications
8. Number of glaucoma related surgeries/laser
9. Family or close-friends with glaucoma diagnosis
10. Smoking history

Knowledge of glaucoma

1. Glaucoma is a disease that affects the eyes and no other part of the body.
2. Glaucoma is caused by raised intraocular pressure.
3. Glaucoma can lead to optic nerve damage and visual disorders.
4. Glaucoma has more than one type (eg: open angle, closed angle, secondary, developmental)
5. Glaucoma can be inherited in the family.
6. Glaucoma is more common as you get older.
7. High blood pressure and diabetes increase the risk of glaucoma.
8. People with high myopia or hyperopia are more likely to get glaucoma.
9. The use of steroid /eye drops can cause glaucoma.
10. Medicines other than eye drops can influence intraocular pressure.
11. Stress can make glaucoma worse.
12. Using a computer will make glaucoma worse.
13. Using a smartphone will make glaucoma worse.
14. Fluorescent lights will make glaucoma worse.
15. A lot of reading may make glaucoma worse
16. High intraocular pressure must be addressed.
17. Glaucoma can be controlled by treatment.
18. Early detection and treatment will not slow the course of glaucoma.
19. It is possible to completely lose vision as a result of laser treatment or surgery for glaucoma.
20. The use of eye drops will be redundant if one has had a laser treatment or surgery for glaucoma.
21. Follow-up visit to the clinic is not necessary after the laser treatment or surgery for glaucoma.
22. Regular check-ups are not necessary for glaucoma patients.
23. Even if the intraocular pressure is under control, the visual field has to be checked.
24. A patient should always tell the ophthalmologist which other medicines (s)he is taking.
25. A patient should always tell the ophthalmologist which other diseases (s)he has.
26. Treatment for glaucoma is lifelong.
27. Vision loss is reversible after initiating glaucoma treatment.

28. Choose the symptoms associated with glaucoma
    1. Normally it's asymptomatic.
    2. Reduced visual acuity
    3. Narrowed visual field
    4. Vision loss
    5. Convulsive seizure
    6. Pain, redness, nausea and vomiting during an acute attack
29. Treatment options of glaucoma include
    1. Medications
    2. Laser
    3. Surgery
30. Side effects of using eye drops for the treatment of glaucoma
    1. Stinging and burning of eyes
    2. Blurred vision after dropping
    3. Discoloration of the iris
    4. Dyspnea
    5. Slower heart rate
    6. Longer eyelashes
    7. Eyelid inflammation
31. Source of information
    1. Ophthalmologists
    2. Friends and Family
    3. Media (TV, Newspapers)
    4. Non-ophthalmic doctors
    5. Internet
    6. Social media
32. Everyday visual difficulties faced by patients with glaucoma
    1. No visual problems
    2. Difficulties with:
        i. Identifying persons outdoors
        ii. Reading
        iii. Walking on stairs
        iv. Watching TV
        v. Driving a car
        vi. Walking out alone
        vii. Reading street signs
        viii. Colours, choosing clothes
33. Aspects you feel should be improved regarding glaucoma knowledge and awareness:
    1. Better information about the disease
    2. Better information on treatment
    3. Better social contact
    4. Better follow up on negative feelings
    5. Better practical advices
    6. Nothing
34. Your state after undergoing laser or incisional surgery? (if applicable)
    1. Unchanged
    2. Better
    3. Worse

العمر

الجنس؟
- ☐ ذكر
- ☐ انثى

في اي محافظة تقيم؟
- ☐ اقليم العاصمة (عمان)
- ☐ الوسط (الزرقاء، البلقاء، مادبا)
- ☐ الجنوب (الكرك، معان، الطفيلة)
- ☐ الشمال (جرش، عجلون، اربد)

كم يتراوح دخلك الشهري؟
- ☐ اقل من ٥٠٠ دينار
- ☐ ٥٠٠ الى ٩٩٩ دينار
- ☐ ١٠٠٠ الى ٢٠٠٠ دينار
- ☐ ٢٠٠٠ الى ٥٠٠٠ دينار
- ☐ اكثر من ٥٠٠٠ دينار

ما هو مؤهلك العلمي؟
- ☐ امي
- ☐ اساسي (صف عاشر)
- ☐ ثانوي (توجيهي)
- ☐ جامعي (بكالوريوس)
- ☐ تعليم عالي (ماجستير او دكتوراه)

التاريخ المرضي

منذ متى و انت مشخص بالزرق (المياه الزرقاء) ؟ [____________________]

منذ متى و انت تستخدم قطرات العين ؟ [____________________]

كم عدد المرات الي تستخدم فيها قطرت العين في اليوم الواحد؟ [____________________]

كم مرة تزور فيها عيادات العيون في الشهر؟ [____________________]

كم مرة تزور فيها عيادات العيون في السنة ؟ [____________________]

كم عدد الادوية التي تستخدمها من اجل علاج الزرق ؟ [____________________]

كم عدد العمليات اجريتها من اجل علاج الزرق ؟ [____________________]

هل تعرف أحد من أفراد عائلتك أو أصدقائك المقربين لديهم داء الزرق ؟ [____________________]

هل يوجد لديك امراض سابقة؟
- ☐ سكري
- ☐ ضغط
- ☐ امراض القلب
- ☐ امراض تنفسية
- ☐ اكتئاب
- ☐ امراض مفاصل
- ☐ صداع نصفي (شقيقة)

هل انت مدخن؟

معرفتك بمرض داء الزرق (المياه الزرقاء)

		موافق بشدة	موافق	لا اعرف	غير موافق	غير موافق بشدة
١.	الزرق مرض يصيب العينين دون باقي أعضاء الجسم	☐	☐	☐	☐	☐
٢.	الزرق هو نتيجة ارتفاع في ضغط العين	☐	☐	☐	☐	☐
٣.	الزرق يؤدي الى اعتلال في العصب البصري و اضطرابات بصرية	☐	☐	☐	☐	☐
٤.	الزرق لديه أكثر من نوع (مثال: *زرق مفتوح الزاوية, زرق مغلق الزاوية, ثانوي, تطوّري..*)	☐	☐	☐	☐	☐
٥.	الزرق يمكن توارثه في العائلة	☐	☐	☐	☐	☐
٦.	تزيد فرصة حدوث الزرق مع التقدم في العمر	☐	☐	☐	☐	☐
٧.	ارتفاع ضغط الدم و السكري يزيدان من احتمالية الاصابة بالزرق	☐	☐	☐	☐	☐
٨.	الأشخاص الذين لديهم طول أو قصر في النظر أكثر عرضة للإصابة بالزرق	☐	☐	☐	☐	☐
٩.	استخدام قطرات العين التي تحتوي على الكورتيزون قد تسبب الزرق	☐	☐	☐	☐	☐
١٠.	استخدام أدوية أخرى غير قطرات العين قد يكون له تأثير على ضغط العين	☐	☐	☐	☐	☐
١١.	اعتقد ان التوتر والإجهاد قد يجعلان الزرق أسوأ	☐	☐	☐	☐	☐
١٢.	اعتقد ان استعمال الكومبيوتر قد يجعل الزرق أسوأ	☐	☐	☐	☐	☐
١٣.	اعتقد ان استعمال الهاتف الذكي (التلفون) قد يجعل الزرق أسوأ	☐	☐	☐	☐	☐
١٤.	اعتقد ان ضوء الفلورسنت قد يجعل الزرق أسوأ	☐	☐	☐	☐	☐
١٥.	اعتقد ان القراءة لفترات طويلة قد تجعل الزرق أسوأ	☐	☐	☐	☐	☐
١٦.	يجب معالجة الارتفاع في ضغط العين إن وجد	☐	☐	☐	☐	☐
١٧.	يمكن السيطرة على الزرق من خلال العلاج	☐	☐	☐	☐	☐
١٨.	الكشف والعلاج المبكر لا يستطيع ان يحد من تقدم مرض الزرق	☐	☐	☐	☐	☐
١٩.	من المحتمل خسارة الرؤية بشكل كامل نتيجة علاج الزرق بالليزر أو الجراحة	☐	☐	☐	☐	☐
٢٠.	سيكون استخدام قطرات العين زائدا عن الحاجة في حال اجراء علاج بالليزر او عملية جراحية لداء الزرق	☐	☐	☐	☐	☐
٢١.	زيارات المتابعة في العيادة ليست ضرورية بعد اجراء علاج بالليزر او عملية جراحية لداء الزرق	☐	☐	☐	☐	☐
٢٢.	الفحوصات والزيارات الدورية المنتظمة ليست ضرورية لمرضى الزرق	☐	☐	☐	☐	☐
٢٣.	من اللازم فحص المحيط البصري حتى وإن كان ضغط العين مسيطر عليه	☐	☐	☐	☐	☐
٢٤.	يجب على المريض إبلاغ طبيب العيون بكافّة الأدوية التي يتناولها	☐	☐	☐	☐	☐
٢٥.	يجب على المريض إبلاغ طبيب العيون بكافة الأمراض التي يعاني منها	☐	☐	☐	☐	☐
٢٦.	علاج الزرق يستمر مدى الحياة	☐	☐	☐	☐	☐
٢٧.	يتم استرجاع ما فُقِد من البصر بعد البدء بأخذ العلاجات لداء الزرق	☐	☐	☐	☐	☐

٢٨. **ما هي اعتقاداتك اتجاه علامات و اعراض الزرق (المياه الزرقاء)**
- ☐ عادة ما يكون بلا أعراض
- ☐ انخفاض في درجة وضوح الرؤية
- ☐ تضيق المجال البصري
- ☐ فقدان البصر
- ☐ اضطرابات كهربائية في الدماغ (نوبات)
- ☐ ألم واحمرار في العين، غثيان وتقيؤ عند التعرض إلى زرق ضيق الزاوية

٢٩. **خيارات علاج الزرق (اليمياه الزرقاء) تشمل:**
- ☐ ادوية
- ☐ الليزر
- ☐ عمليات جراحية

٣٠. **اختر الأعراض الجانبية الناتجة عن استخدام قطرات العين في علاج الزرق**
- ☐ شعور بالوخز والحرقة بالعينين
- ☐ عدم وضوح في الرؤية بعد التقطير
- ☐ تغير لون قزحيّة العين
- ☐ ضيق في التنفس
- ☐ تباطؤ في نبضات القلب
- ☐ زيادة طول رموش العينين
- ☐ حكة و احمرار و جفاف في جفن العين

٣١. **ما هو مصدر معلوماتك عن داء الزرق**
- ☐ طبيب العيون
- ☐ اطباء في تخصصات اخرى
- ☐ العائلة و الأصدقاء
- ☐ وسائل الإعلام (تلفاز، جرائد)
- ☐ شبكة الإنترنت
- ☐ وسائل التواصل الاجتماعي

٣٢. **ما هي الصعوبات البصرية التي تواجهها في الحياة اليومية بسبب داء الزرق (المياه الزرقاء)**
- ☐ تحديد هويّة الأشخاص في الخارج
- ☐ القراءة
- ☐ استخدام الدرج
- ☐ مشاهدة التّلفاز
- ☐ قيادة السّيّارة
- ☐ وسائل التواصل الاجتماعي
- ☐ المشي وحيدا في الخارج
- ☐ رؤية الألوان واختيار الملابس
- ☐ لا أجد أي صعوبة

٣٣. **جوانب تشعر بأنها يجب أن تتحسن في المعرفة والتوعية بمرض الزرق:**
- ☐ تحسين المعلومات المتعلقة بالمرض نفسه
- ☐ تحسين المعلومات المتعلقة بالخيارات العلاجية
- ☐ تحسين التّواصل الاجتماعي
- ☐ متابعة المشاعر السلبية المتعلقة بالمرض بشكل أفضل
- ☐ تقديم نصائح عملية بشكل أفضل
- ☐ لا شيء

٣٤. **ساهمت العملية الجراحية أو الليزر في جعل حالتي المرضيّة (إن أُجرِي أيّ منها):**
- ☐ أفضل
- ☐ أسوأ
- ☐ لم يحدث أي تغيير
