# Supplementary material for: Predictors of glaucoma knowledge and its risk factors among Jordanian patients with primary open angle glaucoma at a tertiary teaching hospital: A cross-sectional survey
Source: PLoS One. 2023 May 18;18(5):e0285405. doi: 10.1371/journal.pone.0285405 (PMC10194903; doi:10.1371/journal.pone.0285405)
Supplement: Attachment — Submitted filename: highlighted review.pdf [file pone.0285405.s005.pdf]

PLOS ONE

Predictors of glaucoma knowledge and its risk factors among Jordanian patients with primary open angle glaucoma at a tertiary teaching hospital: a cross-sectional survey

--Manuscript Draft--

**Manuscript Number:**	PONE-D-22-22392R1
**Article Type:**	Research Article
**Full Title:**	Predictors of glaucoma knowledge and its risk factors among Jordanian patients with primary open angle glaucoma at a tertiary teaching hospital: a cross-sectional survey
**Short Title:**	Predictors of glaucoma knowledge among Jordanian patients
**Corresponding Author:**	Abdallah Al-Ani<br>King Hussein Cancer Center<br>Amman, 'Ammān JORDAN
**Keywords:**	Glaucoma; Jordan; awareness; compliance; Education
**Abstract:**	Purpose of study: To assess the glaucoma knowledge and adherence to care seeking behaviors among Jordanian patients with glaucoma to formulate policies and compliance assuring strategies.<br>Methods: A cross-sectional survey was developed after an extensive literature search to investigate glaucoma-related knowledge among participants with glaucoma visiting the Jordan University Hospital clinics from October 2021 to February 2022. Responses were compared to a sample of ophthalmic participants with eye conditions other than glaucoma visiting the ophthalmology clinics at the same time frame.<br>Results: A total of 256 participants filled out the survey, of which 53.1% were diagnosed with glaucoma while 46.9% had ophthalmic conditions other than glaucoma. Our sample of participants is characterized by a mean age of 52.2 ± 17.8 years and a male-to-female ratio of 1.04:1. Overall, participants with glaucoma were more aware of their disease than participants with other ophthalmic conditions. Compared to their ophthalmic non-glaucoma counterparts, those diagnosed with glaucoma face significantly more daily life difficulties due to their ophthalmic disease (p <0.001). Results of the independent sample t-test demonstrate that participants with glaucoma have significantly higher knowledge scores (p <0.001) and were able to recognize more glaucoma symptoms than their non-glaucoma counterparts (p = 0.002). Similarly, those with a positive family history of glaucoma displayed higher knowledge (p = 0.005). Multivariate linear regression demonstrates that family history of glaucoma, higher symptom recognition score, reliance on ophthalmologists, and the internet for glaucoma-related information are positive predictors of higher knowledge scores.<br>Conclusion: We have demonstrated that ophthalmic patients display mediocre levels of glaucoma knowledge. Raising awareness through various interventions may improve the lifestyles of patients with glaucoma and alleviate the economic burden associated with treating the disease.
**Order of Authors:**	Sana' Muhsen
	Leen Al-Huneidy
	Ward Maaita
	Lina AlQirem
	Zaid Madain
	Jaleel Sweis
	Raya Abu Tawileh
	Yazan Al-Huneidy
	Amro Alkhatib
	Abdallah Al-Ani
**Response to Reviewers:**	Rebuttal Letter

Powered by Editorial Manager® and ProduXion Manager® from Aries Systems Corporation

On behalf of myself and all authors within this study, we thank the panel of reviewers for their extensive, shrewd, and informative comments. Any and all changes requested implied through your comments were considered and worked upon in order to strengthen the framework of our manuscript and render it suitable for publication in your respected journal. We would like to thank you for all of your efforts. The new manuscript file now has an expanded introduction, methodology, results, and discussion sections, all based on your comments. We hope that these expansions address your concerns and further validate our work. We immensely appreciate giving us the opportunity to revise our manuscript. We would like to thank the editorial team and the associated reviewers for their efforts. We hope that everyone is staying safe and well.

Editorial comments
1.Please ensure that your manuscript meets PLOS ONE's style requirements, including those for file naming.
a.Response: The manuscript style was edited to match PLOS ONE’s style requirements.
2.Please provide additional details regarding participant consent. In the ethics statement in the Methods and online submission information, please ensure that you have specified (1) whether consent was informed and (2) what type you obtained (for instance, written or verbal, and if verbal, how it was documented and witnessed). If your study included minors, state whether you obtained consent from parents or guardians. If the need for consent was waived by the ethics committee, please include this information.
a.Response: The ethical considerations section was updated to accommodate for the abovementioned concerns. The methodology section states that only participants 18 years and older were recruited; therefore, no minors were included in the study.
3.You indicated that you had ethical approval for your study. Please clarify whether minors (participants under the age of 18 years) were included in this study. If yes, in your Methods section, please ensure you have also stated whether you obtained consent from parents or guardians of the minors included in the study or whether the research ethics committee or IRB specifically waived the need for their consent.
a.Response: The ethical considerations section was updated to accommodate for the abovementioned concerns. The methodology section states that only participants 18 years and older were recruited; therefore, no minors were included in the study.
4.Please review your reference list to ensure that it is complete and correct. If you have cited papers that have been retracted, please include the rationale for doing so in the manuscript text, or remove these references and replace them with relevant current references. Any changes to the reference list should be mentioned in the rebuttal letter that accompanies your revised manuscript. If you need to cite a retracted article, indicate the article’s retracted status in the References list and also include a citation and full reference for the retraction notice.
a.Response: The reference list was checked and the any added references resulted from dealing with reviewers’ comments were noted at in the rebuttal letter.

Reviewer comments

Abstract
1.Why compare to ophthalmic patients? this demography is expected to demonstrate some knowledge a eye patients. so maybe the public at large would be a better option especially considering the use of self administered questionnaire
a.Response: Dear respected reviewer, the data collection was designed in such a fashion in order to concentrate efforts into the recruitment of patients within one space, that is the ophthalmology clinics. Interestingly enough, glaucoma patients demonstrated far superior knowledge relative to the ophthalmic patients, which could only let us speculate about that of the general public. In fact, an examination of the general public and their glaucoma knowledge is the phase II of this project.
2.Name of the test should be mentioned here. Like regression, correlation etc
a.Response: Type of statistical test was added to the abstract.

Introduction
3.Some explanation here as advanced age would be expected to have more knowledge? especially as it is a disease of older people usually
a.Response: We thank the reviewer for raising such an important point. Explanation to

this point was added to the discussion as not to congest the introduction. The following was added: “Interestingly, age was predominantly a negative predictor of glaucoma knowledge within the literature. A variety of factors could’ve influenced such relationship such as the low levels of health literacy in older individuals, neurological disease progression, or the inherent technological bias exhibited by younger participants able to fully utilize the internet to gain disease-related knowledge [34,44].”
4.Some explanation here as well
a.Response: The statement was altered to showcase more clarity. The statement now reads: “Subsequently, those poor clinical presentations are associated with worse treatment outcomes as cases might be treated with more complex surgical options to halt the rapidly progressive and irreversible retinal damage [5,14].”
5.Difference between knowledge and awareness should be made clear
a.Response: We thank the review for their keen comment. The following was added to the introduction: “The literature evaluating glaucoma knowledge within the Middle East and therefore its treatment implications, is scarce. In fact, an accurate estimation of knowledge is tricky to calculate due to the juxtaposition with the concept of awareness. It should be noted that awareness refers to the generalized or diffuse knowledge of a particular concept (e.g., have you heard of glaucoma?), while knowledge refers to any information that is factual in nature (e.g., prevalence, etiology, risk factors, etc.)[16].”

Methodology
6.self-administered questionnaire should be enough, also since this includes patients, an interview with questionnaire could have been a better option
a.Response: We appreciate the reviewer’s keen points. Patients were given the questionnaire and a team of specialized data collectors contacted the patients to ensure that they understood all items provided in the questionnaire. In fact, some older patients with severe visual disability needed the questionnaire to be read for them as they couldn’t read it on their own.
7.Define type of glaucoma, how did you diagnose glaucoma.just mention e.g oct VF etc also exclude other types of glaucoma such as secondary causes
a.Response: The target population in question were those with primary glaucoma. Since closed-angle glaucoma is a medical emergency, it is only logical that all recruited patients had chronic primary open angle glaucoma. Diagnostic modality and an expanded exclusion criteria were added. The new paragraph reads: “Patients were enrolled into two groups: The first group consisted of participants with a clinically proven diagnosis of primary glaucoma of at least 6 months and are on medications. Diagnosis of glaucoma was established on Optical Coherence Tomography. Patients with secondary glaucoma (e.g., pseudoexfoliation syndrome, pigment dispersion syndrome, neovascular glaucoma, inflammatory glaucoma, and medication-induced glaucoma) were excluded. The second group of participants consisted of those who were diagnosed with any vision hindering ophthalmic conditions with the exception of long-standing glaucoma. Such conditions include cataracts, diabetic retinopathy, macular degeneration, optic neuritis among others. Participants that were younger than 18 years of age, suffer from mental/psychiatric illnesses, and are unable to consent to participation in the survey were excluded from the study. Moreover, participants that failed to complete at least 80% of the questionnaire were also excluded.”
8.vision hindering? define term..is it vision threating or just routine patients
a.Response: The term was illuminated on with the use of examples.
9.exclusion and inclusion should be more specific..like glaucoma patients should not have other pathology etc
a.Response: Refer to response to comment number 7
10.Non mutually exclusive? statement 28 through 30 not included in document so cant comment
a.Response: The full questionnaire is now added to the revised submission.
11.any scoring system or just simple addition? was reverse coding utilised as well? as these were self administered questionnaires. without interview, some pariticipants might have filled in a hurry. Reverse coding might have hinted at that
a.Response: Dear respected reviewer, the scoring was a simple addition of all correct items. The knowledge score wasn’t even grouped into poor or good knowledge as such grouping would require validation and diagnostic analysis by ROC curves which outside the scope of this study. As for the reverse coding, as the reviewer knows, participants, especially those with vision ailments, are very sensitive to the number of questions in a questionnaire. Therefore, if reverse-coding was used, a higher rate of participant attrition would occur. However, to combat the issue of participants finishing

Powered by Editorial Manager® and ProduXion Manager® from Aries Systems Corporation

	the questionnaire in a hurry, we implemented the following: 1) providing negative and positive statements throughout the questionnaire, 2) calculating the mean time to finish among participants which was about 8 minutes.<br>12.can be used for continuous data e.g mean between 2 groups, for categorical use chi square<br>a.Response: The addition of (for categorical variables with more than two groups) next to the ANOVA test and the t-test was in reference to the INDEPDENT variable. Indeed, the ANOVA and t-test are tests of mean differences by which difference in a dependent continuous variable is tested among 2 independent groups (t-test) or 3+ independent groups (ANOVA). Therefore, to avoid this confusion that we may caused, the brackets were removed.<br>13.Please confirm this mean is taken from continuuous data, and not categorical data in spss, if it is categorical and we take mean it will be wrong. Just a heads up<br>a.Response: All data presented as means ± SD were taken from continuous variables. Within the questionnaire number of medications, number of drops per day, and number of surgeries were asked.<br>14.mention which test is used here not just p value<br>a.Response: The test used here was the chi squared test. The notion (X2(df) = X, p-value) denotes chi-square and was added accordingly.<br>15.elaborate on seizures?<br>a.Response: Simply, we asked participants on what they thought are symptomatic complications of glaucoma. Options were reduced visual acuity, narrow visual field, vision loss, convulsive seizures, and symptoms of inflammation. Convulsive seizures refer to whole body seizures (grand mal seizures).<br>16.open or closed angle?these symptoms are usually in closed angle glaucoma , which might mean a difference in attitude towards adherence as it is more painful etc<br>a.Response: Indeed, such symptoms denote a diagnosis of a closed-angle glaucoma. However, participants were asked these questions to test their knowledge of primary glaucoma including both closed and open angle.<br>17.mention which test is used here. it canot be t test<br>a.Response: Indeed, each category was compared amongst the ophthalmic and glaucoma patients using the chi-square test. Mentioning of the chi-square test will be added in the footnote of the table.<br>18.which type of regression is used please mention here<br>a.Response: Dear respected reviewer, univariate analysis within the context of this paragraph refers to either t-test or ANOVA and not regression (which is a form of multivariate analysis). Continuous variables such as knowledge score, symptom recognition, and adverse effect recognition were examined for difference between 2 groups using the t-test (e.g., biological sex, disease status, or family history of glaucoma), and the between 3 or more groups using the ANOVA test (e.g., residence, education, or income). The notion of (t(df) = X, p-value) denotes the use of the student t-test and the notion of (F(df between groups, df within groups) = X, p-value) denotes the use of the ANOVA per APA guidelines. Both were added accordingly (when applicable).<br>19.linear regression cannot be used for categorical variables unless a dummy variable is made as a reference....or use logistic regression if the outcome is binary which again required reference variable...here the dummy or reference variable is not shown<br>a.Response: The reviewer is correct with their knowledge regarding linear regression; however, the linear regression model is found in table 5 and not table 4. Table 4 are mere univariate analysis consisting of t-test and ANOVA as described in the response above.<br>20.again family history i.e yes or no is categorical, either use dummy variable and show which is the reference variable<br>a.Response: Dear esteemed reviewer, indeed, family history is a categorical variable. The dummy variable in the model was no family history of glaucoma. The same applies for gender, smoking status (yes/no), and presence of comorbidities. Table 5, which contains the model is updated to contain the reference variables and reduce any confusion to readers.<br>21.Try to fit univariate and multivariate in 1 table....only show the significant results in the table to make it smaller. This allows easy comparison<br>a.Response: We appreciate the reviewer's concern with that regard. However, table 4 (table of univariate analysis) has 3 outcome measures (i.e., knowledge, symptom recognition, and adverse effect recognition scores), while table 5 (table of multivariate analysis) has only knowledge as its outcome variable. Therefore, combining the two

	tables would create a congested mess of numbers that might be harder to consume by the readers.<br><br>Discussion<br>22.This part might be included introduction to avoid redundancy<br>a.Response: We thank the reviewer for raising such point. That part of the discussion was reorganized and place within the introduction.<br>23.how much % of glaucoma patients were aware? since the general population is aware 38.8% , it should be more than this theoretically<br>a.Response: Dear respected reviewer, indeed, knowledge of glaucoma should be higher among glaucoma patients than the general populace. However, the report which describes glaucoma knowledge among patients visiting Jordan University Hospital did not stratify knowledge per diagnosis. In fact, in their sample of 488, only 20 participants were diagnosed with glaucoma (Refer to: 10.4103/1319-4534.322618)<br>24.please clear the difference in introduction between awareness and knowledge as it seems to be mixed here/ confusing for the reader. Define it in objectives of study perhaps<br>a.Response: The following was added to the introduction to illuminate on the differences between awareness and knowledge: “The literature evaluating glaucoma knowledge within the Middle East and therefore its treatment implications, is scarce. In fact, an accurate estimation of knowledge is tricky to calculate due to the juxtaposition with the concept of awareness. It should be noted that awareness refers to the generalized or diffuse knowledge of a particular concept (e.g., have you heard of glaucoma?), while knowledge refers to any information that is factual in nature (e.g., prevalence, etiology, risk factors, etc.)[16].”<br>25.in my opinion theres is little need for mentioning so many countries.Just local studies and few western studies should be enough perhaps<br>a.Response: We thank the reviewer for their keen concern. We believe that the inclusion of such studies serves two purposes. Firstly, it shows that variability between country and continents in terms of glaucoma knowledge and/or awareness. Secondly, it serves to provide future researchers and readers with a significant body of literature that they can explore, refer to, and even expand within the context of our results. If the reviewer strongly believes that a reduction should be made, then we are more than happy to comply.<br>26.just 2 or 3 references should be enough<br>a.Response: The number of references was reduced to 3 per the reviewer’s advice.<br>27.Generally the discussion section is redundant in many places, confusing the reader.In my humble opinion it needs to be written coherently , in a stepwise manner and not repeat what has already been established in previous paragraphs. Also there seems to be a few grammatical mistakes. This can be solved by using software such as 'Grammarly'. Of course, Statistical analysis and results section first should be assured free of errors.<br>a.Response: Dear esteemed reviewer, all points raised with regards to the statistical analysis and results were addressed. Type of statistical testing was shown (resulted with expanded to show such a change) and every part of the analysis was described throughout our responses and changes within the manuscript. As for the discussion, it went through a grammatical check by a native English speaker and we cut at certain parts to avoid redundancy whenever applicable. The new discussion is now presented in the following themes: 1) Summary of findings, 2) Comparison with local literature, 3) Comparison with Western literature, 4) Illuminating on the reasons for variance between different countries/studies, 5) Factors associated with glaucoma knowledge, 6) Recommendations for better glaucoma knowledge and awareness, 7) Limitations.<br>28.Unclear ? please rephrase if possible<br>a.Response: The statement aims to stress on the fact that whatever educational intervention that will be given through concerned authorities, it must account that glaucoma have various types and might require different treatments for different risk groups. Therefore, an intervention must be clear, concise, and accommodating. To avoid redundancy, the statement was removed.
Additional Information:	
Question	Response
**Financial Disclosure**	The author(s) received no specific funding for this work.

Powered by Editorial Manager® and ProduXion Manager® from Aries Systems Corporation

Enter a financial disclosure statement that describes the sources of funding for the work included in this submission. Review the submission guidelines for detailed requirements. View published research articles from *PLOS ONE* for specific examples.<br><br>This statement is required for submission and **will appear in the published article** if the submission is accepted. Please make sure it is accurate.<br><br>**Unfunded studies**<br>Enter: *The author(s) received no specific funding for this work.*<br><br>**Funded studies**<br>Enter a statement with the following details:<br>• Initials of the authors who received each award<br>• Grant numbers awarded to each author<br>• The full name of each funder<br>• URL of each funder website<br>• Did the sponsors or funders play any role in the study design, data collection and analysis, decision to publish, or preparation of the manuscript?<br>• **NO** - Include this sentence at the end of your statement: *The funders had no role in study design, data collection and analysis, decision to publish, or preparation of the manuscript.*<br>• **YES** - Specify the role(s) played.<br><br>* typeset	
**Competing Interests**<br><br>Use the instructions below to enter a competing interest statement for this submission. On behalf of all authors, disclose any competing interests that could be perceived to bias this work—acknowledging all financial support and any other relevant financial or non-financial competing interests.<br><br>This statement is required for submission and **will appear in the published article** if the submission is accepted. Please make	The authors have declared that no competing interests exist.

sure it is accurate and that any funding sources listed in your Funding Information later in the submission form are also declared in your Financial Disclosure statement.<br><br>View published research articles from *PLOS ONE* for specific examples.<br><br>**NO authors have competing interests**<br><br>Enter: *The authors have declared that no competing interests exist.*<br><br>**Authors with competing interests**<br><br>Enter competing interest details beginning with this statement:<br><br>*I have read the journal's policy and the authors of this manuscript have the following competing interests: [insert competing interests here]*<br><br>* typeset	
**Ethics Statement**<br><br>Enter an ethics statement for this submission. This statement is required if the study involved:<br><br>• Human participants<br>• Human specimens or tissue<br>• Vertebrate animals or cephalopods<br>• Vertebrate embryos or tissues<br>• Field research<br><br>Write "N/A" if the submission does not require an ethics statement.<br><br>General guidance is provided below. Consult the submission guidelines for detailed instructions. **Make sure that all information entered here is included in the Methods section of the manuscript.**	The study's protocol was approved by the Jordan University Hospital Institutional Review Board (ID:212/2022/09) and was issued on the 1st of September 2021 by the aforementioned body. All participants have read and signed an informed written consent form before continuing to complete the questionnaire. The consent form included the participants' right to anonymity, confidentially of their data, right to leave the study, and reassurance that their participation is completely voluntary, is not associated with any kind of short-term benefit or rewards, and does not affect the quality of their received care.

Powered by Editorial Manager® and ProduXion Manager® from Aries Systems Corporation

**Format for specific study types**<br><br>**Human Subject Research (involving human participants and/or tissue)**<br>• Give the name of the institutional review board or ethics committee that approved the study<br>• Include the approval number and/or a statement indicating approval of this research<br>• Indicate the form of consent obtained (written/oral) or the reason that consent was not obtained (e.g. the data were analyzed anonymously)<br><br>**Animal Research (involving vertebrate animals, embryos or tissues)**<br>• Provide the name of the Institutional Animal Care and Use Committee (IACUC) or other relevant ethics board that reviewed the study protocol, and indicate whether they approved this research or granted a formal waiver of ethical approval<br>• Include an approval number if one was obtained<br>• If the study involved *non-human primates*, add *additional details* about animal welfare and steps taken to ameliorate suffering<br>• If anesthesia, euthanasia, or any kind of animal sacrifice is part of the study, include briefly which substances and/or methods were applied<br><br>**Field Research**<br><br>Include the following details if this study involves the collection of plant, animal, or other materials from a natural setting:<br>• Field permit number<br>• Name of the institution or relevant body that granted permission	
**Data Availability**<br><br>Authors are required to make all data underlying the findings described fully available, without restriction, and from the time of publication. PLOS allows rare exceptions to address legal and ethical concerns. See the PLOS Data Policy and FAQ for detailed information.	Yes - all data are fully available without restriction

Powered by Editorial Manager® and ProduXion Manager® from Aries Systems Corporation

A Data Availability Statement describing where the data can be found is required at submission. Your answers to this question constitute the Data Availability Statement and **will be published in the article**, if accepted.<br><br>**Important:** Stating 'data available on request from the author' is not sufficient. If your data are only available upon request, select 'No' for the first question and explain your exceptional situation in the text box.<br><br>Do the authors confirm that all data underlying the findings described in their manuscript are fully available without restriction?	
**Describe where the data may be found in full sentences. If you are copying our sample text, replace any instances of XXX with the appropriate details.**<br><br>• If the data are **held or will be held in a public repository**, include URLs, accession numbers or DOIs. If this information will only be available after acceptance, indicate this by ticking the box below. For example: *All XXX files are available from the XXX database (accession number(s) XXX, XXX.*).<br>• If the data are all contained **within the manuscript and/or Supporting Information files**, enter the following: *All relevant data are within the manuscript and its Supporting Information files.*<br>• If neither of these applies but you are able to provide **details of access elsewhere**, with or without limitations, please do so. For example:<br><br>*Data cannot be shared publicly because of [XXX]. Data are available from the XXX Institutional Data Access / Ethics Committee (contact via XXX) for researchers who meet the criteria for access to confidential data.*<br><br>*The data underlying the results presented in the study are available from (include the name of the third party*	All relevant data are within the manuscript and its Supporting Information files.

and contact information or URL).<br>• This text is appropriate if the data are owned by a third party and authors do not have permission to share the data.<br><br>* typeset	
Additional data availability information:	

Powered by Editorial Manager® and ProduXion Manager® from Aries Systems Corporation

Manuscript

Click here to access/download;Manuscript;Final Manuscript_R1_Accepted.docx

Predictors of glaucoma knowledge and its risk factors among Jordanian patients with primary open angle glaucoma at a tertiary teaching hospital: a cross-sectional survey

Authors:

Sana' Muhsen[1*], Leen Al-Huneidy[2], Ward Maaita[2], Lina AlQirem[2], Zaid Madain[2], Jaleel Sweis[2], Raya Abu Tawileh[2], Yazan Al-Huneidy[2], Amro Alkhatib[2], Abdallah Al-Ani[2,3]

Affiliations:

[1]Special Surgery Department/Ophthalmology Division, School of Medicine, University of Jordan, Amman, Jordan

[2]School of Medicine, University of Jordan, Amman, Jordan

[3]Office of Scientific Affairs and Research, King Hussein Cancer Center, Amman, Jordan

*** Corresponding author**:

E-mail: sana_mohsen79@yahoo.com

Funding: This work has received no funding

Abstract

Purpose of study: To assess the glaucoma knowledge and adherence to care seeking behaviors among Jordanian patients with glaucoma to formulate policies and compliance assuring strategies.

Methods: A cross-sectional survey was developed after an extensive literature search to investigate glaucoma-related knowledge among participants with glaucoma visiting the Jordan University Hospital clinics from October 2021 to February 2022. Responses were compared to a sample of ophthalmic participants with eye conditions other than glaucoma visiting the ophthalmology clinics at the same time frame.

Results: A total of 256 participants filled out the survey, of which 53.1% were diagnosed with glaucoma while 46.9% had ophthalmic conditions other than glaucoma. Our sample of participants is characterized by a mean age of 52.2 ± 17.8 years and a male-to-female ratio of 1.04:1. Overall, participants with glaucoma were more aware of their disease than participants with other ophthalmic conditions. Compared to their ophthalmic non-glaucoma counterparts, those diagnosed with glaucoma face significantly more daily life difficulties due to their ophthalmic disease ($p < 0.001$). Results of the independent sample *t*-test demonstrate that participants with glaucoma have significantly higher knowledge scores ($p < 0.001$) and were able to recognize more glaucoma symptoms than their non-glaucoma counterparts ($p = 0.002$). Similarly, those with a positive family history of glaucoma displayed higher knowledge ($p = 0.005$). Multivariate linear regression demonstrates that family history of glaucoma, higher symptom recognition score, reliance on ophthalmologists, and the internet for glaucoma-related information are positive predictors of higher knowledge scores.

Conclusion: We have demonstrated that ophthalmic patients display mediocre levels of glaucoma knowledge. Raising awareness through various interventions may improve the

lifestyles of patients with glaucoma and alleviate the economic burden associated with treating the disease.

Keywords: Glaucoma; Jordan; Awareness; Compliance; Education

Introduction

Glaucoma encompasses a spectrum of diseases characterized by degeneration of the retinal ganglion cells leading to optic disc damage and irreversible vision loss [1]. Being the second most common cause of blindness worldwide, glaucoma affected 64.3 million adults in 2013 and is expected to affect 111.8 million by 2040 [2,3]. Vision loss associated with glaucoma can be prevented with early diagnosis and treatment; however, due to its asymptomatic nature, lack of awareness of risk, and low levels of referral, the incidence of glaucoma is rising within the population [4]. In addition, the chronicity of the disease implies that patients with glaucoma may be subjected to various quality of life and therapeutic concerns due to long-term noncompliance, gender discrimination, and health literacy, all of which are influenced by the patients' awareness towards their disease [4,5].

Despite the discrepancies between developed and developing nations in terms of knowledge of patients with glaucoma [6,7], the overall level of knowledge is poor [8,9]. Throughout the literature, factors such as socioeconomic status, short disease duration, and advanced age were negative predictors of glaucoma knowledge [8,10–12]. Poor glaucoma knowledge influences the clinical progression of the disease as it contributes to a lack of adherence to palliative drug regimes, and the underestimation of regular ophthalmic examinations [2,5,13]. Subsequently, those poor clinical presentations are associated with worse treatment outcomes as cases might be treated with more complex surgical options to halt the rapidly progressive and irreversible retinal damage [5,14]. Therefore, knowledge of glaucoma could be of vital importance as a target of interventions aiming to improve the lifestyles among those with glaucoma, particularly high-risk groups (e.g., geriatrics). Gatwood et al., (2022) demonstrate that disease-related educational interventions improve adherence to glaucoma treatment [15].

The literature evaluating glaucoma knowledge within the Middle East and therefore its treatment implications, is scarce. In fact, an accurate estimation of knowledge is tricky to calculate due to the juxtaposition with the concept of awareness. It should be noted that awareness refers to the generalized or diffuse knowledge of a particular concept (e.g., have you heard of glaucoma?), while knowledge refers to any information that is factual in nature (e.g., prevalence, etiology, risk factors, etc.)[16].

Among Jordanians, glaucoma is the third leading cause of severe bilateral blindness [17]. Glaucoma awareness throughout the population was estimated to be approximately 38.8% [18]. However, there exist no reports evaluating glaucoma knowledge among Jordanians. Thus, this study aims to assess the glaucoma knowledge and adherence to care seeking behaviors among Jordanian patients with glaucoma to formulate relevant educational policies and compliance assuring strategies.

Material and methods

We conducted this cross-sectional study using a self-administered questionnaire on patients visiting the ophthalmology clinics at Jordan University Hospital from October 2021 to February 2022. The Jordan University Hospital is the largest tertiary and academic health provider in Jordan. In addition, it is the largest referral center for all of central Jordan serving over four million patients.

Patients were enrolled into two groups: The first group consisted of participants with a clinically proven diagnosis of primary open angle glaucoma of at least 6 months and are on medications. Diagnosis of glaucoma was established on Optical Coherence Tomography. Patients with secondary glaucoma (e.g., pseudoexfoliation syndrome, pigment dispersion syndrome, neovascular glaucoma, inflammatory glaucoma, and medication-induced

glaucoma) were excluded. The second group of participants consisted of those who were diagnosed with any vision hindering ophthalmic conditions with the exception of long-standing glaucoma. Such conditions include cataracts, diabetic retinopathy, macular degeneration, and optic neuritis among others. Participants that were younger than 18 years of age, suffer from mental/psychiatric illnesses, and are unable to consent to participation in the survey were excluded from the study. Moreover, participants that failed to complete at least 80% of the questionnaire were also excluded. Systemic random sampling was adopted to recruit every 4th patient from the ophthalmology clinics.

Instrument development

The study's questionnaire was developed based on an extensive literature review of the most relevant articles reporting on the perceived knowledge of glaucoma by both patients with or without glaucoma [12,19–25]. The questionnaire is composed of the following sections: demographics (5 items), medical history (10 items), perceived glaucoma knowledge (31 items), and a miscellaneous section (4 items). The first 27 statements of the glaucoma knowledge section were presented as Likert-scales with 1 being strongly disagree and 5 being strongly agree. Statement 28 (signs and symptoms of glaucoma), statement 29 (treatment options for glaucoma), and statement 30 (complications of glaucoma treatment) were presented as non-mutually exclusive multiple choices. Miscellaneous items included statements exploring sources of glaucoma-related information, daily life difficulties due to ophthalmic conditions, ways on how to improve glaucoma awareness, and impact of surgical treatment on patients undergoing glaucoma surgery.

The questionnaire items were extracted from the literature, therefore, preserving their external validity. Furthermore, a panel of two expert ophthalmologists and four senior residents have approved the content of the questionnaire and slightly edited some of its

statements to fit ophthalmic practice at the Jordan University Hospital. A pilot test on 15 patients with glaucoma and 15 patients with ophthalmic disorders other than glaucoma yielded a Cronbach alpha of 0.704 for the 27 knowledge Likert-scales.

The questionnaire underwent back-to-back translation as it was translated from English to Arabic by an expert ophthalmologist and then back to English by an official translator in order to ensure the validity of the initial translation.

Statistical analysis

All statistical analyses and data cleaning were conducted on SPSS version 24 (SPSS Inc., Chicago, IL, USA). Categorical data were reported as frequencies (n (%)), while continuous data were presented as means ± standard deviations. Mean differences among different categories were examined by the independent sample *t*-test or ANOVA. Statistical associations between categorical variables were assessed using the chi-square test. Upon conducting chi-square testing, the 5-point Likert-scales were condensed into three categories (strongly disagree and disagree as one category; neutral as one category; strongly agree and agree as one category). Knowledge scores were computed by adding how many correct answers that participants had out of the first 27 knowledge items (1 point per each correct statement resulting in a total score of 27). Symptoms and adverse effects recognition scores were computed as the sum of participants' chosen categories. Pearson R correlation testing was conducted to assess the correlation between continuous variables and knowledge scores. Moreover, a multivariate linear regression model was computed to assess predictors of knowledge score. All statistical tests are conducted with a 95% confidence interval and a 5% error margin. A p-value of less than 0.05 is considered statistically significant.

Ethical considerations

The study’s protocol was approved by the Jordan University Hospital Institutional Review Board (ID:212/2022/09) and was issued on the 1st of September 2021 by the aforementioned body. All participants have read and signed an informed written consent form before continuing to complete the questionnaire. The consent form included the participants’ right to anonymity, confidentially of their data, right to leave the study, and reassurance that their participation is completely voluntary, is not associated with any kind of short-term benefit or rewards, and does not affect the quality of their received care.

Results

A total of 256 participants filled out the survey, of which 53.1% were diagnosed with glaucoma while 46.9% had ophthalmic conditions other than glaucoma. Our sample of participants is characterized by a mean age of 52.2 ± 17.8 years and a male to female ratio of 1.04:1. The majority of the sample resided in the capital city of Amman (66.8%), had a bachelor’s degree or higher (50.8%), and earned less than 500 JDs in monthly income (64.5%). Characteristics of participants with glaucoma were similar to those without glaucoma with the exception of age (p = 0.009), clinic visits per month (p <0.001), clinic visits per year (p <0.001), and family history of glaucoma (p = 0.006). **Table 1** describes the sociodemographic characteristics of the recruited cohort.

Table 1: Sociodemographic characteristics of the studied population

		Ophthalmic non-glaucoma patients (n = 120)	Glaucoma patients (n = 136)	p-value
Age in years	Mean ± SD (Median)	48.1 ± 16.4 (49.5)	54.6 ± 18.2 (57.0)	**0.009**
Clinic visits per month	Mean ± SD (Median)	0.5 ± 0.8 (0.0)	0.9 ± 0.7 (1.0)	**<0.001**
Clinic visits per year	Mean ± SD (Median)	3.6 ± 5.6 (2.0)	7.0 ± 6.8 (4.0)	**<0.001**
Biological Sex				0.133

	Male	55 (45.8)	76 (55.9)	
	Female	65 (54.2)	60 (44.1)	
Residence				0.832
	Amman (Capital)	80 (66.7)	91 (66.9)	
	Central	29 (24.2)	30 (22.1)	
	South	5 (4.2)	9 (6.6)	
	North	6 (5.0)	6 (4.4)	
Education				0.415
	Illiterate	8 (6.7)	12 (8.8)	
	Primary	10 (8.3)	21 (15.4)	
	Highschool	37 (30.8)	38 (27.9)	
	Bachelors	52 (43.3)	54 (39.7)	
	Master's +	13 (10.8)	11 (8.1)	
Income				0.091
	< 499	69 (57.5)	96 (70.6)	
	500 – 999	39 (32.5)	30 (22.1)	
	1000 – 1999	8 (6.7)	9 (6.6)	
	> 2000	4 (3.3)	1 (0.7)	
Comorbidities				0.133
	No	63 (52.5)	58 (42.6)	
	Yes	57 (47.5)	78 (57.4)	
Smoking history				0.373
	No	89 (74.2)	108 (79.4)	
	Yes	31 (25.8)	28 (20.6)	
Family history of glaucoma				**0.006**
	No	90 (77.6)	83 (61.5)	
	Yes	26 (21.7)	52 (38.5)	

Amongst participants with glaucoma, the mean time since glaucoma diagnosis was 9.7 ± 9.4 years, and the mean time since the start of glaucoma treating drops was 9.1 ± 8.7 years. Participants with glaucoma used a mean of 2.4 ± 1.4 medications and a mean of 3.3 ± 2.9 drops per day for the treatment and/or control of their glaucoma. Furthermore, participants were subjected to a mean of 1.6 ± 2.7 surgeries for the treatment of glaucoma in their lifetime.

Table 2 demonstrates the responses of participants towards glaucoma knowledge items. Overall, participants with glaucoma were more aware of their disease than participants with other ophthalmic conditions. Participants with glaucoma were significantly more likely than their non-glaucoma counterparts to recognize that glaucoma is a predominantly ophthalmic

disease ($X^2(2) = 11.16$, $p = 0.004$), is the result of increased intraocular pressure ($X^2(2) = 19.56$, $p < 0.001$), can progress to optic nerve damage ($X^2(2) = 29.41$, $p < 0.001$), and is more likely to increase in incidence with age ($X^2(2) = 9.13$, $p = 0.010$). Moreover, they were more likely to think that stress ($X^2(2) = 6.05$, $p = 0.048$), prolonged computer usage ($X^2(2) = 10.05$, $p = 0.007$), prolonged smartphone usage ($X^2(2) = 11.59$, $p = 0.003$), and prolonged reading ($X^2(2) = 9.15$, $p = 0.010$) may worsen symptoms of glaucoma. Compared to ophthalmic non-glaucoma participants, those diagnosed with glaucoma were familiar with the nature of their treatment as they are vigilant of the ability of medications to control glaucoma ($X^2(2) = 19.98$, $p < 0.001$), and that glaucoma treatment is lifelong ($X^2(2) = 32.97$, $p < 0.001$).

Nonetheless, both groups of participants portrayed variability in their awareness towards types of glaucoma, certain risk factors associated with glaucoma (i.e., myopia), effect of certain medications on glaucoma development (e.g., corticosteroid containing drops), and the post-operative implications of the surgical management of glaucoma.

Table 2: Responses of participants to knowledge items

	Ophthalmic non-glaucoma patients			Glaucoma patients			
**Items**	Disagree	Neutral	Agree	Disagree	Neutral	Agree	p-value
Glaucoma is a disease that affects the eyes and no other part of the body.	4 (3.3)	34 (28.3)	82 (68.3)	5 (3.7)	16 (11.8)	115 (84.6)	**0.004**
Glaucoma is caused by raised intraocular pressure.	7 (5.8)	54 (45.0)	59 (49.2)	7 (5.1)	27 (19.9)	102 (75.0)	**0.000**
Glaucoma can lead to optic nerve damage and visual disorders.	1 (0.8)	47 (39.2)	72 (60.0)	1 (0.7)	14 (10.3)	121 (89.0)	**0.000**
Glaucoma has more than one type (eg: open angle, closed angle, secondary, developmental)	3 (2.5)	92 (76.7)	25 (20.8)	3 (2.2)	93 (68.4)	40 (29.4)	0.290
Glaucoma can be inherited in the family.	19 (15.8)	55 (45.8)	46 (38.3)	25 (18.4)	47 (34.6)	64 (47.1)	0.182
Glaucoma is more common as you get older.	3 (2.5)	35 (29.2)	82 (68.3)	6 (4.4)	19 (14.0)	111 (81.6)	**0.010**
High blood pressure and diabetes increase the risk of glaucoma.	2 (1.7)	42 (35.0)	76 (63.3)	11 (8.1)	39 (28.7)	86 (63.2)	0.050
People with high myopia or hyperopia are more likely to get glaucoma.	20 (16.7)	58 (48.3)	42 (35.0)	16 (11.8)	72 (52.9)	48 (35.3)	0.507
The use of steroid /eye drops can cause glaucoma.	9 (7.5)	76 (63.3)	35 (29.2)	13 (9.6)	70 (51.5)	53 (39.0)	0.160
Medicines other than eye drops can influence intraocular pressure.	6 (5.0)	51 (42.5)	63 (52.5)	15 (11.0)	50 (36.8)	71 (52.2)	0.187
Stress can make glaucoma worse.	6 (5.0)	41 (24.2)	73 (60.8)	7 (5.1)	28 (20.6)	101 (74.3)	**0.048**
Using a computer will make glaucoma worse.	4 (3.3)	40 (33.3)	76 (63.3)	13 (9.6)	25 (18.4)	98 (72.1)	**0.007**
Using a smartphone will make glaucoma worse.	4 (3.3)	38 (31.7)	78 (65.0)	14 (10.3)	22 (16.2)	100 (73.5)	**0.003**
Fluorescent lights will make glaucoma worse.	10 (8.3)	59 (49.2)	51 (42.5)	20 (14.7)	62 (45.6)	53 (39.7)	0.286
A lot of reading may make glaucoma worse	11 (9.2)	47 (39.2)	62 (51.7)	13 (9.6)	30 (22.1)	93 (68.4)	**0.010**
High intraocular pressure must be addressed.	0 (0.0)	13 (10.8)	107 (89.2)	0 (0.0)	4 (2.9)	132 (97.1)	**0.021**
Glaucoma can be controlled by treatment.	4 (3.3)	34 (28.3)	82 (68.3)	8 (5.9)	10 (7.4)	118 (86.8)	**0.000**
Early detection and treatment will not slow the course of glaucoma.	70 (58.3)	28 (23.3)	22 (18.3)	82 (60.3)	15 (11.0)	39 (28.7)	**0.013**
It is possible to completely lose vision as a result of laser treatment or surgery for glaucoma.	24 (20.0)	58 (48.3)	38 (31.7)	34 (25.0)	58 (42.6)	44 (32.4)	0.558

The use of eye drops will be redundant if one has had a laser treatment or surgery for glaucoma.	32 (26.7)	60 (50.0)	28 (23.3)	34 (25.0)	60 (44.1)	32 (30.9)	0.393
Follow-up visit to the clinic is not necessary after the laser treatment or surgery for glaucoma.	81 (67.5)	19 (15.8)	20 (16.7)	91 (66.9)	13 (9.6)	32 (23.5)	0.175
Regular check-ups are not necessary for glaucoma patients.	89 (74.2)	17 (14.2)	14 (11.7)	106 (77.9)	7 (5.1)	23 (16.9)	**0.032**
Even if the intraocular pressure is under control, the visual field has to be checked.	1 (0.8)	34 (28.3)	85 (70.8)	2 (1.5)	18 (13.2)	116 (85.3)	**0.011**
A patient should always tell the ophthalmologist which other medicines (s)he is taking.	2 (1.7)	8 (6.7)	110 (91.7)	0 (0.0)	4 (2.9)	132 (97.1)	0.114
A patient should always tell the ophthalmologist which other diseases (s)he has.	2 (1.7)	9 (7.5)	109 (90.8)	0 (0.0)	3 (2.2)	133 (97.8)	**0.041**
Treatment for glaucoma is lifelong.	23 (19.2)	69 (57.5)	28 (23.3)	13 (9.6)	43 (31.6)	80 (58.8)	**0.000**
Vision loss is reversible after initiating glaucoma treatment.	21 (17.5)	57 (47.5)	42 (35.0)	60 (44.1)	42 (30.9)	34 (25.0)	**0.000**

Most participants were aware of glaucoma symptoms with the exception of convulsive seizures (9.0%). Moreover, only 27.3% were aware that glaucoma can manifest as an asymptomatic entity. Participants with glaucoma were significantly more likely to report vision loss (p = 0.008), and pain, redness, nausea, and vomiting ($X^2(1) = 12.11$, p = 0.001) as symptoms of glaucoma than their ophthalmic non-glaucoma counterparts. Similarly, the treatment of glaucoma with medications is significantly more recognized by participants with glaucoma ($X^2(1) = 8.11$, p = 0.006). The least reported modality of treatment among both groups was surgery (57.5%, and 69.1%).

The least commonly recognized complications of glaucoma treatment were dyspnea (10.9%), bradycardia (12.1%), and elongation of eyelashes (18.0%). In terms of sources of information, participants with glaucoma are significantly more dependent on ophthalmologists for disease-specific information ($X^2(1) = 80.62$, p < 0.001). On the other hand, ophthalmic non-glaucoma participants are significantly more likely to rely on close social circles ($X^2(2) = 15.28$, p < 0.001). **Table 3** describes knowledge about glaucoma's presentation and its treatment.

Table 3: Awareness of risk factors, treatment options, and difficulties associated with glaucoma

		Ophthalmic non-glaucoma patients	Glaucoma patients	p-value*
Signs & symptoms of glaucoma				
	Asymptomatic	29 (24.2)	41 (30.1)	0.326
	Reduced visual acuity	106 (88.3)	123 (90.4)	0.685
	Narrow field of vision	62 (51.7)	85 (62.5)	0.100
	Vision loss	55 (45.8)	85 (62.5)	**0.008**
	Convulsive seizures	11 (9.2)	12 (8.8)	1.000

	Pain, redness, nausea, and vomiting	41 (34.2)	76 (55.9)	**0.001**
Treatment options for glaucoma				
	Medications	75 (62.5)	107 (78.7)	**0.006**
	Surgical procedures	93 (77.5)	118 (86.8)	0.070
	Laser therapy	69 (57.5)	94 (69.1)	0.068
Complications of treatment				
	Stinging and burning	90 (75.0)	96 (70.6)	0.483
	Blurred vision	87 (72.5)	96 (70.6)	0.782
	Discoloration of iris	29 (24.2)	29 (21.3)	0.654
	Dyspnea	10 (8.3)	18 (13.2)	0.234
	Tachypnea	15 (12.5)	16 (11.8)	1.000
	Elongation of lashes	9 (7.5)	37 (27.2)	**0.000**
	Redness, dryness, and itching	64 (53.3)	100 (73.5)	**0.001**
Source of information				
	Ophthalmologists	35 (29.2)	115 (84.6)	**0.000**
	Non-ophthalmologists	10 (8.3)	8 (5.9)	0.472
	Friends and family	53 (44.2)	29 (21.3)	**0.000**
	TV/Newspapers	32 (26.7)	27 (19.9)	0.234
	Internet	45 (37.5)	56 (41.2)	0.609
	Social Media	21 (17.5)	23 (16.9)	1.000
Difficulties due to ophthalmic conditions				
	Identifying persons	27 (22.5)	54 (39.7)	**0.005**
	Reading	39 (32.5)	73 (53.7)	**0.001**
	Using stairs	21 (17.5)	44 (32.4)	**0.009**
	Watching TV	31 (25.8)	54 (39.7)	**0.024**
	Driving	30 (25.0)	52 (38.2)	**0.031**
	Use social media	23 (19.2)	25 (25.7)	0.233
	Walking alone	14 (11.7)	32 (23.5)	**0.015**
	Differentiating colors and clothes	20 (16.7)	28 (20.6)	0.521
	No difficulties	70 (58.3)	43 (31.6)	**0.000**

*Conducted using the chi-squared test

Compared to their ophthalmic non-glaucoma counterparts, those diagnosed with glaucoma face significantly more daily life difficulties due to their ophthalmic disease (p <0.001). Such difficulties include identifying persons (p = 0.005), reading (p = 0.001), using the stairs (p = 0.009), driving (p = 0.031), walking alone (p = 0.015), among others. Overall, most participants request that more information on the pathophysiology/progression (83.6%), treatment (68.4%) of glaucoma, and other practical advice on how to manage disease fluctuations (71.1%) are needed to improve awareness and knowledge of glaucoma.

Univariate analysis shows that participants with glaucoma have significantly higher knowledge scores (t(254) = 4.31, p <0.001) and were able to recognize more glaucoma symptoms than their non-glaucoma counterparts (t(254) = 3.18, p = 0.002). Similarly, those with a positive family history of glaucoma displayed higher knowledge (t(249) = 2.81, p = 0.005) and a relatively higher ability to recognize glaucoma symptoms (t(249) = 2.07, p = 0.039). Knowledge scores did not differ between genders, area of residence, educational status, income level, or medical history (refer to **table 4**). Moreover, the ability to recognize more symptoms and more treatment-related complications was positively correlated with higher knowledge scores (r = 0.302, p <0.001; r = 0.285, p <0.001, respectively). However, age (p = 0.130), number of clinic visits per month (p = 0.410), and number of clinic visits per year (p = 0.412) were not significantly correlated with knowledge scores.

Table 4: Factors affecting glaucoma knowledge scores

		Knowledge Score	p-value	Symptom recognition	p-value	Adverse effect recognition	p-value
Disease Status			**0.000**		**0.002**		0.074
	Ophthalmic non-glaucoma patients	14.6 ± 6.1		2.5 ± 1.4		2.5 ± 1.6	
	Glaucoma patients	17.5 ± 4.1		3.1 ± 1.4		2.9 ± 1.5	
Biological Sex			0.442		**0.028**		0.204
	Male	16.4 ± 5.3		3.0 ± 1.5		2.8 ± 1.7	
	Female	15.8 ± 5.4		2.6 ± 1.4		2.6 ± 1.4	
Residence			0.856		0.929		0.642

	Amman (Capital)	16.1 ± 5.3		2.8 ± 1.4		2.7 ± 1.6	
	Central	15.8 ± 6.1		2.8 ± 1.4		2.7 ± 1.4	
	South	17.1 ± 2.8		2.6 ± 1.3		2.3 ± 1.1	
	North	16.6 ± 4.1		2.6 ± 1.7		2.3 ± 1.1	
Education			0.076		0.440		0.114
	Illiterate	16.3 ± 6.0		3.1 ± 1.4		3.3 ± 1.6	
	Primary	16.2 ± 4.6		3.1 ± 1.3		2.6 ± 1.3	
	Highschool	14.7 ± 6.2		2.7 ± 1.6		2.6 ± 1.6	
	Bachelors	16.8 ± 4.6		2.7 ± 1.4		2.5 ± 1.5	
	Master's +	17.4 ± 5.1		3.0 ± 1.4		3.2 ± 1.7	
Income			0.077		0.460		0.110
	< 499	16.0 ± 5.4		2.9 ± 1.5		2.7 ± 1.5	
	500 – 999	16.4 ± 5.1		2.6 ± 1.4		2.5 ± 1.5	
	1000 – 1999	15.1 ± 5.3		2.9 ± 1.1		2.4 ± 1.2	
	> 2000	22.0 ± 1.0		3.4 ± 0.8		4.2 ± 1.8	
Comorbidities			0.346		0.448		0.938
	No	15.8 ± 5.9		2.9 ± 1.4		2.7 ± 1.6	
	Yes	16.4 ± 4.7		2.7 ± 1.4		2.7 ± 1.5	
Smoking history			0.699		0.754		0.471
	No	16.1 ± 5.3		2.8 ± 1.5		2.7 ± 1.5	
	Yes	16.4 ± 5.6		2.9 ± 1.4		2.8 ± 1.5	
Family history of glaucoma			**0.005**		**0.039**		0.154
	No	15.5 ± 5.8		2.7 ± 1.4		2.6 ± 1.5	
	Yes	17.5 ± 3.9		3.1 ± 1.5		2.9 ± 1.6	

Multivariate linear regression demonstrates that a family history of glaucoma (B: 1.539; 95%CI: 0.078 – 3.000; p-value: 0.039), higher symptom recognition score (B: 0.624; 95%CI: 0.083 – 1.164, p-value: 0.024), relying on ophthalmologists (B: 2.584; 95%CI: 0.983 – 4.185; p-value: 0.002), and the internet (B: 2.364; 95%CI: 0.845 – 3.883: p-value: 0.002) for glaucoma-related information are positive predictors of higher knowledge scores. **Table 5** delineates the linear regression model for knowledge scores.

Table 5: Predictors of knowledge score

	**Linear regression for knowledge score**				
	**p-value**	**B**	**ß**	**Lower 95% CI for (B)**	**Upper 95% CI for (B)**
Age	0.390	0.019	0.067	-0.025	0.064
Female gender	0.334	0.672	0.065	-0.696	2.041
Male gender (**Reference**)	—	—	—	—	—
Family history of Glaucoma	**0.039**	1.539	0.138	0.078	3.000

No family history of Glaucoma (**Reference**)	—	—	—	—	—
Positive presence of comorbidities	0.712	0.290	0.028	-1.259	1.840
Smoking	0.425	0.651	0.052	-0.957	2.260
No smoking (**Reference**)	—	—	—	—	—
Clinic Visit Per Year	0.724	-0.019	-0.025	-0.122	0.085
Symptom recognition score	**0.024**	0.624	0.175	0.083	1.164
Adverse effects recognition score	0.568	0.163	0.048	-0.399	0.724
Number of faced difficulties due to ophthalmic disease	0.445	0.108	0.055	-0.170	0.386
**Sources of information**					
Ophthalmologists	**0.002**	2.584	0.239	0.983	4.185
Non-Ophthalmologists	0.376	1.324	0.060	-1.621	4.268
Friends and Family	0.832	-0.174	-0.015	-1.783	1.436
TV/Newspapers	0.696	-0.344	-0.027	-2.081	1.392
Internet	**0.002**	2.364	0.227	0.845	3.883
Social Media	0.518	-0.631	-0.047	-2.554	1.292

Discussion

Our study demonstrated that glaucoma knowledge among Jordanian ophthalmic patients is rather underwhelming and extremely variable. Knowledge deficiencies are most pronounced in areas such as risk factors, neurological symptoms, adverse effects of over-the-counter medications of glaucoma, and post-operative management and expectations. Additionally, it appears that the extent of knowledge of recruited participants is only influenced by their glaucoma diagnosis and family history of glaucoma as all sociodemographic factors did not significantly impact overall knowledge.

The level of knowledge of glaucoma portrays significant variability across different reports throughout the globe. Local Jordanian reports show that while Jordanian patients visiting

Jordan University Hospital were mostly aware of glaucoma, only 34.1% were able to define it accurately [26]. In contrast, only 38.8% of the Jordanian population was aware of glaucoma [18]. In terms of regional literature, a Saudi Arabian report demonstrates that patients with glaucoma exhibit poor knowledge, attitudes, and practices toward their disease [27]. Similarly, a survey of Iraqi ophthalmic patients with disease-induced blindness demonstrated that lack of awareness is the most dominant factor affecting patients with glaucoma [28]. An Iranian population-based survey demonstrated that only 46.6% demonstrated glaucoma awareness while only 19.2% were able to correctly define the disease [29]. Moreover, among Egyptian adolescents with glaucoma, satisfactory knowledge was as low as 12% prior to the initiation of an educational program.[30] Asian countries, the likes of Nepal and India, demonstrated low rates of glaucoma knowledge among hospital presenting patients ranging from 1.9% to 27% [31–33]. China, on the other hand, demonstrated that 51.6% of glaucoma patients visiting a tertiary university hospital were aware of their disease [34].

Great variance is noted among African nations in terms of glaucoma awareness as rates range from 74% among Ghanian patients with glaucoma visiting a tertiary teaching hospital, 36.8% among Nigerians with glaucoma, 46.7% among Ethiopian glaucoma patients, to 2.4% among Ethiopian patients visiting the ophthalmic outpatient clinics [35–38]. On the other hand, Western nations such as Austria, Germany, the United States, Australia, and the United Kingdom display far superior rates of glaucoma knowledge ranging from 51% to 93% among the public and patients visiting eye clinics [19,39–41]. Nonetheless, marked variability does exist among such a report in which an investigation of Swiss patients with glaucoma demonstrated low levels of glaucoma knowledge as only 28% were able to define their disease [5].

While such variance among different studies could be attributed to differences between studied populations in terms of sociodemographic or clinical characteristics, inherent

methodological differences make a direct comparison between any two sets of reports unfeasible. There seems to be an overlap, or a rather failure of distinction, between knowledge and awareness [22]. Across the aforementioned studies, measures of knowledge assessment were dramatically different in terms of content, format (e.g., number and type of questions), and definitions of proper knowledge, most of which were made based on arbitrary and/or convenient basis [42]. Furthermore, those questionnaire-based measures were not always developed rigorously as development discrepancies (e.g., presence or absence of pilot testing, expert group consultation, and extensive literature review of similar studies) could lead to the heterogenous observation of glaucoma-targeted knowledge.

We demonstrated that glaucoma patients are more knowledgeable about glaucoma in comparison to ophthalmic non-glaucoma counterparts, yet the knowledge among the two groups was relatively poor. Such observation is fairly established within the literature and is expected as those with established glaucoma diagnosis receive a greater fund of disease-specific knowledge from their doctors or close social circles at far higher frequencies of contact [23]. Literature shows that durations of glaucoma and follow-up are significantly associated with improved levels of knowledge [12,43]. Another plausible explanation is the reliance of ophthalmic non-glaucoma patients on friends and family for glaucoma-related information, as well as lack of clinical visits, both of which may predispose them to inaccurate or outdated information. Such reliance is rather interesting as all recruited participants were from a hospital setting which may indicate a gap of trust between patients and healthcare providers within our tertiary hospital. Reliance on "friends and family" among the public or ophthalmic non-glaucoma patients has been reported in a German survey, which demonstrated fairly poor levels of glaucoma knowledge [41]. Hassan et al., (2021) mentioned that the large number of patients seen by doctors in primary healthcare clinics as well as in ophthalmology clinics in Jordan results in less doctor-patient time in the clinic which

consequently makes doctor-patient encounters less informative [26]. Increasing the number of serving physicians in ophthalmology clinics and fortifying their communication skills may ensure the appropriate delivery of vital glaucoma-related information to patients, however, this solution might be over-ambitious in resource-scarce institutions and developing nations.

Amongst the literature, several factors were implicated to have influenced glaucoma-related knowledge such as age, education, occupation, income, accessibility of ophthalmic care, duration of glaucoma, compliance to medications, family history of glaucoma, and type of glaucoma [20,22,34]. Chen et al., (2022) demonstrate a compressive review of such factors and show that lack of conformity between the impact of different factors on glaucoma knowledge can be explained by differences in study design, characteristics of study populations, and methods of analysis [34]. In agreement with our investigation, several studies reported that participants with a family history of glaucoma were significantly more knowledgeable about their condition [12,26,41]. Such might be the case because having a family member with glaucoma may provoke the individual to search for more information and/or gather information during their appointment with the eye care professional [31]. Nonetheless, this association is not always consistent across the literature as family history of glaucoma failed to improve levels of glaucoma knowledge among Swiss glaucoma patients visiting tertiary glaucoma clinics [5]. Contrary to the literary consensus that shows higher educational standards associated with more glaucoma knowledge [21,25,34], educational level was not significantly associated with glaucoma knowledge among both groups of studied participants. This can be attributed to weakness in the Jordanian health-related educational programs for secondary and higher levels of education [26]. Additionally, age did not significantly predict knowledge scores among included participants. Interestingly, age was predominantly a negative predictor of glaucoma knowledge within the literature. A variety of factors could've influenced such an association such as the low levels of health

literacy in older individuals, neurological disease progression, or the inherent technological bias exhibited by younger participants able to fully utilize the internet to gain disease-related knowledge [34,44].

Since glaucoma is a major contributor to vision loss in Jordan and worldwide, lack of knowledge about glaucoma makes improving information about this disease a necessity for Jordanian and global communities. Achieving acceptable long-term visual outcomes in glaucoma relies on controlling the eye pressure which is dependent on the patient adhering to the treatment regimen [45]; a dimension that can be improved by improving patient knowledge about glaucoma [46,47]. This can be achieved through using a patient-held record (e.g., 'Glaucoma Logbook'), which is an A5-sized book containing some basic and important information about glaucoma, patient information, details of the treating medical team, intraocular pressure, medications, and details of support services. Such a method proved to be effective in increasing patients' knowledge of glaucoma among those treated for glaucoma or ocular hypertension [48]. Tackling the issue of poor patient knowledge is of great importance as both studied groups were able to identify less than 50% of the proposed adverse effects associated with glaucoma medications. Considering the wide spectrum of systemic side effects associated with beta-blockers and alpha-agonists, targeted solutions should focus on such deficiency [12].

Institution-wise, a multitude of research-proven interventions could be implemented to tackle these gaps of knowledge. Chen et al., (2009) demonstrated the impact of creating a glaucoma club on recruited participants [49]. Participants within the club had significantly higher levels of glaucoma knowledge, improved disease cognition, experienced improved compliance to medications, and lived healthier lives. Li et al., (2018) showed that a mobile-based education program, administered through social media (e.g., WeChat), was effective in improving patients' education towards glaucoma and enhanced their willingness to utilize electronic

services for their follow-up [23]. The authors argue that such programs, not only enhance public awareness, but also improve treatment adherence and clinical outcomes. Tatham et al., (2019) demonstrated that virtual clinic visits yielded the same, yet defective, levels of glaucoma knowledge compared to those attending face-to-face clinics [50]. This may suggest the adoption and enhancement of a telemedicine program centered around patient education may impact knowledge with lesser long-term costs. Finally, the formulation of a patient-centered educational program was proven effective in enhancing the knowledge of patients with congenital and secondary glaucoma [30]. Such a program may take different forms ranging from seminars, written publications (e.g., brochures and leaflets), and video materials, to theory-based practical sessions given throughout a patient's clinical visit. Peralta et al., (2018) stressed on the importance of such programs to increase knowledge and raise awareness about glaucoma [42].

The burden of ameliorating glaucoma awareness and knowledge should also be shared with governing bodies and health policymakers. Our multivariate analysis demonstrated that ophthalmologists and the internet are associated with increased levels of knowledge; an observation that was documented in China [34]. Concerned governmental authorities and health policymakers should increase the public's awareness and knowledge by allocating more resources towards enhancing ophthalmology practice and utilizing internet-based solutions to reach a wide audience. Improving awareness was shown to help in early glaucoma detection, thus better control and recognition of its complications [25]. Moreover, early detection, control, and treatment will mitigate healthcare costs of patients over the long-term and alleviate the medical burden by encouraging patients to seek medical treatment [24].

This study is not devoid of limitations. The cross-sectional nature of its design is already associated with a handful of biases including sampling bias that may result in a snapshot of the population that may not reflect the original sampling pool. The small sample size might

have impacted the statistical robustness by which knowledge scores are differentiated. Moreover, the study examines glaucoma knowledge in a different, yet more comprehensive, manner than the available literature thus it might underestimate its rates of glaucoma awareness. Moreover, data may be subjected to recall bias due to the self-reporting nature of the questionnaire. Finally, we did not investigate compliance with medications which could be affected by glaucoma-related knowledge. Nonetheless, the strength of the study lies in its usage of a rigorously designed and validated questionnaire to evaluate glaucoma-related knowledge. Also, the study's sample of glaucoma patients closely resembles the overall number of glaucoma patients treated at the JUH ophthalmology clinics thus a high internal validity.

Conclusion

In light of this investigation, we have demonstrated that ophthalmic patients display mediocre levels of glaucoma knowledge. Raising awareness through various programs and interventions may improve the lifestyles of patients with glaucoma, which may result in long-term mitigation of the economic burden associated with tending to the chronic and severe complications of such disease. Concerned authorities should project their interventions through ophthalmologists and internet-based platforms due to their profound effect in shaping glaucoma-related knowledge.

Acknowledgments

None

Declaration of interest

The authors have declared that no competing interests exist.

Funding disclosure statement

The author(s) received no specific funding for this work

Author contributions

Conceptualization: Sana’ Muhsen, Abdallah Al-Ani

Data Curation: Leen Al-Huneidy

Formal analysis: Abdallah Al-Ani, Leen Al-Huneidy

Methodology: Abdallah Al-Ani, Lina AlQirem, Leen Al-Huneidy, Ward Maaita

Supervision: Sana’ Muhsen

Writing – original draft: All authors

Writing – review & editing: Sana’ Muhsen, Abdallah Al-Ani, Leen Al-Huneidy

Supporting information

S1 Data. Data from the administered survey

S2 File. Data collection instrument (questionnaire)

References

1. Weinreb RN, Aung T, Medeiros FA. The pathophysiology and treatment of glaucoma: A review. JAMA - J Am Med Assoc. 2014;311: 1901–1911. doi:10.1001/jama.2014.3192

2. Uche NJ, Udeh NN, Chuka-Okosa CM, Kizor-Akaraiwe NN, Uche EO. Glaucoma care and follow-up in sub-Saharan Africa: Is there a need for modification of counselling practices to improve awareness, knowledge and treatment acceptance profiles? A

prospective cross-sectional study. Int Ophthalmol. 2020;40: 1539–1546. doi:10.1007/s10792-020-01323-6

3. Tham YC, Li X, Wong TY, Quigley HA, Aung T, Cheng CY. Global prevalence of glaucoma and projections of glaucoma burden through 2040: A systematic review and meta-analysis. Ophthalmology. 2014;121: 2081–2090. doi:10.1016/j.ophtha.2014.05.013

4. Sood D, Sood S, Sood I, Kumar D, Sood R, Sood NN. Glaucoma awareness amongst glaucoma patients seeking a second opinion. Int Ophthalmol. 2018;38: 1441–1449. doi:10.1007/s10792-017-0604-5

5. Mansouri K, Iliev ME, Rohrer K, Shaarawy T. Compliance and knowledge about glaucoma in patients at tertiary glaucoma units. Int Ophthalmol. 2011;31: 369–376. doi:10.1007/s10792-011-9468-2

6. Noertjojo K, Maberley D, Bassett K, Courtright P. Awareness of eye diseases and risk factors: Identifying needs for health education and promotion in Canada. Can J Ophthalmol. 2006;41: 617–623. doi:10.1016/S0008-4182(06)80035-9

7. Dandona R. Awareness of eye diseases in an urban population in southern India. Bull World Health Organ. 2001;79: 96–102.

8. Fabjani S, Richter-Müksch S, Cakmak N, Markstaller M, Weingessel B, Vécsei-Marlovits VP. Informationsstand über die Erkrankung Glaukom in Wien. Wien Klin Wochenschr. 2015;127: 612–618. doi:10.1007/s00508-014-0687-1

9. Hoevenaars JGMM, Schouten JSAG, Van Den Borne B, Beckers HJM, Webers CAB. Knowledge base and preferred methods of obtaining knowledge of glaucoma patients. Eur J Ophthalmol. 2005;15: 32–40. doi:10.1177/112067210501500106

10. Hoevenaars JGMM, Schouten JSAG, van den Borne B, Beckers HJM, Webers CAB. Socioeconomic differences in glaucoma patients' knowledge, need for information and expectations of treatments. Acta Ophthalmol Scand. 2006;84: 84–91. doi:10.1111/j.1600-0420.2005.00587.x

11. Li J, Huang W, Gao J, Li D, Xu L, Huang J. Impact of Mobile-Based Health Education on the Awareness and Knowledge of Glaucoma in Chinese Patients. Telemed E-Health. 2019;25: 455–461. doi:10.1089/tmj.2018.0123

12. Danesh-Meyer H V., Deva NC, Slight C, Tan YW, Tarr K, Carroll SC, et al. What do people with glaucoma know about their condition? A comparative cross-sectional incidence and prevalence survey. Clin Experiment Ophthalmol. 2008;36: 13–18. doi:10.1111/j.1442-9071.2007.01624.x

13. Adegbehingbe BO, Bisiriyu LA. Knowledge, attitudes, and self care practices associated with glaucoma among hospital workers in Ile-Ife, Osun State, Nigeria. Tanzan J Health Res. 2008;10: 240–245. doi:10.4314/thrb.v10i4.45080

14. Saw SM, Gazzard G, Friedman D, Foster PJ, Devereux JG, Wong ML, et al. Awareness of glaucoma, and health beliefs of patients suffering primary acute angle closure. Br J Ophthalmol. 2003;87: 446–449. doi:10.1136/bjo.87.4.446

15. Gatwood J, Brooks C, Meacham R, Abou-Rahma J, Cernasev A, Brown E, et al. Facilitators and Barriers to Glaucoma Medication Adherence. J Glaucoma. 2022;31: 31–36. doi:10.1097/IJG.0000000000001965

16. Trevethan R. Deconstructing and Assessing Knowledge and Awareness in Public Health Research. Front Public Health. 2017;5: 194. doi:10.3389/fpubh.2017.00194

17. Rabiu MM, Al Bdour MD, Abu Ameerh MA, Jadoon MZ. Prevalence of blindness and diabetic retinopathy in northern Jordan. Eur J Ophthalmol. 2015;25: 320–327. doi:10.5301/ejo.5000557

18. Haddad MF, Bakkar MM, Abdo N. Public awareness of common eye diseases in Jordan. BMC Ophthalmol. 2017;17: 177. doi:10.1186/s12886-017-0575-3

19. Fabjani S, Richter-Müksch S, Cakmak N, Markstaller M, Weingessel B, Vécsei-Marlovits VP. Informationsstand über die Erkrankung Glaukom in Wien. Wien Klin Wochenschr. 2015;127: 612–618. doi:10.1007/s00508-014-0687-1

20. Hoevenaars JGMM, Schouten JSAG, van den Borne B, Beckers HJM, Webers CAB. Socioeconomic differences in glaucoma patients' knowledge, need for information and expectations of treatments. Acta Ophthalmol Scand. 2006;84: 84–91. doi:10.1111/j.1600-0420.2005.00587.x

21. Hoevenaars JGMM, Schouten JSAG, Van Den Borne B, Beckers HJM, Webers CAB. Knowledge base and preferred methods of obtaining knowledge of glaucoma patients. Eur J Ophthalmol. 2005;15: 32–40. doi:10.1177/112067210501500106

22. Uche NJ, Udeh NN, Chuka-Okosa CM, Kizor-Akaraiwe NN, Uche EO. Glaucoma care and follow-up in sub-Saharan Africa: Is there a need for modification of counselling practices to improve awareness, knowledge and treatment acceptance profiles? A prospective cross-sectional study. Int Ophthalmol. 2020;40: 1539–1546. doi:10.1007/s10792-020-01323-6

23. Li J, Huang W, Gao J, Li D, Xu L, Huang J. Impact of Mobile-Based Health Education on the Awareness and Knowledge of Glaucoma in Chinese Patients. Telemed E-Health. 2019;25: 455–461. doi:10.1089/tmj.2018.0123

24. Saw SM, Gazzard G, Friedman D, Foster PJ, Devereux JG, Wong ML, et al. Awareness of glaucoma, and health beliefs of patients suffering primary acute angle closure. Br J Ophthalmol. 2003;87: 446–449. doi:10.1136/bjo.87.4.446

25. Sood D, Sood S, Sood I, Kumar D, Sood R, Sood NN. Glaucoma awareness amongst glaucoma patients seeking a second opinion. Int Ophthalmol. 2018;38: 1441–1449. doi:10.1007/s10792-017-0604-5

26. Hassan DWA, Alsibai BA, Alhouri AN, Alhajeh RY, Suleiman AA, Al Bdour MD. Awareness and knowledge about glaucoma among patients attending outpatient clinics of Jordan University Hospital. 2021. doi:10.4103/1319-4534.322618

27. Al Rashed WA, Bin Shihah AS, Alhomoud AS, Alharbi MM, Shadid AM, Alghamdi MA, et al. Knowledge, attitude, and practice toward glaucoma and its management

among adult Saudi patients. Saudi J Ophthalmol Off J Saudi Ophthalmol Soc. 2020;34: 261–265. doi:10.4103/1319-4534.322596

28. Al-Shakarchi FI. Blindness in iraq: leading causes, target patients, and barriers to treatment. Middle East Afr J Ophthalmol. 2011;18: 199–203. doi:10.4103/0974-9233.84044

29. Katibeh M, Ziaei H, Panah E, Moein H-R, Hosseini S, Kalantarion M, et al. Knowledge and Awareness of Age Related Eye Diseases: a Population-Based Survey. J Ophthalmic Vis Res. 2014;9: 223–231.

30. Mohamed EA, Bayoumi OR, Draz SF. Impact of an educational programme on knowledge, beliefs, practices and expectations about care among adolescent glaucoma patients in Cairo. East Mediterr Health J Rev Sante Mediterr Orient Al-Majallah Al-Sihhiyah Li-Sharq Al-Mutawassit. 2011;17: 960–968. doi:10.26719/2011.17.12.960

31. Gyawali R, Sarkar N. Glaucoma awareness in a hospital presenting population in eastern Nepal. J Glaucoma. 2014;23: 594–598. doi:10.1097/IJG.0b013e31828700de

32. Rewri P, Kakkar M. Awareness, knowledge, and practice: A survey of glaucoma in north Indian rural residents. Indian J Ophthalmol. 2014;62: 482–486. doi:10.4103/0301-4738.132105

33. Maharana PK, Rai VG, Pattebahadur R, Singhi S, Chauhan AK. Awareness and Knowledge of Glaucoma in Central India: A Hospital-Based Study. Asia-Pac J Ophthalmol Phila Pa. 2017;6: 243–249. doi:10.22608/APO.2015166

34. Chen X, Zhong Y-L, Chen Q, Tao Y-J, Yang W-Y, Niu Z-Q, et al. Knowledge of glaucoma and associated factors among primary glaucoma patients in Kunming, China. BMC Ophthalmol. 2022;22: 95. doi:10.1186/s12886-022-02322-0

35. Kizor-Akaraiwe NN, Monye HI, Okeke S. Awareness and knowledge about glaucoma and proportion of people with glaucoma in an urban outreach programme in Southeast Nigeria. BMJ Open Ophthalmol. 2017;1: 1–10. doi:10.1136/bmjophth-2016-000018

36. Tenkir A, Solomon B, Deribew A. Glaucoma awareness among people attending ophthalmic outreach services in Southwestern Ethiopia. BMC Ophthalmol. 2010;10. doi:10.1186/1471-2415-10-17

37. Alemu DS, Gudeta AD, Gebreselassie KL. Awareness and knowledge of glaucoma and associated factors among adults: a cross sectional study in Gondar Town, Northwest Ethiopia. BMC Ophthalmol. 2017;17: 154. doi:10.1186/s12886-017-0542-z

38. Nkum G, Lartey S, Frimpong C, Micah F, Nkum B. Awareness and Knowledge of Glaucoma Among Adult Patients at the Eye Clinic of a Teaching Hospital. Ghana Med J. 2015;49: 195–199. doi:10.4314/gmj.v49i3.11

39. Gasch AT, Wang P, Pasquale LR. Determinants of glaucoma awareness in a general eye clinic. Ophthalmology. 2000;107: 303–308. doi:10.1016/S0161-6420(99)00076-7

40. Baker H, Cousens SN, Murdoch IE. Poor public health knowledge about glaucoma: Fact or fiction. Eye. 2010;24: 653–657. doi:10.1038/eye.2009.155

41. Pfeiffer N, Krieglstein GK, Wellek S. Knowledge about glaucoma in the unselected population: A German survey. J Glaucoma. 2002;11: 458–463. doi:10.1097/00061198-200210000-00016

42. Peralta E, Muir KW, Rosdahl JA. Systematic Review of Knowledge Assessments for Glaucoma Patients. Semin Ophthalmol. 2018;33: 377–388. doi:10.1080/08820538.2016.1247180

43. Gray TA, Fenerty C, Harper R, Lee A, Spencer AF, Campbell M, et al. Preliminary survey of educational support for patients prescribed ocular hypotensive therapy. Eye. 2010;24: 1777–1786. doi:10.1038/eye.2010.121

44. Schönfeld MS, Pfisterer-Heise S, Bergelt C. Self-reported health literacy and medication adherence in older adults: a systematic review. BMJ Open. 2021;11: e056307. doi:10.1136/bmjopen-2021-056307

45. Meyers MA. Distribution of intra-abdominal malignant seeding: dependency on dynamics of flow of ascitic fluid. Am J Roentgenol Radium Ther Nucl Med. 1973;119: 198–206. doi:10.2214/ajr.119.1.198

46. Kashiwagi K, Tsukahara S. Impact of patient access to internet health records on glaucoma medication: Randomized controlled trial. J Med Internet Res. 2014;16: 1–16. doi:10.2196/jmir.2795

47. Attebo K, Mitchell P, Cumming R, Smith W. Knowledge and beliefs about common eye diseases. Aust N Z J Ophthalmol. 1997;25: 283–287. doi:10.1111/j.1442-9071.1997.tb01516.x

48. Daly R, Agarwal PK. Is Patient Knowledge of Glaucoma Improved by Using a Glaucoma Logbook? J Glaucoma. 2018;27: 470–475. doi:10.1097/IJG.0000000000000943

49. Chen X, Chen Y, Sun X. Notable role of glaucoma club on patients' knowledge of glaucoma. Clin Experiment Ophthalmol. 2009;37: 590–594. doi:10.1111/j.1442-9071.2009.02101.x

50. Tatham AJ, Ali AM, Hillier N. Knowledge of Glaucoma Among Patients Attending Virtual and Face-to-Face Glaucoma Clinics. J Glaucoma. 2021;30: 325–331. doi:10.1097/IJG.0000000000001758

Click here to access/download
Supporting Information
S2_File.pdf

Click here to access/download
Supporting Information
S1_Data.xlsx

Revised Manuscript with Track Changes

Predictors of glaucoma knowledge and its risk factors among Jordanian patients with primary open angle glaucoma at a tertiary teaching hospital: a cross-sectional survey

Authors:

Sana' Muhsen[1*], Leen Al-Huneidy[2], Ward Maaita[2], Lina AlQirem[2], Zaid Madain[2], Jaleel Sweis[2], Raya Abu Tawileh[2], Yazan Al-Huneidy[2], Amro Alkhatib[2], Abdallah Al-Ani[2,3]

Affiliations:

[1]Special Surgery Department/Ophthalmology Division, School of Medicine, University of Jordan, Amman, Jordan

[2]School of Medicine, University of Jordan, Amman, Jordan

[3]Office of Scientific Affairs and Research, King Hussein Cancer Center, Amman, Jordan

* **Corresponding author**:

E-mail: sana_mohsen79@yahoo.com

Funding: This work has received no funding

Abstract

Purpose of study: To assess the glaucoma knowledge and adherence to care seeking behaviors among Jordanian patients with glaucoma to formulate policies and compliance assuring strategies.

Methods: A cross-sectional survey was developed after an extensive literature search to investigate glaucoma-related knowledge among participants with glaucoma visiting the Jordan University Hospital clinics from October 2021 to February 2022. Responses were compared to a sample of ophthalmic participants with eye conditions other than glaucoma visiting the ophthalmology clinics at the same time frame.

Results: A total of 256 participants filled out the survey, of which 53.1% were diagnosed with glaucoma while 46.9% had ophthalmic conditions other than glaucoma. Our sample of participants is characterized by a mean age of 52.2 ± 17.8 years and a ~~male to female~~male-to-female ratio of 1.04:1. Overall, participants with glaucoma were more aware of their disease than participants with other ophthalmic conditions. Compared to their ophthalmic non-glaucoma counterparts, those diagnosed with glaucoma face significantly more daily life difficulties due to their ophthalmic disease (p <0.001). ~~Analysis~~ Results of the independent sample *t*-test demonstrate ~~shows~~ that participants with glaucoma have significantly higher knowledge scores (p <0.001) and were able to recognize more glaucoma symptoms than their non-glaucoma counterparts (p = 0.002). Similarly, those with a positive family history of glaucoma displayed higher knowledge (p = 0.005). ~~Multivariate analysis~~Multivariate linear regression demonstrates that family history of glaucoma, higher symptom recognition score, ~~relying~~ reliance on ophthalmologists, and the internet for glaucoma-related information are positive predictors of higher knowledge scores.

Conclusion: We have demonstrated that ophthalmic patients display mediocre levels of glaucoma knowledge. Raising awareness through various interventions may improve the

Formatted: Font: Italic

lifestyles of patients with glaucoma and alleviate the economic burden associated with treating the disease.

Keywords: Glaucoma; Jordan; Awareness; Compliance; Education

Introduction

Glaucoma encompasses a spectrum of diseases characterized by degeneration of the retinal ganglion cells leading to optic disc damage and irreversible vision loss [1]. Being the second most common cause of blindness worldwide, glaucoma affected 64.3 million adults in 2013 and is expected to affect 111.8 million by 2040 [2,3]. Vision loss associated with glaucoma can be prevented with early diagnosis and treatment; however, due to its asymptomatic nature, lack of awareness of risk, and low levels of referral, the incidence of glaucoma is rising within the population [4]. In addition, the chronicity of the disease implies that patients with glaucoma may be subjected to various quality of life and therapeutic concerns due to long-term noncompliance, gender discrimination, and health literacy, all of which are influenced by the patients' awareness towards their disease [4,5].

Despite the discrepancies between developed and developing nations in terms of knowledge ~~in~~ of patients with glaucoma [6,7], the overall level of knowledge is poor [8,9]. Throughout the literature, factors such as socioeconomic status, short disease duration, and advanced age were negative predictors of glaucoma knowledge [8,10–12]. Poor glaucoma knowledge influences the clinical progression of the disease as it contributes to a lack of adherence to palliative drug regime~~n~~s, and the underestimation of regular ophthalmic examinations [2,5,13]. Subsequently, those poor clinical presentations are associated with worse treatment outcomes as cases might be treated with more complex surgical options to halt the rapidly progressive and irreversible retinal damage ~~due to the reliance on more complex surgical options, and the progressive and irreversible retinal damage~~ [5,14]. Therefore, knowledge of glaucoma could be of vital importance as a target of interventions aiming to improve the lifestyles among those with glaucoma, particularly high-risk groups (e.g., geriatrics).

Gatwood et al., (2022) demonstrate that disease-related educational interventions improve adherence to glaucoma treatment [15].

The literature evaluating glaucoma awareness knowledge within the Middle East and therefore its treatment implications, is scarce. In fact, an accurate estimation of knowledge is tricky to calculate due to the juxtaposition with the concept of awareness. It should be noted that awareness refers to the generalized or diffuse knowledge of a particular concept (e.g., have you heard of glaucoma?), while knowledge refers to any information that is factual in nature (e.g., prevalence, etiology, risk factors, etc.)[16].

Among Jordanians, glaucoma is the third leading cause of severe bilateral blindness [17]. It is well established that glaucoma-induced blindness is influenced by poor patient compliance [25]; a factor that is associated with the patients' knowledge and comprehension of their disease as delineated in multiple reports [5]. Moreover, an incomplete understanding of glaucoma disease dynamics may exacerbate psychological burdens among patients or leave them oblivious to the blinding nature of glaucoma [12]. Therefore, knowledge of glaucoma could be of vital importance as a target of interventions aiming to improve the lifestyles among those with glaucoma, particularly high-risk groups (e.g., geriatrics). Gatwood et al., (2022) demonstrates that disease-related educational interventions improve adherence to glaucoma treatment [26].

Glaucoma awareness throughout the population was estimated toAmong the Jordanian public, glaucoma awareness is estimated at be approximately 38.8% [18]. NonethelessHowever, there exists no reports evaluating glaucoma knowledge among this populaceJordanians. [16]Thus, this study aims to assess the glaucoma knowledge and adherence to care seeking behaviors among Jordanian patients with glaucoma in order to to formulate relevant educational policies and compliance assuring strategies.

Material and methods

We conducted this cross-sectional study using a self-administered ~~anonymous~~ questionnaire on patients visiting the ophthalmology clinics at Jordan University Hospital from October 2021 to February 2022. The Jordan University Hospital is the largest tertiary and academic health provider in Jordan. In addition, it is the largest referral center for all of central Jordan serving over four million patients.

Patients were enrolled into two groups: The first group consisted of participants with a clinically proven diagnosis of primary open angle glaucoma of at least 6 months ~~who~~ and are on medications. Diagnosis of glaucoma was established on Optical Coherence Tomography. Patients with secondary glaucoma (e.g., pseudoexfoliation syndrome, pigment dispersion syndrome, neovascular glaucoma, inflammatory glaucoma, and medication-induced glaucoma) were excluded. ~~, and~~ The second group of participants consisted of those who ~~are~~ were diagnosed with any vision hindering ophthalmic conditions with the exception of long-standing glaucoma. Such conditions include cataracts, diabetic retinopathy, macular degeneration, and optic neuritis among others. Participants that were younger than 18 years of age, suffer from mental/psychiatric illnesses, and are unable to consent to participation in the survey were excluded from the study. Moreover, participants that failed to complete at least 80% of the questionnaire were also excluded. Systemic random sampling was adopted to recruit every 4th patient from the ophthalmology clinics.

Formatted: Not Highlight

Instrument development

The study's questionnaire was developed based on an extensive literature review of the most relevant articles reporting on the perceived knowledge of glaucoma by both patients with or without glaucoma [12,19–25]. The questionnaire is composed of the following sections:

demographics (5 items), medical history (10 items), perceived glaucoma knowledge (31 items), and a miscellaneous section (4 items). The first 27 statements of the glaucoma knowledge section were presented as Likert-scales with 1 being strongly disagree and 5 being strongly agree. Statement 28 (signs and symptoms of glaucoma), statement 29 (treatment options for glaucoma), and statement 30 (complications of glaucoma treatment) were presented as non-mutually exclusive multiple choices. Miscellaneous items included statements exploring sources of glaucoma-related information, daily life difficulties due to ophthalmic conditions, ways on how to improve glaucoma awareness, and impact of surgical treatment on patients undergoing glaucoma surgery.

The questionnaire items were extracted from the literature, -therefore, -preserving theirits external validity. Furthermore, a panel of two expert ophthalmologists and four senior residents have approved the content of the questionnaire and slightly edited some of its statements to fit ophthalmic practice at the Jordan University Hospital. A pilot testing on 15 patients with glaucoma and 15 patients with ophthalmic disorders other than glaucoma yielded a Cronbach alpha of 0.704 for the 27 knowledge Likert-scales.

The questionnaire underwent back-to-back translation as it was translated from English to Arabic by an expert ophthalmologist and then back to English by an official translator in order to ensure the validity of the initial translation.

Statistical analysis

All statistical analyses and data cleaning were conducted on SPSS version 24 (SPSS Inc., Chicago, IL, USA). Categorical data were reported as frequencies (n (%)), while continuous data were presented as means ± standard deviations. Mean differences among different categories were examined by the independent sample *t*-test (for categorical variables of two

~~groups),~~ or ANOVA. ~~(for categorical variables with more than two groups).~~ Statistical associations between categorical variables were assessed using the chi-square test. Upon conducting chi-square testing, the 5-point Likert-scales were condensed into three categories (strongly disagree and disagree as one category; neutral as one category; strongly agree and agree as one category). Knowledge scores were computed by adding how many correct answers that participants had out of the first 27 knowledge items (1 point per each correct statement resulting in a total score of 27). Symptoms and adverse effects recognition scores were computed as the sum of participants' chosen categories. Pearson R correlation testing was conducted to assess the correlation between continuous variables and knowledge scores. Moreover, a multivariate linear regression model was computed to assess predictors of knowledge score. All statistical tests are conducted with a 95% confidence interval and a 5% error margin. A p-value of less than 0.05 is considered statistically significant.

Ethical considerations

The study's protocol was approved by the Jordan University Hospital Institutional Review Board (ID:212/2022/09) and was issued on the 1st of September 2021 by the aforementioned body. All participants have read and signed an informed written consent form before continuing to complete the questionnaire. The consent form included the participants' right to anonymity, confidentially of their data, right to leave the study, and reassurance that their participation is completely voluntary, is not associated with any kind of short-term benefit or rewards, ~~-~~and does not affect the quality of their received care.

Results

A total of 256 participants filled out the survey, of which 53.1% were diagnosed with glaucoma while 46.9% had ophthalmic conditions other than glaucoma. Our sample of

participants is characterized by a mean age of 52.2 ± 17.8 years and a male to female ratio of 1.04:1. The majority of the sample resided in the capital city of Amman (66.8%), had a bachelor's degree or higher (50.8%), and earned less than 500 JDs in monthly income (64.5%). Characteristics of participants with glaucoma were similar to those without glaucoma with the exception of age (p = 0.009), clinic visits per month (p <0.001), clinic visits per year (p <0.001), and family history of glaucoma (p = 0.006). **Table 1** describes the sociodemographic characteristics of the recruited cohort.

Table 1: Sociodemographic characteristics of the studied population

		Ophthalmic non-glaucoma patients (n = 120)	Glaucoma patients (n = 136)	p-value
Age in years	Mean ± SD (Median)	48.1 ± 16.4 (49.5)	54.6 ± 18.2 (57.0)	**0.009**
Clinic visits per month	Mean ± SD (Median)	0.5 ± 0.8 (0.0)	0.9 ± 0.7 (1.0)	**<0.001**
Clinic visits per year	Mean ± SD (Median)	3.6 ± 5.6 (2.0)	7.0 ± 6.8 (4.0)	**<0.001**
Biological Sex				0.133
	Male	55 (45.8)	76 (55.9)	
	Female	65 (54.2)	60 (44.1)	
Residence				0.832
	Amman (Capital)	80 (66.7)	91 (66.9)	
	Central	29 (24.2)	30 (22.1)	
	South	5 (4.2)	9 (6.6)	
	North	6 (5.0)	6 (4.4)	
Education				0.415
	Illiterate	8 (6.7)	12 (8.8)	
	Primary	10 (8.3)	21 (15.4)	
	Highschool	37 (30.8)	38 (27.9)	
	Bachelors	52 (43.3)	54 (39.7)	
	Master's +	13 (10.8)	11 (8.1)	
Income				0.091
	< 499	69 (57.5)	96 (70.6)	
	500 – 999	39 (32.5)	30 (22.1)	
	1000 – 1999	8 (6.7)	9 (6.6)	
	> 2000	4 (3.3)	1 (0.7)	
Comorbidities				0.133
	No	63 (52.5)	58 (42.6)	
	Yes	57 (47.5)	78 (57.4)	

Smoking history				0.373
	No	89 (74.2)	108 (79.4)	
	Yes	31 (25.8)	28 (20.6)	
Family history of glaucoma				**0.006**
	No	90 (77.6)	83 (61.5)	
	Yes	26 (21.7)	52 (38.5)	

Amongst participants with glaucoma, the mean time since glaucoma diagnosis was 9.7 ± 9.4 years, and the mean time since the start of glaucoma treating drops was 9.1 ± 8.7 years. Participants with glaucoma used a mean of 2.4 ± 1.4 medications and a mean of 3.3 ± 2.9 drops per day for the treatment and/or control of their glaucoma. Furthermore, participants were subjected to a mean of 1.6 ± 2.7 surgeries for the treatment of glaucoma in their lifetime.

Table 2 demonstrates the responses of~~by~~ participants towards glaucoma knowledge items. Overall, participants with glaucoma were more aware of their disease than participants with other ophthalmic conditions. Participants with glaucoma were significantly more likely than their non-glaucoma counterparts to recognize that glaucoma is a predominantly ophthalmic disease ($X^2(2) = 11.16$, p = 0.004), is the result of increased intraocular pressure ($X^2(2) = 19.56$, p <0.001), can progress to optic nerve damage ($X^2(2) = 29.41$, p <0.001), and is more likely to increase in incidence with age ($X^2(2) = 9.13$, p = 0.010). Moreover, they were more likely to think that stress ($X^2(2) = 6.05$, p = 0.048), prolonged computer usage ($X^2(2) = 10.05$, p = 0.007), prolonged smartphone usage ($X^2(2) = 11.59$, p = 0.003), and prolonged reading ($X^2(2) = 9.15$, p = 0.010) may worsen symptoms of glaucoma. Compared to ophthalmic non-glaucoma participants, those diagnosed with glaucoma were familiar with the nature of their treatment as they are vigilant of the ability of medications to control glaucoma ($X^2(2) = 19.98$, p <0.001), and that glaucoma treatment is lifelong ($X^2(2) = 32.97$, p <0.001).

Nonetheless, both groups of participants portrayed variability in their awareness towards types of glaucoma, certain risk factors associated with glaucoma (i.e., myopia), effect of

Formatted: Font: Italic

Formatted: Superscript

certain medications on glaucoma development (e.g., corticosteroid containing drops), and the post-operative implications of the surgical management of glaucoma.

Table 2: Responses of participants to knowledge items

	Ophthalmic non-glaucoma patients			Glaucoma patients			
**Items**	Disagree	Neutral	Agree	Disagree	Neutral	Agree	p-value
Glaucoma is a disease that affects the eyes and no other part of the body.	4 (3.3)	34 (28.3)	82 (68.3)	5 (3.7)	16 (11.8)	115 (84.6)	**0.004**
Glaucoma is caused by raised intraocular pressure.	7 (5.8)	54 (45.0)	59 (49.2)	7 (5.1)	27 (19.9)	102 (75.0)	**0.000**
Glaucoma can lead to optic nerve damage and visual disorders.	1 (0.8)	47 (39.2)	72 (60.0)	1 (0.7)	14 (10.3)	121 (89.0)	**0.000**
Glaucoma has more than one type (eg: open angle, closed angle, secondary, developmental)	3 (2.5)	92 (76.7)	25 (20.8)	3 (2.2)	93 (68.4)	40 (29.4)	0.290
Glaucoma can be inherited in the family.	19 (15.8)	55 (45.8)	46 (38.3)	25 (18.4)	47 (34.6)	64 (47.1)	0.182
Glaucoma is more common as you get older.	3 (2.5)	35 (29.2)	82 (68.3)	6 (4.4)	19 (14.0)	111 (81.6)	**0.010**
High blood pressure and diabetes increase the risk of glaucoma.	2 (1.7)	42 (35.0)	76 (63.3)	11 (8.1)	39 (28.7)	86 (63.2)	0.050
People with high myopia or hyperopia are more likely to get glaucoma.	20 (16.7)	58 (48.3)	42 (35.0)	16 (11.8)	72 (52.9)	48 (35.3)	0.507
The use of steroid /eye drops can cause glaucoma.	9 (7.5)	76 (63.3)	35 (29.2)	13 (9.6)	70 (51.5)	53 (39.0)	0.160
Medicines other than eye drops can influence ~~the~~ intraocular pressure.	6 (5.0)	51 (42.5)	63 (52.5)	15 (11.0)	50 (36.8)	71 (52.2)	0.187
Stress can make glaucoma worse.	6 (5.0)	41 (24.2)	73 (60.8)	7 (5.1)	28 (20.6)	101 (74.3)	**0.048**
Using a computer will make glaucoma worse.	4 (3.3)	40 (33.3)	76 (63.3)	13 (9.6)	25 (18.4)	98 (72.1)	**0.007**
Using a smartphone will make glaucoma worse.	4 (3.3)	38 (31.7)	78 (65.0)	14 (10.3)	22 (16.2)	100 (73.5)	**0.003**
Fluorescent lights will make glaucoma worse.	10 (8.3)	59 (49.2)	51 (42.5)	20 (14.7)	62 (45.6)	53 (39.7)	0.286
A lot of reading may make glaucoma worse	11 (9.2)	47 (39.2)	62 (51.7)	13 (9.6)	30 (22.1)	93 (68.4)	**0.010**
High intraocular pressure must be addressed.	0 (0.0)	13 (10.8)	107 (89.2)	0 (0.0)	4 (2.9)	132 (97.1)	**0.021**
Glaucoma can be controlled by treatment.	4 (3.3)	34 (28.3)	82 (68.3)	8 (5.9)	10 (7.4)	118 (86.8)	**0.000**
Early detection and treatment will not slow the course of glaucoma.	70 (58.3)	28 (23.3)	22 (18.3)	82 (60.3)	15 (11.0)	39 (28.7)	**0.013**
It is possible to completely lose vision as a result of laser treatment or surgery for glaucoma.	24 (20.0)	58 (48.3)	38 (31.7)	34 (25.0)	58 (42.6)	44 (32.4)	0.558

The use of eye drops will be redundant if one has had a laser treatment or surgery for glaucoma.	32 (26.7)	60 (50.0)	28 (23.3)	34 (25.0)	60 (44.1)	32 (30.9)	0.393
Follow-up visit ~~in~~ to the clinic is not necessary after the laser treatment or surgery ~~of~~ for glaucoma.	81 (67.5)	19 (15.8)	20 (16.7)	91 (66.9)	13 (9.6)	32 (23.5)	0.175
Regular check-ups are not necessary for glaucoma patients.	89 (74.2)	17 (14.2)	14 (11.7)	106 (77.9)	7 (5.1)	23 (16.9)	**0.032**
Even if the intraocular pressure is under control, the visual field has to be checked.	1 (0.8)	34 (28.3)	85 (70.8)	2 (1.5)	18 (13.2)	116 (85.3)	**0.011**
A patient should always tell the ophthalmologist which other medicines (s)he is taking.	2 (1.7)	8 (6.7)	110 (91.7)	0 (0.0)	4 (2.9)	132 (97.1)	0.114
A patient should always tell the ophthalmologist which other diseases (s)he has.	2 (1.7)	9 (7.5)	109 (90.8)	0 (0.0)	3 (2.2)	133 (97.8)	**0.041**
Treatment for glaucoma is lifelong.	23 (19.2)	69 (57.5)	28 (23.3)	13 (9.6)	43 (31.6)	80 (58.8)	**0.000**
Vision loss is reversible after initiating glaucoma treatment.	21 (17.5)	57 (47.5)	42 (35.0)	60 (44.1)	42 (30.9)	34 (25.0)	**0.000**

Most participants were aware of glaucoma symptoms with the exception of convulsive seizures (9.0%). Moreover, only 27.3% were aware that glaucoma can manifest as an asymptomatic entity. Participants with glaucoma were significantly more likely to report vision loss ($p = 0.008$), and pain, redness, nausea, and vomiting ($X^2(1) = 12.11$, $p = 0.001$) as symptoms of glaucoma than their ophthalmic non-glaucoma counterparts. Similarly, the treatment of glaucoma with medications is significantly more recognized by participants with glaucoma ($X^2(1) = 8.11$, $p = 0.006$). The least reported modality of treatment among both groups was surgery (57.5%, and 69.1%).

The least ~~common~~ commonly recognized complications of glaucoma treatment were dyspnea (10.9%), bradycardia (12.1%), and elongation of eyelashes (18.0%). In terms of sources of information, participants with glaucoma are significantly more dependent on ophthalmologists for disease-specific information ($X^2(1) = 80.62$, $p < 0.001$). On the other hand, ophthalmic non-glaucoma participants are significantly more likely to rely on close social circles ($X^2(2) = 15.28$, $p < 0.001$). **Table 3** describes knowledge about glaucoma's presentation and its treatment.

Table 3: Awareness of risk factors, treatment options, and difficulties associated with glaucoma

		Ophthalmic non-glaucoma patients	Glaucoma patients	p-value*
Signs & symptoms of glaucoma				
	Asymptomatic	29 (24.2)	41 (30.1)	0.326
	Reduced visual acuity	106 (88.3)	123 (90.4)	0.685
	Narrow field of vision	62 (51.7)	85 (62.5)	0.100
	Vision loss	55 (45.8)	85 (62.5)	**0.008**
	Convulsive seizures	11 (9.2)	12 (8.8)	1.000

	Pain, redness, nausea, and vomiting	41 (34.2)	76 (55.9)	**0.001**
Treatment options for glaucoma				
	Medications	75 (62.5)	107 (78.7)	**0.006**
	Surgical procedures	93 (77.5)	118 (86.8)	0.070
	Laser therapy	69 (57.5)	94 (69.1)	0.068
Complications of treatment				
	Stinging and burning	90 (75.0)	96 (70.6)	0.483
	Blurred vision	87 (72.5)	96 (70.6)	0.782
	Discoloration of iris	29 (24.2)	29 (21.3)	0.654
	Dyspnea	10 (8.3)	18 (13.2)	0.234
	Tachypnea	15 (12.5)	16 (11.8)	1.000
	Elongation of lashes	9 (7.5)	37 (27.2)	**0.000**
	Redness, dryness, and itching	64 (53.3)	100 (73.5)	**0.001**
Source of information				
	Ophthalmologists	35 (29.2)	115 (84.6)	**0.000**
	Non-ophthalmologists	10 (8.3)	8 (5.9)	0.472
	Friends and family	53 (44.2)	29 (21.3)	**0.000**
	TV/Newspapers	32 (26.7)	27 (19.9)	0.234
	Internet	45 (37.5)	56 (41.2)	0.609
	Social Media	21 (17.5)	23 (16.9)	1.000
Difficulties due to ophthalmic conditions				
	Identifying persons	27 (22.5)	54 (39.7)	**0.005**
	Reading	39 (32.5)	73 (53.7)	**0.001**
	Using stairs	21 (17.5)	44 (32.4)	**0.009**
	Watching TV	31 (25.8)	54 (39.7)	**0.024**
	Driving	30 (25.0)	52 (38.2)	**0.031**
	Use social media	23 (19.2)	25 (25.7)	0.233
	Walking alone	14 (11.7)	32 (23.5)	**0.015**
	Differentiating colors and clothes	20 (16.7)	28 (20.6)	0.521
	No difficulties	70 (58.3)	43 (31.6)	**0.000**

*Conducted using the chi-squared test

Formatted Table

Compared to their ophthalmic non-glaucoma counterparts, those diagnosed with glaucoma face significantly more daily life difficulties due to their ophthalmic disease (p <0.001). Such difficulties include identifying persons (p = 0.005), reading (p = 0.001), using the stairs (p = 0.009), driving (p = 0.031), walking alone (p = 0.015), among others. Overall, most participants request that more information on the pathophysiology/progression (83.6%), treatment (68.4%) of glaucoma, and other practical advice on how to manage disease fluctuations (71.1%) are needed to improve awareness and knowledge on of glaucoma.

Univariate analysis shows that participants with glaucoma have significantly higher knowledge scores (t(254) = 4.31, p <0.001) and were able to recognize more glaucoma symptoms than their non-glaucoma counterparts (t(254) = 3.18, p = 0.002). Similarly, those with a positive family history of glaucoma displayed higher knowledge (t(249) = 2.81, p = 0.005) and a relatively higher ability to recognize glaucoma symptoms (t(249) = 2.07, p = 0.039). Knowledge scores did not differ between genders, area of residence, educational status, income level, or medical history (refer to **table 4**). Moreover, the ability to recognize more symptoms and more treatment-related complications were was positively correlated with higher knowledge scores (r = 0.302, p <0.001; r = 0.285, p <0.001, respectively). However, age (p = 0.130), number of clinic visits per month (p = 0.410), and number of clinic visits per year (p = 0.412) were not significantly correlated with knowledge scores.

Table 4: Factors affecting glaucoma knowledge scores

		Knowledge Score	p-value	Symptom recognition	p-value	Adverse effect recognition	p-value
Disease Status			**0.000**		**0.002**		0.074
	Ophthalmic non-glaucoma patients	14.6 ± 6.1		2.5 ± 1.4		2.5 ± 1.6	
	Glaucoma patients	17.5 ± 4.1		3.1 ± 1.4		2.9 ± 1.5	
Biological Sex			0.442		**0.028**		0.204
	Male	16.4 ± 5.3		3.0 ± 1.5		2.8 ± 1.7	
	Female	15.8 ± 5.4		2.6 ± 1.4		2.6 ± 1.4	
Residence			0.856		0.929		0.642

	Amman (Capital)	16.1 ± 5.3		2.8 ± 1.4		2.7 ± 1.6	
	Central	15.8 ± 6.1		2.8 ± 1.4		2.7 ± 1.4	
	South	17.1 ± 2.8		2.6 ± 1.3		2.3 ± 1.1	
	North	16.6 ± 4.1		2.6 ± 1.7		2.3 ± 1.1	
Education			0.076		0.440		0.114
	Illiterate	16.3 ± 6.0		3.1 ± 1.4		3.3 ± 1.6	
	Primary	16.2 ± 4.6		3.1 ± 1.3		2.6 ± 1.3	
	Highschool	14.7 ± 6.2		2.7 ± 1.6		2.6 ± 1.6	
	Bachelors	16.8 ± 4.6		2.7 ± 1.4		2.5 ± 1.5	
	Master's +	17.4 ± 5.1		3.0 ± 1.4		3.2 ± 1.7	
Income			0.077		0.460		0.110
	< 499	16.0 ± 5.4		2.9 ± 1.5		2.7 ± 1.5	
	500 – 999	16.4 ± 5.1		2.6 ± 1.4		2.5 ± 1.5	
	1000 – 1999	15.1 ± 5.3		2.9 ± 1.1		2.4 ± 1.2	
	> 2000	22.0 ± 1.0		3.4 ± 0.8		4.2 ± 1.8	
Comorbidities			0.346		0.448		0.938
	No	15.8 ± 5.9		2.9 ± 1.4		2.7 ± 1.6	
	Yes	16.4 ± 4.7		2.7 ± 1.4		2.7 ± 1.5	
Smoking history			0.699		0.754		0.471
	No	16.1 ± 5.3		2.8 ± 1.5		2.7 ± 1.5	
	Yes	16.4 ± 5.6		2.9 ± 1.4		2.8 ± 1.5	
Family history of glaucoma			**0.005**		**0.039**		0.154
	No	15.5 ± 5.8		2.7 ± 1.4		2.6 ± 1.5	
	Yes	17.5 ± 3.9		3.1 ± 1.5		2.9 ± 1.6	

Multivariate linear regression demonstrates that a family history of glaucoma (B: 1.539; 95%CI: 0.078 – 3.000; p-value: 0.039), higher symptom recognition score (B: 0.624; 95%CI: 0.083 – 1.164, p-value: 0.024), relying on ophthalmologists (B: 2.584; 95%CI: 0.983 – 4.185; p-value: 0.002), and the internet (B: 2.364; 95%CI: 0.845 – 3.883: p-value: 0.002) for glaucoma-related information are positive predictors of higher knowledge scores. **Table 5** delineates ~~that~~ the linear regression model for knowledge scores.

Table 5: Predictors of knowledge score

	**Linear regression for knowledge score**				
	**p-value**	**B**	**ß**	**Lower 95% CI for (B)**	**Upper 95% CI for (B)**
Age	0.390	0.019	0.067	-0.025	0.064
Female gender	0.334	0.672	0.065	-0.696	2.041
Male gender (**Reference**)	—	—	—	—	—
Family history of Glaucoma	**0.039**	1.539	0.138	0.078	3.000

Formatted Table

Formatted: Font: Bold

Formatted Table

No family history of Glaucoma **(Reference)**	—	—	—	—	—
Positive presence of comorbidities	0.712	0.290	0.028	-1.259	1.840
Smoking	0.425	0.651	0.052	-0.957	2.260
No smoking **(Reference)**	—	—	—	—	—
Clinic Visit Per Year	0.724	-0.019	-0.025	-0.122	0.085
Symptom recognition score	**0.024**	0.624	0.175	0.083	1.164
Adverse effects recognition score	0.568	0.163	0.048	-0.399	0.724
Number of faced difficulties due to ophthalmic disease	0.445	0.108	0.055	-0.170	0.386
**Sources of information**					
Ophthalmologists	**0.002**	2.584	0.239	0.983	4.185
Non-Ophthalmologists	0.376	1.324	0.060	-1.621	4.268
Friends and Family	0.832	-0.174	-0.015	-1.783	1.436
TV/Newspapers	0.696	-0.344	-0.027	-2.081	1.392
Internet	**0.002**	2.364	0.227	0.845	3.883
Social Media	0.518	-0.631	-0.047	-2.554	1.292

Formatted: Font: Bold

Formatted Table

Formatted: Font: Bold

Formatted Table

Discussion

Our study demonstrated that glaucoma knowledge among Jordanian ophthalmic patients is rather underwhelming and extremely variable. Knowledge deficiencies are most pronounced in areas such as risk factors, neurological symptoms, adverse effects of over-the-counter medications of glaucoma, and post-operative management and expectations. Additionally, it appears that the extent of knowledge of recruited participants is only influenced by their glaucoma diagnosis and family history of glaucoma as all sociodemographic factors did not significantly impact overall knowledge.

~~Among Jordanians, glaucoma is the third leading cause of severe bilateral blindness [24]. It is well established that glaucoma-induced blindness is influenced by poor patient compliance~~

[25]; a factor that is associated with the patients' knowledge and comprehension of their disease as delineated in multiple reports [5]. Moreover, an incomplete understanding of glaucoma disease dynamics may exacerbate psychological burdens among patients or leave them oblivious to the blinding nature of glaucoma [12]. Therefore, knowledge of glaucoma could be of vital importance as a target of interventions aiming to improve the lifestyles among those with glaucoma, particularly high-risk groups (e.g., geriatrics). Gatwood et al., (2022) demonstrates that disease-related educational interventions improve adherence to glaucoma treatment [26].

The level of knowledge of glaucoma portrays significant variability across different reports throughout the globe. Local Jordanian reports show that while Jordanian patients visiting Jordan University Hospital were mostly aware of glaucoma, only 34.1% were able to define it accurately [26]. In contrast, only 38.8% of the Jordanian population was aware of glaucoma [18]. In terms of regional literature, a Saudi Arabian report demonstrates that patients with glaucoma exhibit poor knowledge, attitudes, and practices towards their disease [27]. Similarly, a survey of Iraqi ophthalmic patients with disease-induced blindness demonstrated that lack of awareness is the most dominant factor affecting patients with glaucoma [28]. An Iranian population-based survey demonstrated that only 46.6% demonstrated glaucoma awareness while only 19.2% were able to correctly define the disease [29]. Moreover, among Egyptian adolescents with glaucoma, satisfactory knowledge was as low as 12% prior to the initiation of an educational program.[30] Asian countries, the likes of Nepal and India, demonstrated low rates of glaucoma awareness knowledge among hospital presenting patients ranging from 1.9% to 27% [31–33]. China, on the other hand, demonstrated that 51.6% of glaucoma patients visiting a tertiary university hospital were aware of their disease [34].

Great variance is noted among African nations in terms of glaucoma awareness as rates range from 74% among Ghanian patients with glaucoma visiting a tertiary teaching hospital, 36.8% among Nigerians with glaucoma, 46.7% among Ethiopian glaucoma patients, to 2.4% among Ethiopian patients visiting the ophthalmic outpatient clinics [35–38]. On the other hand, Western nations such as Austria, Germany, the United States, Australia, and the United Kingdom display far superior rates of glaucoma knowledge ranging from 51% to 93% among the public and patients visiting eye clinics [19,39–41]. Nonetheless, marked variability does exist among such a report in which an investigation of Swiss patients with glaucoma demonstrated low levels of glaucoma knowledge as only 28% were able to define their disease [5].

While such variance among different studies could be attributed to differences between studied populations in terms of sociodemographic or clinical characteristics, inherent methodological differences make a direct comparison between any two sets of reports unfeasible. There seems to be an overlap, or a rather failure of distinction, between knowledge and awareness [22]. Across the aforementioned studies, measures of knowledge assessment were dramatically different in terms of content, format (e.g., number and type of questions), and definitions of proper knowledge, most of which were made based on arbitrary and/or convenient basis [42]. Furthermore, those questionnaire-based measures were not always developed ~~in the most rigorous manner~~rigorously as development discrepancies (e.g., presence or absence of pilot testing, expert group consultation, and extensive literature review of similar studies) could lead to the heterogenous observation of glaucoma-targeted knowledge.

We demonstrated that glaucoma patients are more knowledgeable about glaucoma in comparison to ophthalmic non-glaucoma counterparts~~;~~, yet the knowledge among the two groups was relatively poor. Such observation is fairly established within the literature and is

expected as those with established glaucoma diagnosis receive a greater fund of disease-specific knowledge from their doctors or close social circles at far higher frequencies of contact [23]. Literature shows that durations of glaucoma and follow-up are significantly associated with improved levels of knowledge [12,43]. Another plausible explanation is the reliance of ophthalmic non-glaucoma patients on friends and family for glaucoma-related information, as well as lack of clinical visits, both of which may predispose them to inaccurate or outdated information. Such reliance is rather interesting as all recruited participants were from a hospital setting which may indicate a gap of trust between patients and healthcare providers within our tertiary hospital. Reliance on "friends and family" among the public or ophthalmic non-glaucoma patients has been reported in a German survey, which demonstrated fairly poor levels of glaucoma knowledge [41]. Hassan et al., (2021) mentioned that the large number of patients seen by ~~the~~ doctors in primary ~~health-care~~healthcare clinics as well as in ophthalmology clinics in Jordan results in less doctor-patient time in the clinic which consequently makes doctor-patient encounters less informative [26]. Increasing the number of serving physicians in ophthalmology clinics and fortifying their communication skills may ensure the appropriate delivery of vital glaucoma-related information to patients, however, this solution might be ~~over ambitious~~over-ambitious in ~~resource scarce~~resource-scarce institutions and developing nations.

Amongst the literature, several factors were implicated to have influenced glaucoma-related knowledge such as age, education, occupation, income, accessibility of ophthalmic care, duration of glaucoma, compliance to medications, family history of glaucoma, and type of glaucoma [20,22,34]. Chen et al., (2022) ~~demonstrates~~ demonstrate a compressive review of such factors and ~~shows~~ show that lack of conformity between the impact of different factors on glaucoma knowledge can be explained by differences in study design, characteristics of study populations, and methods of analysis [34]. In agreement with our investigation, several

studies reported that participants with a family history of glaucoma were significantly more knowledgeable about their condition [12,26,41]. Such might be the case because having a family member with glaucoma may provoke the individual to search for more information and/or gather information during their appointment with the eye care professional [31]. Nonetheless, this association is not always consistent across the literature as family history of glaucoma failed to improve levels of glaucoma knowledge among Swiss glaucoma patients visiting tertiary glaucoma clinics [5]. Contrary to the literary consensus that shows higher educational standards associated with more glaucoma knowledge [21,25,34], educational level was not significantly associated with glaucoma knowledge among both groups of studied participants. This can be attributed to weakness in the Jordanian health-related educational programs for secondary and higher levels of education [26]. Additionally, age did not significantly predict knowledge scores among included participants. Interestingly, age was predominantly a negative predictor of glaucoma knowledge within the literature. A variety of factors could've influenced such an association such as the low levels of health literacy in older individuals, neurological disease progression, or the inherent technological bias exhibited by younger participants able to fully utilize the internet to gain disease-related knowledge [34,44].

Since glaucoma is a major contributor to vision loss in Jordan and worldwide, lack of knowledge about glaucoma makes improving information about this disease a necessity ~~to~~ for Jordanian and global communities. Achieving acceptable ~~long term~~long-term visual outcomes in glaucoma relies on controlling the eye pressure which is dependent on the patient adhering to the treatment regimen [45]; a dimension that can be ~~improved~~ improved by improving patient knowledge about glaucoma [46,47]. This can be achieved through using a ~~patient held~~patient-held record (e.g., 'Glaucoma Logbook'), which is an A5-sized book containing some basic and important information about glaucoma, patient information, details

of the treating medical team, intraocular pressure, medications, and details of support services. Such a method proved to be effective in increasing patients' knowledge to of glaucoma among those treated for glaucoma or ocular hypertension [48]. Tackling the issue of poor patient knowledge is of great importance as both studied groups were able to identify less than 50% of the proposed adverse effects associated with glaucoma medications. Considering the wide-spectrumwide spectrum of systemic side effects associated with beta-blockers and alpha-agonists, targeted solutions should focus on such deficiency [12].

Institution wiseInstitution-wise, a multitude of research-proven interventions could be implemented in order toto tackle these gaps of knowledge. Chen et al., (2009) demonstrated the impact of creating a glaucoma club on recruited participants [49]. Participants within the club had significantly higher levels of glaucoma knowledge, had improved disease-cognitiondisease cognition, experienced improved medications compliancecompliance to medications, and lived healthier lives. Li et al., (2018) showed that a mobile-based education program, administered through social media (e.g., WeChat), was effective in improving patients' education towards glaucoma and enhanced their willingness to utilize electronic services for their follow-up [23]. The authors argue that such programs, not only enhance public awareness, but also improve treatment adherence and clinical outcomes. Tatham et al., (2019) demonstrated that virtual clinic visits yielded the same, yet defective, levels of glaucoma knowledge compared to those attending face-to-face clinics [50]. This may suggest the adoption and enhancement of a telemedicine program centered around patient education may impact knowledge with lesser long-term costs. Finally, the formulation of a patient-centered educational program was proven effective in enhancing the knowledge of patients with congenital and secondary glaucoma [30]. Such a program may take different forms ranging from seminars, written publications (e.g., brochures and leaflets), and video materials, to theory-based practical sessions given throughout a patient's clinical visit. Peralta

et al., (2018) stressed on the importance of such programs to increase knowledge and raise awareness about glaucoma [42].

The burden of ameliorating glaucoma awareness and knowledge should also be shared with governing bodies and health policymakers. Our multivariate analysis demonstrated that ophthalmologists and the internet are associated with increased levels of knowledge; an observation that was documented in China [34]. Concerned governmental authorities and health policymakers should increase the public's awareness and knowledge by allocating more resources towards enhancing ophthalmology practice and ~~utilize~~ utilizing internet-based solutions to reach a wide audience. Improving awareness was shown to help in early glaucoma detection, thus better control and recognition of its complications [25]. Moreover, early detection, control, and treatment will mitigate healthcare costs of patients over the long-term and alleviate the medical burden ~~through~~ by encouraging patients to seek medical treatment [24].

This study is not devoid of limitations. The cross-sectional nature of its design is already associated with a handful of biases including sampling bias that may result in a snapshot of the population that may not reflect the original sampling pool. The small sample size might have impacted ~~that~~ the statistical robustness by which knowledge scores are differentiated. Moreover, the study examines glaucoma knowledge in a different, yet more comprehensive, manner ~~to~~ than the available literature thus it might underestimate its rates of glaucoma awareness. Moreover, data may be subjected to recall bias due to the self-reporting nature of the questionnaire. Finally, we did not investigate compliance ~~to~~ with medications which could be affected by glaucoma-related knowledge. Nonetheless, the strength of the study lies in its usage of a rigorously designed and validated questionnaire to evaluate glaucoma-related knowledge. Also, the study's sample of glaucoma patients closely resembles the overall

number of glaucoma patients treated at the JUH ophthalmology clinics thus a high internal validity.

Conclusion

In light of this investigation, we have demonstrated that ophthalmic patients display mediocre levels of glaucoma knowledge. Raising awareness through various programs and interventions may improve the lifestyles of patients with glaucoma, which may result in ~~a~~ long-term mitigation of the economic burden associated with tending to the chronic and severe complications of such disease. Concerned authorities should project their interventions through ophthalmologists and internet-based platforms due to their profound effect in shaping glaucoma-related knowledge. ~~Such interventions must account for the pathophysiology of glaucoma and its treatment dynamics, especially for those with neurological diseases.~~

Acknowledgments

None

Declaration of interest

~~The authors report there are no competing interests to declare.~~The authors have declared that no competing interests exist.

Funding disclosure statement

The author(s) received no specific funding for this work

Author contributions

Conceptualization: Sana' Muhsen, Abdallah Al-Ani

Data Curation: Leen Al-Huneidy

Formal analysis: Abdallah Al-Ani, Leen Al-Huneidy

Methodology: Abdallah Al-Ani, Lina AlQirem, Leen Al-Huneidy, Ward Maaita

Supervision: Sana' Muhsen

Writing – original draft: All authors

Writing – review & editing: Sana' Muhsen, Abdallah Al-Ani, Leen Al-Huneidy

Supporting information

S1 Data. Data from the administered survey

S2 File. Data collection instrument (questionnaire)

References

1. Weinreb RN, Aung T, Medeiros FA. The pathophysiology and treatment of glaucoma: A review. JAMA - J Am Med Assoc. 2014;311: 1901–1911. doi:10.1001/jama.2014.3192

2. Uche NJ, Udeh NN, Chuka-Okosa CM, Kizor-Akaraiwe NN, Uche EO. Glaucoma care and follow-up in sub-Saharan Africa: Is there a need for modification of counselling practices to improve awareness, knowledge and treatment acceptance profiles? A prospective cross-sectional study. Int Ophthalmol. 2020;40: 1539–1546. doi:10.1007/s10792-020-01323-6

3. Tham YC, Li X, Wong TY, Quigley HA, Aung T, Cheng CY. Global prevalence of glaucoma and projections of glaucoma burden through 2040: A systematic review and meta-analysis. Ophthalmology. 2014;121: 2081–2090. doi:10.1016/j.ophtha.2014.05.013

4. Sood D, Sood S, Sood I, Kumar D, Sood R, Sood NN. Glaucoma awareness amongst glaucoma patients seeking a second opinion. Int Ophthalmol. 2018;38: 1441–1449. doi:10.1007/s10792-017-0604-5

5. Mansouri K, Iliev ME, Rohrer K, Shaarawy T. Compliance and knowledge about glaucoma in patients at tertiary glaucoma units. Int Ophthalmol. 2011;31: 369–376. doi:10.1007/s10792-011-9468-2

6. Noertjojo K, Maberley D, Bassett K, Courtright P. Awareness of eye diseases and risk factors: Identifying needs for health education and promotion in Canada. Can J Ophthalmol. 2006;41: 617–623. doi:10.1016/S0008-4182(06)80035-9

7. Dandona R. Awareness of eye diseases in an urban population in southern India. Bull World Health Organ. 2001;79: 96–102.

8. Fabjani S, Richter-Müksch S, Cakmak N, Markstaller M, Weingessel B, Vécsei-Marlovits VP. Informationsstand über die Erkrankung Glaukom in Wien. Wien Klin Wochenschr. 2015;127: 612–618. doi:10.1007/s00508-014-0687-1

9. Hoevenaars JGMM, Schouten JSAG, Van Den Borne B, Beckers HJM, Webers CAB. Knowledge base and preferred methods of obtaining knowledge of glaucoma patients. Eur J Ophthalmol. 2005;15: 32–40. doi:10.1177/112067210501500106

10. Hoevenaars JGMM, Schouten JSAG, van den Borne B, Beckers HJM, Webers CAB. Socioeconomic differences in glaucoma patients' knowledge, need for information and expectations of treatments. Acta Ophthalmol Scand. 2006;84: 84–91. doi:10.1111/j.1600-0420.2005.00587.x

11. Li J, Huang W, Gao J, Li D, Xu L, Huang J. Impact of Mobile-Based Health Education on the Awareness and Knowledge of Glaucoma in Chinese Patients. Telemed E-Health. 2019;25: 455–461. doi:10.1089/tmj.2018.0123

12. Danesh-Meyer H V., Deva NC, Slight C, Tan YW, Tarr K, Carroll SC, et al. What do people with glaucoma know about their condition? A comparative cross-sectional incidence and prevalence survey. Clin Experiment Ophthalmol. 2008;36: 13–18. doi:10.1111/j.1442-9071.2007.01624.x

13. Adegbehingbe BO, Bisiriyu LA. Knowledge, attitudes, and self care practices associated with glaucoma among hospital workers in Ile-Ife, Osun State, Nigeria. Tanzan J Health Res. 2008;10: 240–245. doi:10.4314/thrb.v10i4.45080

14. Saw SM, Gazzard G, Friedman D, Foster PJ, Devereux JG, Wong ML, et al. Awareness of glaucoma, and health beliefs of patients suffering primary acute angle closure. Br J Ophthalmol. 2003;87: 446–449. doi:10.1136/bjo.87.4.446

15. Gatwood J, Brooks C, Meacham R, Abou-Rahma J, Cernasev A, Brown E, et al. Facilitators and Barriers to Glaucoma Medication Adherence. J Glaucoma. 2022;31: 31–36. doi:10.1097/IJG.0000000000001965

16. Trevethan R. Deconstructing and Assessing Knowledge and Awareness in Public Health Research. Front Public Health. 2017;5: 194. doi:10.3389/fpubh.2017.00194

17. Rabiu MM, Al Bdour MD, Abu Ameerh MA, Jadoon MZ. Prevalence of blindness and diabetic retinopathy in northern Jordan. Eur J Ophthalmol. 2015;25: 320–327. doi:10.5301/ejo.5000557

18. Haddad MF, Bakkar MM, Abdo N. Public awareness of common eye diseases in Jordan. BMC Ophthalmol. 2017;17: 177. doi:10.1186/s12886-017-0575-3

19. Fabjani S, Richter-Müksch S, Cakmak N, Markstaller M, Weingessel B, Vécsei-Marlovits VP. Informationsstand über die Erkrankung Glaukom in Wien. Wien Klin Wochenschr. 2015;127: 612–618. doi:10.1007/s00508-014-0687-1

20. Hoevenaars JGMM, Schouten JSAG, van den Borne B, Beckers HJM, Webers CAB. Socioeconomic differences in glaucoma patients' knowledge, need for information and expectations of treatments. Acta Ophthalmol Scand. 2006;84: 84–91. doi:10.1111/j.1600-0420.2005.00587.x

21. Hoevenaars JGMM, Schouten JSAG, Van Den Borne B, Beckers HJM, Webers CAB. Knowledge base and preferred methods of obtaining knowledge of glaucoma patients. Eur J Ophthalmol. 2005;15: 32–40. doi:10.1177/112067210501500106

22. Uche NJ, Udeh NN, Chuka-Okosa CM, Kizor-Akaraiwe NN, Uche EO. Glaucoma care and follow-up in sub-Saharan Africa: Is there a need for modification of counselling practices to improve awareness, knowledge and treatment acceptance profiles? A prospective cross-sectional study. Int Ophthalmol. 2020;40: 1539–1546. doi:10.1007/s10792-020-01323-6

23. Li J, Huang W, Gao J, Li D, Xu L, Huang J. Impact of Mobile-Based Health Education on the Awareness and Knowledge of Glaucoma in Chinese Patients. Telemed E-Health. 2019;25: 455–461. doi:10.1089/tmj.2018.0123

24. Saw SM, Gazzard G, Friedman D, Foster PJ, Devereux JG, Wong ML, et al. Awareness of glaucoma, and health beliefs of patients suffering primary acute angle closure. Br J Ophthalmol. 2003;87: 446–449. doi:10.1136/bjo.87.4.446

25. Sood D, Sood S, Sood I, Kumar D, Sood R, Sood NN. Glaucoma awareness amongst glaucoma patients seeking a second opinion. Int Ophthalmol. 2018;38: 1441–1449. doi:10.1007/s10792-017-0604-5

26. Hassan DWA, Alsibai BA, Alhouri AN, Alhajeh RY, Suleiman AA, Al Bdour MD. Awareness and knowledge about glaucoma among patients attending outpatient clinics of Jordan University Hospital. 2021. doi:10.4103/1319-4534.322618

27. Al Rashed WA, Bin Shihah AS, Alhomoud AS, Alharbi MM, Shadid AM, Alghamdi MA, et al. Knowledge, attitude, and practice toward glaucoma and its management among adult Saudi patients. Saudi J Ophthalmol Off J Saudi Ophthalmol Soc. 2020;34: 261–265. doi:10.4103/1319-4534.322596

28. Al-Shakarchi FI. Blindness in iraq: leading causes, target patients, and barriers to treatment. Middle East Afr J Ophthalmol. 2011;18: 199–203. doi:10.4103/0974-9233.84044

29. Katibeh M, Ziaei H, Panah E, Moein H-R, Hosseini S, Kalantarion M, et al. Knowledge and Awareness of Age Related Eye Diseases: a Population-Based Survey. J Ophthalmic Vis Res. 2014;9: 223–231.

30. Mohamed EA, Bayoumi OR, Draz SF. Impact of an educational programme on knowledge, beliefs, practices and expectations about care among adolescent glaucoma patients in Cairo. East Mediterr Health J Rev Sante Mediterr Orient Al-Majallah Al-Sihhiyah Li-Sharq Al-Mutawassit. 2011;17: 960–968. doi:10.26719/2011.17.12.960

31. Gyawali R, Sarkar N. Glaucoma awareness in a hospital presenting population in eastern Nepal. J Glaucoma. 2014;23: 594–598. doi:10.1097/IJG.0b013e31828700de

32. Rewri P, Kakkar M. Awareness, knowledge, and practice: A survey of glaucoma in north Indian rural residents. Indian J Ophthalmol. 2014;62: 482–486. doi:10.4103/0301-4738.132105

33. Maharana PK, Rai VG, Pattebahadur R, Singhi S, Chauhan AK. Awareness and Knowledge of Glaucoma in Central India: A Hospital-Based Study. Asia-Pac J Ophthalmol Phila Pa. 2017;6: 243–249. doi:10.22608/APO.2015166

34. Chen X, Zhong Y-L, Chen Q, Tao Y-J, Yang W-Y, Niu Z-Q, et al. Knowledge of glaucoma and associated factors among primary glaucoma patients in Kunming, China. BMC Ophthalmol. 2022;22: 95. doi:10.1186/s12886-022-02322-0

35. Kizor-Akaraiwe NN, Monye HI, Okeke S. Awareness and knowledge about glaucoma and proportion of people with glaucoma in an urban outreach programme in Southeast Nigeria. BMJ Open Ophthalmol. 2017;1: 1–10. doi:10.1136/bmjophth-2016-000018

36. Tenkir A, Solomon B, Deribew A. Glaucoma awareness among people attending ophthalmic outreach services in Southwestern Ethiopia. BMC Ophthalmol. 2010;10. doi:10.1186/1471-2415-10-17

37. Alemu DS, Gudeta AD, Gebreselassie KL. Awareness and knowledge of glaucoma and associated factors among adults: a cross sectional study in Gondar Town, Northwest Ethiopia. BMC Ophthalmol. 2017;17: 154. doi:10.1186/s12886-017-0542-z

38. Nkum G, Lartey S, Frimpong C, Micah F, Nkum B. Awareness and Knowledge of Glaucoma Among Adult Patients at the Eye Clinic of a Teaching Hospital. Ghana Med J. 2015;49: 195–199. doi:10.4314/gmj.v49i3.11

39. Gasch AT, Wang P, Pasquale LR. Determinants of glaucoma awareness in a general eye clinic. Ophthalmology. 2000;107: 303–308. doi:10.1016/S0161-6420(99)00076-7

40. Baker H, Cousens SN, Murdoch IE. Poor public health knowledge about glaucoma: Fact or fiction. Eye. 2010;24: 653–657. doi:10.1038/eye.2009.155

41. Pfeiffer N, Krieglstein GK, Wellek S. Knowledge about glaucoma in the unselected population: A German survey. J Glaucoma. 2002;11: 458–463. doi:10.1097/00061198-200210000-00016

42. Peralta E, Muir KW, Rosdahl JA. Systematic Review of Knowledge Assessments for Glaucoma Patients. Semin Ophthalmol. 2018;33: 377–388. doi:10.1080/08820538.2016.1247180

43. Gray TA, Fenerty C, Harper R, Lee A, Spencer AF, Campbell M, et al. Preliminary survey of educational support for patients prescribed ocular hypotensive therapy. Eye. 2010;24: 1777–1786. doi:10.1038/eye.2010.121

44. Schönfeld MS, Pfisterer-Heise S, Bergelt C. Self-reported health literacy and medication adherence in older adults: a systematic review. BMJ Open. 2021;11: e056307. doi:10.1136/bmjopen-2021-056307

45. Meyers MA. Distribution of intra-abdominal malignant seeding: dependency on dynamics of flow of ascitic fluid. Am J Roentgenol Radium Ther Nucl Med. 1973;119: 198–206. doi:10.2214/ajr.119.1.198

46. Kashiwagi K, Tsukahara S. Impact of patient access to internet health records on glaucoma medication: Randomized controlled trial. J Med Internet Res. 2014;16: 1–16. doi:10.2196/jmir.2795

47. Attebo K, Mitchell P, Cumming R, Smith W. Knowledge and beliefs about common eye diseases. Aust N Z J Ophthalmol. 1997;25: 283–287. doi:10.1111/j.1442-9071.1997.tb01516.x

48. Daly R, Agarwal PK. Is Patient Knowledge of Glaucoma Improved by Using a Glaucoma Logbook? J Glaucoma. 2018;27: 470–475. doi:10.1097/IJG.0000000000000943

49. Chen X, Chen Y, Sun X. Notable role of glaucoma club on patients' knowledge of glaucoma. Clin Experiment Ophthalmol. 2009;37: 590–594. doi:10.1111/j.1442-9071.2009.02101.x

50. Tatham AJ, Ali AM, Hillier N. Knowledge of Glaucoma Among Patients Attending Virtual and Face-to-Face Glaucoma Clinics. J Glaucoma. 2021;30: 325–331. doi:10.1097/IJG.0000000000001758

Rebuttal Letter

On behalf of myself and all authors within this study, we thank the panel of reviewers for their extensive, shrewd, and informative comments. Any and all changes requested implied through your comments were considered and worked upon in order to strengthen the framework of our manuscript and render it suitable for publication in your respected journal. We would like to thank you for all of your efforts. The new manuscript file now has an expanded introduction, methodology, results, and discussion sections, all based on your comments. We hope that these expansions address your concerns and further validate our work. We immensely appreciate giving us the opportunity to revise our manuscript. We would like to thank the editorial team and the associated reviewers for their efforts. We hope that everyone is staying safe and well.

Editorial comments

1. Please ensure that your manuscript meets PLOS ONE's style requirements, including those for file naming.

   a. **Response**: The manuscript style was edited to match PLOS ONE’s style requirements.

2. Please provide additional details regarding participant consent. In the ethics statement in the Methods and online submission information, please ensure that you have specified (1) whether consent was informed and (2) what type you obtained (for instance, written or verbal, and if verbal, how it was documented and witnessed). If your study included minors, state whether you obtained consent from parents or guardians. If the need for consent was waived by the ethics committee, please include this information.

   a. **Response**: The ethical considerations section was updated to accommodate for the abovementioned concerns. The methodology section states that only participants 18 years and older were recruited; therefore, no minors were included in the study.

3. You indicated that you had ethical approval for your study. Please clarify whether minors (participants under the age of 18 years) were included in this study. If yes, in your Methods section, please ensure you have also stated whether you obtained consent from parents or guardians of the minors included in the study or whether the research ethics committee or IRB specifically waived the need for their consent.

   a. **Response**: The ethical considerations section was updated to accommodate for the abovementioned concerns. The methodology section states that only participants 18 years and older were recruited; therefore, no minors were included in the study.

4. Please review your reference list to ensure that it is complete and correct. If you have cited papers that have been retracted, please include the rationale for doing so in the manuscript text, or remove these references and replace them with relevant current references. Any changes to the reference list should be mentioned in the rebuttal letter that accompanies your revised manuscript. If you need to cite a retracted article, indicate the article’s retracted status in the References list and also include a citation and full reference for the retraction notice.

   a. **Response**: The reference list was checked and the any added references resulted from dealing with reviewers’ comments were noted at in the rebuttal letter.

Reviewer comments

Abstract

1. Why compare to ophthalmic patients? this demography is expected to demonstrate some knowledge a eye patients. so maybe the public at large would be a better option especially considering the use of self administered questionnaire

    a. **Response**: Dear respected reviewer, the data collection was designed in such a fashion in order to concentrate efforts into the recruitment of patients within one space, that is the ophthalmology clinics. Interestingly enough, glaucoma patients demonstrated far superior knowledge relative to the ophthalmic patients, which could only let us speculate about that of the general public. In fact, an examination of the general public and their glaucoma knowledge is the phase II of this project.

2. Name of the test should be mentioned here. Like regression, correlation etc

    a. **Response**: Type of statistical test was added to the abstract.

Introduction

3. Some explanation here as advanced age would be expected to have more knowledge? especially as it is a disease of older people usually

    a. **Response**: We thank the reviewer for raising such an important point. Explanation to this point was added to the discussion as not to congest the introduction. The following was added: "Interestingly, age was predominantly a negative predictor of glaucoma knowledge within the literature. A variety of factors could've influenced such relationship such as the low levels of health literacy in older individuals, neurological disease progression, or the inherent technological bias exhibited by younger participants able to fully utilize the internet to gain disease-related knowledge [34,44]."

4. Some explanation here as well

    a. **Response**: The statement was altered to showcase more clarity. The statement now reads: "Subsequently, those poor clinical presentations are associated with worse treatment outcomes as cases might be treated with more complex surgical options to halt the rapidly progressive and irreversible retinal damage [5,14]."

5. Difference between knowledge and awareness should be made clear

    a. **Response**: We thank the review for their keen comment. The following was added to the introduction: "The literature evaluating glaucoma knowledge within the Middle East and therefore its treatment implications, is scarce. In fact, an accurate estimation of knowledge is tricky to calculate due to the juxtaposition with the concept of awareness. It should be noted that awareness refers to the generalized or diffuse knowledge of a particular concept (e.g., have you heard of glaucoma?), while knowledge refers to any information that is factual in nature (e.g., prevalence, etiology, risk factors, etc.)[16]."

Methodology

6. self-administered questionnaire should be enough, also since this includes patients, an interview with questionnaire could have been a better option

   a. **Response:** We appreciate the reviewer's keen points. Patients were given the questionnaire and a team of specialized data collectors contacted the patients to ensure that they understood all items provided in the questionnaire. In fact, some older patients with severe visual disability needed the questionnaire to be read for them as they couldn't read it on their own.

7. Define type of glaucoma, how did you diagnose glaucoma.just mention e.g oct VF etc also exclude other types of glaucoma such as secondary causes

   a. **Response**: The target population in question were those with primary glaucoma. Since closed-angle glaucoma is a medical emergency, it is only logical that all recruited patients had chronic primary open angle glaucoma. Diagnostic modality and an expanded exclusion criteria were added. The new paragraph reads: "Patients were enrolled into two groups: The first group consisted of participants with a clinically proven diagnosis of primary glaucoma of at least 6 months and are on medications. Diagnosis of glaucoma was established on Optical Coherence Tomography. Patients with secondary glaucoma (e.g., pseudoexfoliation syndrome, pigment dispersion syndrome, neovascular glaucoma, inflammatory glaucoma, and medication-induced glaucoma) were excluded. The second group of participants consisted of those who were diagnosed with any vision hindering ophthalmic conditions with the exception of long-standing glaucoma. Such conditions include cataracts, diabetic retinopathy, macular degeneration, optic neuritis among others. Participants that were younger than 18 years of age, suffer from mental/psychiatric illnesses, and are unable to consent to participation in the survey were excluded from the study. Moreover, participants that failed to complete at least 80% of the questionnaire were also excluded."

8. vision hindering? define term..is it vision threating or just routine patients

   a. **Response**: The term was illuminated on with the use of examples.

9. exclusion and inclusion should be more specific..like glaucoma patients should not have other pathology etc

   a. **Response**: Refer to response to comment number 7

10. Non mutually exclusive? statement 28 through 30 not included in document so cant comment

   a. **Response**: The full questionnaire is now added to the revised submission.

11. any scoring system or just simple addition? was reverse coding utilised as well? as these were self administered questionnaires. without interview, some pariticipants might have filled in a hurry. Reverse coding might have hinted at that

   a. **Response**: Dear respected reviewer, the scoring was a simple addition of all correct items. The knowledge score wasn't even grouped into poor or good knowledge as such grouping would require validation and diagnostic analysis by ROC curves which outside the scope of this study. As for the reverse coding, as the reviewer knows, participants, especially those with vision ailments, are very sensitive to the number of questions in a questionnaire. Therefore, if reverse-coding was used, a higher rate of

participant attrition would occur. However, to combat the issue of participants finishing the questionnaire in a hurry, we implemented the following: 1) providing negative and positive statements throughout the questionnaire, 2) calculating the mean time to finish among participants which was about 8 minutes.

12. can be used for continuous data e.g mean between 2 groups, for categorical use chi square

   a. **Response**: The addition of (for categorical variables with more than two groups) next to the ANOVA test and the t-test was in reference to the INDEPDENT variable. Indeed, the ANOVA and t-test are tests of mean differences by which difference in a dependent continuous variable is tested among 2 independent groups (t-test) or 3+ independent groups (ANOVA). Therefore, to avoid this confusion that we may caused, the brackets were removed.

13. Please confirm this mean is taken from continuuous data, and not categorical data in spss, if it is categorical and we take mean it will be wrong. Just a heads up

   a. **Response**: All data presented as means ± SD were taken from continuous variables. Within the questionnaire number of medications, number of drops per day, and number of surgeries were asked.

14. mention which test is used here not just p value

   a. **Response**: The test used here was the chi squared test. The notion (X2(df) = X, p-value) denotes chi-square and was added accordingly.

15. elaborate on seizures?

   a. **Response**: Simply, we asked participants on what they thought are symptomatic complications of glaucoma. Options were reduced visual acuity, narrow visual field, vision loss, convulsive seizures, and symptoms of inflammation. Convulsive seizures refer to whole body seizures (grand mal seizures).

16. open or closed angle?these symptoms are usually in closed angle glaucoma , which might mean a difference in attitude towards adherence as it is more painful etc

   a. **Response**: Indeed, such symptoms denote a diagnosis of a closed-angle glaucoma. However, participants were asked these questions to test their knowledge of primary glaucoma including both closed and open angle.

17. mention which test is used here. it canot be t test

   a. **Response**: Indeed, each category was compared amongst the ophthalmic and glaucoma patients using the chi-square test. Mentioning of the chi-square test will be added in the footnote of the table.

18. which type of regression is used please mention here

   a. **Response**: Dear respected reviewer, univariate analysis within the context of this paragraph refers to either t-test or ANOVA and not regression (which is a form of multivariate analysis). Continuous variables such as knowledge score, symptom recognition, and adverse effect recognition were examined for difference between 2 groups using the t-test (e.g., biological sex, disease status, or family history of glaucoma), and the between 3 or more groups using the ANOVA test (e.g., residence,

education, or income). The notion of (t(df) = X, p-value) denotes the use of the student t-test and the notion of (F(df between groups, df within groups) = X, p-value) denotes the use of the ANOVA per APA guidelines. Both were added accordingly (when applicable).

19. linear regression cannot be used for categorical variables unless a dummy variable is made as a reference....or use logistic regression if the outcome is binary which again required reference variable...here the dummy or reference variable is not shown

a. **Response**: The reviewer is correct with their knowledge regarding linear regression; however, the linear regression model is found in table 5 and not table 4. Table 4 are mere univariate analysis consisting of t-test and ANOVA as described in the response above.

20. again family history i.e yes or no is categorical, either use dummy variable and show which is the reference variable

a. **Response**: Dear esteemed reviewer, indeed, family history is a categorical variable. The dummy variable in the model was no family history of glaucoma. The same applies for gender, smoking status (yes/no), and presence of comorbidities. Table 5, which contains the model is updated to contain the reference variables and reduce any confusion to readers.

21. Try to fit univariate and multivariate in 1 table....only show the significant results in the table to make it smaller. This allows easy comparison

a. **Response**: We appreciate the reviewer's concern with that regard. However, table 4 (table of univariate analysis) has 3 outcome measures (i.e., knowledge, symptom recognition, and adverse effect recognition scores), while table 5 (table of multivariate analysis) has only knowledge as its outcome variable. Therefore, combining the two tables would create a congested mess of numbers that might be harder to consume by the readers.

22. This part might be included introduction to avoid redundancy

a. **Response**: We thank the reviewer for raising such point. That part of the discussion was reorganized and place within the introduction.

23. how much % of glaucoma patients were aware? since the general population is aware 38.8% , it should be more than this theoretically

a. **Response**: Dear respected reviewer, indeed, knowledge of glaucoma should be higher among glaucoma patients than the general populace. However, the report which describes glaucoma knowledge among patients visiting Jordan University Hospital did not stratify knowledge per diagnosis. In fact, in their sample of 488, only 20 participants were diagnosed with glaucoma (Refer to: 10.4103/1319-4534.322618)

24. please clear the difference in introduction between awareness and knowledge as it seems to be mixed here/ confusing for the reader. Define it in objectives of study perhaps

a. **Response**: The following was added to the introduction to illuminate on the differences between awareness and knowledge: "The literature evaluating glaucoma knowledge within the Middle East and therefore its treatment implications, is scarce.

In fact, an accurate estimation of knowledge is tricky to calculate due to the juxtaposition with the concept of awareness. It should be noted that awareness refers to the generalized or diffuse knowledge of a particular concept (e.g., have you heard of glaucoma?), while knowledge refers to any information that is factual in nature (e.g., prevalence, etiology, risk factors, etc.)[16]."

25. in my opinion theres is little need for mentioning so many countries.Just local studies and few western studies should be enough perhaps

a. **Response**: We thank the reviewer for their keen concern. We believe that the inclusion of such studies serves two purposes. Firstly, it shows that variability between country and continents in terms of glaucoma knowledge and/or awareness. Secondly, it serves to provide future researchers and readers with a significant body of literature that they can explore, refer to, and even expand within the context of our results. If the reviewer strongly believes that a reduction should be made, then we are more than happy to comply.

26. just 2 or 3 references should be enough

a. **Response**: The number of references was reduced to 3 per the reviewer's advice.

27. Generally the discussion section is redundant in many places, confusing the reader.In my humble opinion it needs to be written coherently , in a stepwise manner and not repeat what has already been established in previous paragraphs. Also there seems to be a few grammatical mistakes. This can be solved by using software such as 'Grammarly'. Of course, Statistical analysis and results section first should be assured free of errors.

a. **Response**: Dear esteemed reviewer, all points raised with regards to the statistical analysis and results were addressed. Type of statistical testing was shown (resulted with expanded to show such a change) and every part of the analysis was described throughout our responses and changes within the manuscript. As for the discussion, it went through a grammatical check by a native English speaker and we cut at certain parts to avoid redundancy whenever applicable. The new discussion is now presented in the following themes: 1) Summary of findings, 2) Comparison with local literature, 3) Comparison with Western literature, 4) Illuminating on the reasons for variance between different countries/studies, 5) Factors associated with glaucoma knowledge, 6) Recommendations for better glaucoma knowledge and awareness, 7) Limitations.

28. Unclear ? please rephrase if possible

a. **Response**: The statement aims to stress on the fact that whatever educational intervention that will be given through concerned authorities, it must account that glaucoma have various types and might require different treatments for different risk groups. Therefore, an intervention must be clear, concise, and accommodating. To avoid redundancy, the statement was removed.
