# Supplementary material for: Predictors of glaucoma knowledge and its risk factors among Jordanian patients with primary open angle glaucoma at a tertiary teaching hospital: A cross-sectional survey
Source: PLoS One. 2023 May 18;18(5):e0285405. doi: 10.1371/journal.pone.0285405 (PMC10194903; doi:10.1371/journal.pone.0285405)
Supplement: Attachment — Submitted filename: Rebuttal Letter.docx [file pone.0285405.s004.docx]

Rebuttal Letter

On behalf of myself and all authors within this study, we thank the panel of reviewers for their extensive, shrewd, and informative comments. Any and all changes requested implied through your comments were considered and worked upon in order to strengthen the framework of our manuscript and render it suitable for publication in your respected journal. We would like to thank you for all of your efforts. The new manuscript file now has an expanded introduction, methodology, results, and discussion sections, all based on your comments. We hope that these expansions address your concerns and further validate our work. We immensely appreciate giving us the opportunity to revise our manuscript. We would like to thank the editorial team and the associated reviewers for their efforts. We hope that everyone is staying safe and well.

Editorial comments

1. Please ensure that your manuscript meets PLOS ONE's style requirements, including those for file naming.
   a. **Response**: The manuscript style was edited to match PLOS ONE’s style requirements.
2. Please provide additional details regarding participant consent. In the ethics statement in the Methods and online submission information, please ensure that you have specified (1) whether consent was informed and (2) what type you obtained (for instance, written or verbal, and if verbal, how it was documented and witnessed). If your study included minors, state whether you obtained consent from parents or guardians. If the need for consent was waived by the ethics committee, please include this information.
   a. **Response**: The ethical considerations section was updated to accommodate for the abovementioned concerns. The methodology section states that only participants 18 years and older were recruited; therefore, no minors were included in the study.
3. You indicated that you had ethical approval for your study. Please clarify whether minors (participants under the age of 18 years) were included in this study. If yes, in your Methods section, please ensure you have also stated whether you obtained consent from parents or guardians of the minors included in the study or whether the research ethics committee or IRB specifically waived the need for their consent.
   a. **Response**: The ethical considerations section was updated to accommodate for the abovementioned concerns. The methodology section states that only participants 18 years and older were recruited; therefore, no minors were included in the study.
4. Please review your reference list to ensure that it is complete and correct. If you have cited papers that have been retracted, please include the rationale for doing so in the manuscript text, or remove these references and replace them with relevant current references. Any changes to the reference list should be mentioned in the rebuttal letter that accompanies your revised manuscript. If you need to cite a retracted article, indicate the article’s retracted status in the References list and also include a citation and full reference for the retraction notice.
   a. **Response**: The reference list was checked and the any added references resulted from dealing with reviewers’ comments were noted at in the rebuttal letter.

Reviewer comments

**Abstract**

1. Why compare to ophthalmic patients? this demography is expected to demonstrate some knowledge a eye patients. so maybe the public at large would be a better option especially considering the use of self administered questionnaire
   a. **Response**: Dear respected reviewer, the data collection was designed in such a fashion in order to concentrate efforts into the recruitment of patients within one space, that is the ophthalmology clinics. Interestingly enough, glaucoma patients demonstrated far superior knowledge relative to the ophthalmic patients, which could only let us speculate about that of the general public. In fact, an examination of the general public and their glaucoma knowledge is the phase II of this project.
2. Name of the test should be mentioned here. Like regression, correlation etc
   a. **Response**: Type of statistical test was added to the abstract.

**Introduction**

3. Some explanation here as advanced age would be expected to have more knowledge? especially as it is a disease of older people usually
   a. **Response**: We thank the reviewer for raising such an important point. Explanation to this point was added to the discussion as not to congest the introduction. The following was added: “Interestingly, age was predominantly a negative predictor of glaucoma knowledge within the literature. A variety of factors could’ve influenced such relationship such as the low levels of health literacy in older individuals, neurological disease progression, or the inherent technological bias exhibited by younger participants able to fully utilize the internet to gain disease-related knowledge [34,44].”
4. Some explanation here as well
   a. **Response**: The statement was altered to showcase more clarity. The statement now reads: “Subsequently, those poor clinical presentations are associated with worse treatment outcomes as cases might be treated with more complex surgical options to halt the rapidly progressive and irreversible retinal damage [5,14].”
5. Difference between knowledge and awareness should be made clear
   a. **Response**: We thank the review for their keen comment. The following was added to the introduction: “The literature evaluating glaucoma knowledge within the Middle East and therefore its treatment implications, is scarce. In fact, an accurate estimation of knowledge is tricky to calculate due to the juxtaposition with the concept of awareness. It should be noted that awareness refers to the generalized or diffuse knowledge of a particular concept (e.g., have you heard of glaucoma?), while knowledge refers to any information that is factual in nature (e.g., prevalence, etiology, risk factors, etc.)[16].”

**Methodology**

6. self-administered questionnaire should be enough, also since this includes patients, an interview with questionnaire could have been a better option
   a. **Response:** We appreciate the reviewer’s keen points. Patients were given the questionnaire and a team of specialized data collectors contacted the patients to ensure that they understood all items provided in the questionnaire. In fact, some older patients with severe visual disability needed the questionnaire to be read for them as they couldn’t read it on their own.
7. Define type of glaucoma, how did you diagnose glaucoma.just mention e.g oct VF etc also exclude other types of glaucoma such as secondary causes
   a. **Response**: The target population in question were those with primary glaucoma. Since closed-angle glaucoma is a medical emergency, it is only logical that all recruited patients had chronic primary open angle glaucoma. Diagnostic modality and an expanded exclusion criteria were added. The new paragraph reads: “Patients were enrolled into two groups: The first group consisted of participants with a clinically proven diagnosis of primary glaucoma of at least 6 months and are on medications. Diagnosis of glaucoma was established on Optical Coherence Tomography. Patients with secondary glaucoma (e.g., pseudoexfoliation syndrome, pigment dispersion syndrome, neovascular glaucoma, inflammatory glaucoma, and medication-induced glaucoma) were excluded. The second group of participants consisted of those who were diagnosed with any vision hindering ophthalmic conditions with the exception of long-standing glaucoma. Such conditions include cataracts, diabetic retinopathy, macular degeneration, optic neuritis among others. Participants that were younger than 18 years of age, suffer from mental/psychiatric illnesses, and are unable to consent to participation in the survey were excluded from the study. Moreover, participants that failed to complete at least 80% of the questionnaire were also excluded.”
8. vision hindering? define term..is it vision threating or just routine patients
   a. **Response**: The term was illuminated on with the use of examples.
9. exclusion and inclusion should be more specific..like glaucoma patients should not have other pathology etc
   a. **Response**: Refer to response to comment number 7
10. Non mutually exclusive? statement 28 through 30 not included in document so cant comment
    a. **Response**: The full questionnaire is now added to the revised submission.
11. any scoring system or just simple addition? was reverse coding utilised as well? as these were self administered questionnaires. without interview, some pariticipants might have filled in a hurry. Reverse coding might have hinted at that
    a. **Response**: Dear respected reviewer, the scoring was a simple addition of all correct items. The knowledge score wasn’t even grouped into poor or good knowledge as such grouping would require validation and diagnostic analysis by ROC curves which outside the scope of this study. As for the reverse coding, as the reviewer knows, participants, especially those with vision ailments, are very sensitive to the number of questions in a questionnaire. Therefore, if reverse-coding was used, a higher rate of participant attrition would occur. However, to combat the issue of participants finishing the questionnaire in a hurry, we implemented the following: 1) providing negative and positive statements throughout the questionnaire, 2) calculating the mean time to finish among participants which was about 8 minutes.
12. can be used for continuous data e.g mean between 2 groups, for categorical use chi square
    a. **Response**: The addition of (for categorical variables with more than two groups) next to the ANOVA test and the t-test was in reference to the INDEPDENT variable. Indeed, the ANOVA and t-test are tests of mean differences by which difference in a dependent continuous variable is tested among 2 independent groups (t-test) or 3+ independent groups (ANOVA). Therefore, to avoid this confusion that we may caused, the brackets were removed.
13. Please confirm this mean is taken from continuuous data, and not categorical data in spss, if it is categorical and we take mean it will be wrong. Just a heads up
    a. **Response**: All data presented as means ± SD were taken from continuous variables. Within the questionnaire number of medications, number of drops per day, and number of surgeries were asked.
14. mention which test is used here not just p value
    a. **Response**: The test used here was the chi squared test. The notion (X2(df) = X, p-value) denotes chi-square and was added accordingly.
15. elaborate on seizures?
    a. **Response**: Simply, we asked participants on what they thought are symptomatic complications of glaucoma. Options were reduced visual acuity, narrow visual field, vision loss, convulsive seizures, and symptoms of inflammation. Convulsive seizures refer to whole body seizures (grand mal seizures).
16. open or closed angle?these symptoms are usually in closed angle glaucoma , which might mean a difference in attitude towards adherence as it is more painful etc
    a. **Response**: Indeed, such symptoms denote a diagnosis of a closed-angle glaucoma. However, participants were asked these questions to test their knowledge of primary glaucoma including both closed and open angle.
17. mention which test is used here. it canot be t test
    a. **Response**: Indeed, each category was compared amongst the ophthalmic and glaucoma patients using the chi-square test. Mentioning of the chi-square test will be added in the footnote of the table.
18. which type of regression is used please mention here
    a. **Response**: Dear respected reviewer, univariate analysis within the context of this paragraph refers to either t-test or ANOVA and not regression (which is a form of multivariate analysis). Continuous variables such as knowledge score, symptom recognition, and adverse effect recognition were examined for difference between 2 groups using the t-test (e.g., biological sex, disease status, or family history of glaucoma), and the between 3 or more groups using the ANOVA test (e.g., residence, education, or income). The notion of (t(df) = X, p-value) denotes the use of the student t-test and the notion of (F(df between groups, df within groups) = X, p-value) denotes the use of the ANOVA per APA guidelines. Both were added accordingly (when applicable).
19. linear regression cannot be used for categorical variables unless a dummy variable is made as a reference....or use logistic regression if the outcome is binary which again required reference variable...here the dummy or reference variable is not shown
    a. **Response**: The reviewer is correct with their knowledge regarding linear regression; however, the linear regression model is found in table 5 and not table 4. Table 4 are mere univariate analysis consisting of t-test and ANOVA as described in the response above.
20. again family history i.e yes or no is categorical, either use dummy variable and show which is the reference variable
    a. **Response**: Dear esteemed reviewer, indeed, family history is a categorical variable. The dummy variable in the model was no family history of glaucoma. The same applies for gender, smoking status (yes/no), and presence of comorbidities. Table 5, which contains the model is updated to contain the reference variables and reduce any confusion to readers.
21. Try to fit univariate and multivariate in 1 table....only show the significant results in the table to make it smaller. This allows easy comparison
    a. **Response**: We appreciate the reviewer’s concern with that regard. However, table 4 (table of univariate analysis) has 3 outcome measures (i.e., knowledge, symptom recognition, and adverse effect recognition scores), while table 5 (table of multivariate analysis) has only knowledge as its outcome variable. Therefore, combining the two tables would create a congested mess of numbers that might be harder to consume by the readers.
22. This part might be included introduction to avoid redundancy
    a. **Response**: We thank the reviewer for raising such point. That part of the discussion was reorganized and place within the introduction.
23. how much % of glaucoma patients were aware? since the general population is aware 38.8% , it should be more than this theoretically
    a. **Response**: Dear respected reviewer, indeed, knowledge of glaucoma should be higher among glaucoma patients than the general populace. However, the report which describes glaucoma knowledge among patients visiting Jordan University Hospital did not stratify knowledge per diagnosis. In fact, in their sample of 488, only 20 participants were diagnosed with glaucoma (Refer to: 10.4103/1319-4534.322618)
24. please clear the difference in introduction between awareness and knowledge as it seems to be mixed here/ confusing for the reader. Define it in objectives of study perhaps
    a. **Response**: The following was added to the introduction to illuminate on the differences between awareness and knowledge: “The literature evaluating glaucoma knowledge within the Middle East and therefore its treatment implications, is scarce. In fact, an accurate estimation of knowledge is tricky to calculate due to the juxtaposition with the concept of awareness. It should be noted that awareness refers to the generalized or diffuse knowledge of a particular concept (e.g., have you heard of glaucoma?), while knowledge refers to any information that is factual in nature (e.g., prevalence, etiology, risk factors, etc.)[16].”
25. in my opinion theres is little need for mentioning so many countries.Just local studies and few western studies should be enough perhaps
    a. **Response**: We thank the reviewer for their keen concern. We believe that the inclusion of such studies serves two purposes. Firstly, it shows that variability between country and continents in terms of glaucoma knowledge and/or awareness. Secondly, it serves to provide future researchers and readers with a significant body of literature that they can explore, refer to, and even expand within the context of our results. If the reviewer strongly believes that a reduction should be made, then we are more than happy to comply.
26. just 2 or 3 references should be enough
    a. **Response**: The number of references was reduced to 3 per the reviewer’s advice.
27. Generally the discussion section is redundant in many places, confusing the reader.In my humble opinion it needs to be written coherently , in a stepwise manner and not repeat what has already been established in previous paragraphs. Also there seems to be a few grammatical mistakes. This can be solved by using software such as 'Grammarly'. Of course, Statistical analysis and results section first should be assured free of errors.
    a. **Response**: Dear esteemed reviewer, all points raised with regards to the statistical analysis and results were addressed. Type of statistical testing was shown (resulted with expanded to show such a change) and every part of the analysis was described throughout our responses and changes within the manuscript. As for the discussion, it went through a grammatical check by a native English speaker and we cut at certain parts to avoid redundancy whenever applicable. The new discussion is now presented in the following themes: 1) Summary of findings, 2) Comparison with local literature, 3) Comparison with Western literature, 4) Illuminating on the reasons for variance between different countries/studies, 5) Factors associated with glaucoma knowledge, 6) Recommendations for better glaucoma knowledge and awareness, 7) Limitations.
28. Unclear ? please rephrase if possible
    a. **Response**: The statement aims to stress on the fact that whatever educational intervention that will be given through concerned authorities, it must account that glaucoma have various types and might require different treatments for different risk groups. Therefore, an intervention must be clear, concise, and accommodating. To avoid redundancy, the statement was removed.
