# Supplementary material for: Predictors of glaucoma knowledge and its risk factors among Jordanian patients with primary open angle glaucoma at a tertiary teaching hospital: A cross-sectional survey
Source: PLoS One. 2023 May 18;18(5):e0285405. doi: 10.1371/journal.pone.0285405 (PMC10194903; doi:10.1371/journal.pone.0285405)
Supplement: Attachment — Submitted filename: Predictors of glaucoma knowledge and its risk factors among Jordanian patients with primary open angle glaucoma at a tertiary teaching hospital.docx [file pone.0285405.s006.docx]

Predictors of glaucoma knowledge and its risk factors among Jordanian patients with primary open angle glaucoma at a tertiary teaching hospital: a cross-sectional survey

ABSTRACT

The purpose of the study is mentioned to be to assess the glaucoma knowledge and adherence to care seeking behaviors. However, there is no mention of adherence related data included in the study.

INTRODUCTION

Paragraph 1-

- ‘due to its asymptomatic nature, lack of awareness of risk, and low levels of referral, the incidence of glaucoma is rising within the population’

Incidence refers to number of new cases in a certain period. The factors you mentioned may contribute to a rise of glaucoma blindness but not incidence of glaucoma.

MATERIALS AND METHODS

- What is the explanation to use these two groups as study method?
- Group 1 consisted of POAG and such patients are expected to be 40 years of age or above. However, the age limit included in the study is above 18 years of age. It appears those with Juvenile Open Angle Glaucoma may have been included. It is better to be clear about this.
- How could OCT alone establish the diagnosis of glaucoma?
- It is mentioned simple random sampling is utilized and every 4^th^ patient was recruited. There is inadequate information to explain why this method was used. For example, What was the sample size? Did you have a sampling frame?

RESULTS

- There is no operational definition given to identify the level of knowledge of participants. You mentioned in your discussion that your study demonstrated glaucoma patients are more knowledgeable about glaucoma than their non-glaucoma counterparts. Where is the cutoff score to say one is knowledgeable?
- There are some factors that were found to be positive predictors of higher glaucoma knowledge scores. However, it is not clear whether those factors were analyzed for the entire sample (two groups together) or for the glaucoma patients (group 1) alone. Those predictor factors may not be associated for the two groups if analyzed separately.

DISCUSSION

- You included many literature references from Asia to Africa that mentioned the level of awareness of glaucoma but you have not mentioned the level of awareness you found amongst your participants.
