# Supplementary material for: Predictors of glaucoma knowledge and its risk factors among Jordanian patients with primary open angle glaucoma at a tertiary teaching hospital: A cross-sectional survey
Source: PLoS One. 2023 May 18;18(5):e0285405. doi: 10.1371/journal.pone.0285405 (PMC10194903; doi:10.1371/journal.pone.0285405)
Supplement: Attachment — Submitted filename: Rebuttal Letter.docx [file pone.0285405.s007.docx]

**Rebuttal Letter**

On behalf of myself and all authors within this study, we thank the panel of reviewers for their extensive, shrewd, and informative comments. Any and all changes requested implied through your comments were considered and worked upon in order to strengthen the framework of our manuscript and render it suitable for publication in your respected journal. We would like to thank you for all of your efforts. We edited the manuscript based on your comments and illuminated on areas that were condemned to be vague or misunderstand. We hope that these edits address your concerns and further validate our work. We immensely appreciate giving us the opportunity to revise our manuscript. We would like to thank the editorial team and the associated reviewers for their efforts. We hope that everyone is staying safe and well.

**Reviewer 3**

Reviewer #3: The authors describe in this report the predictors of glaucoma knowledge and its risk factors among a group of Jordanian patients attending a tertiary teaching hospital in Jordan. The manuscript is well written and detailed and I congratulate the authors on their extensive work.

Abstract:

1. In the conclusion, when you refer to ophthalmic patients, it is better to describe both glaucoma and non-glaucoma. E.g. We have demonstrated that both glaucoma and non-glaucoma ophthalmic patients display mediocre levels of glaucoma knowledge.

**RESPONSE**: We thank the respected reviewer for their suggestion. The abovementioned edit was conduct accordingly.

2. Also avoid the use of gross terms such as mediocre.

**RESPONSE**: We thank the reviewer for their suggestions. Words such as “mediocre”, “underwhelming”, and “hindering” were replaced by “average”, “limiting”, and “defective”, respectively.

Introduction:

3. The authors state: “Vision loss associated with glaucoma can be prevented with early diagnosis and treatment; however, due to its asymptomatic nature, lack of awareness of risk, and low levels of referral, the incidence of glaucoma is rising within the population [4].” The above statement is confusing. It may be better to state that “the incidence/prevalence of glaucoma is under-reported.”

**RESPONSE**: We thank the respected reviewer for their suggestion. The abovementioned edit was conduct accordingly.

Methods:

4. The word hindering should be substituted with a clearer and simpler term.

**RESPONSE**: We thank the reviewer for their suggestions. Words such as “mediocre”, “underwhelming”, and “hindering” were replaced by “average”, “limiting”, and “defective”, respectively.

5. The authors state: “Such conditions include cataracts, diabetic retinopathy, macular degeneration, and optic neuritis among others.”

Please avoid using “among other”, be specific. Mention the common causes or use causes as they were presented in the results section.

**RESPONSE**: The statement was completed and altered to the following: “Such conditions include cataracts, diabetic retinopathy, macular degeneration, optic neuritis/atrophy, cerebrovascular disease, and hereditary retinal disorders.”

6. In instrument development, the Arabic questionnaire (appendix S2 file) is missing question 11. I am sure this has been attended to, but the authors need to make sure that data have not been compromised by wrong or miss-arrangement order of the questions.

**RESPONSE**: We thank the reviewer for their keen observation. The S2 arabic questionnaire file is now fixed. The item was not missing but rather the numbering was shifted and started from 12 instead of 11. The fidelity of the analysis and order of questions within the analysis sheet was rechecked accordingly.

7. Also, 1 point was given to each correct answer for the first 27 questions, and then added together. The authors assume equal weight of each question in the first 27 questions, but this might not be the case. Please clarify why you think all the questions have equal weights?

**RESPONSE**: We thank the reviewer for pointing out this particular conundrum. According to relevant literature not only limited to glaucoma, the concept of a “reliable self-reported knowledge” instrument might not be achievable in practice. This is simply due to the lack of a “gold standard” for comparison. What constitutes a “knowledgeable” patient/person varies from one physician to another.

Interestingly, and in line with literature trying to produce glaucoma knowledge scales (e.g., National Eye Health Education Program, Hoevenaars et al., Gray et al.), every single one of them were scored with 1 point given to every correct included statement (DOI: 10.1186/s12886-022-02322-0; 10.4274/tjo.26576; 10.1080/08820538.2016.1247180). This brings us back to the point raised above, why would statement X hold more weight than statement Y but not statement Z. In this case, a true weighting of different statements can only be achieved by expert consensus of a large sample of field experts.

While our scoring is in line with convenient pattern of the literature of similar glaucoma knowledge questionnaires, we did statistically examine the differences within each statement of the 27 among glaucoma vs. non-glaucoma patients (Refer to table 2).

8. Once again, 1 point is given for the correct answer for questions with a Likert scale, but can the authors explain what is the correct answer if one subject answered” strongly agree” and the another subject answered “ agree”. I assume that categorization into 3 categories eliminated this issue. Please clarify/confirm.

**RESPONSE**: We thank the reviewer for raising such a point. The 5-point likert scale was condensed as follows: (strongly agree + agree as agree), (neutral = neutral), (strongly disagree + disagree as disagree). We have included that in our statistical analysis part of the methodology.

Discussion

9. The authors state: “Our study demonstrated that glaucoma knowledge among Jordanian ophthalmic patients is rather underwhelming”.

Once again, avoid the use of terms such as underwhelming and use only plain scientific terms.

**RESPONSE**: We thank the reviewer for their suggestions. Words such as “mediocre”, “underwhelming”, and “hindering” were replaced by “average”, “limiting”, and “defective”, respectively.

10. The discussion section is rather long and readers can feel bored. It can be improved by shortening the text. Many of the comparisons with other studies can be summarized in a table as an example.

**RESPONSE**: We thank the reviewer for their comment. Indeed, the discussion is pretty verbose. While having a table to discuss differences in study findings may be more suitable for a systematic review, we have reduced the discussion from 1750 words to 1289 words. We have removed any and all redundancies. Moreover, any information that can be extracted from the original references were removed as to avoid repeating large sums of information.

**Reviewer 4**

ABSTRACT

The purpose of the study is mentioned to be to assess the glaucoma knowledge and adherence to care seeking behaviors. However, there is no mention of adherence related data included in the study.

**RESPONSE**: We thank the reviewer for raising this point, the aim/objective part of the abstract now reads: “To assess and compare glaucoma knowledge between Jordanian patients with glaucoma and non-glaucoma ophthalmic patients.”.

INTRODUCTION

Paragraph 1- ‘due to its asymptomatic nature, lack of awareness of risk, and low levels of referral, the incidence of glaucoma is rising within the population’. Incidence refers to number of new cases in a certain period. The factors you mentioned may contribute to a rise of glaucoma blindness but not incidence of glaucoma.

**RESPONSE**: We thank the reviewer for their suggestion. Upon reviewing the original reference, those factors were associated with the increase in prevalence and not incidence. The statement was altered accordingly.

MATERIALS AND METHODS

What is the explanation to use these two groups as study method?

**RESPONSE**: The study adopts a cross-sectional design in which two groups (one with glaucoma vs. another without glaucoma) were examined for glaucoma-related differences. The data collection was designed in such a fashion in order to concentrate efforts into the recruitment of patients within one space, that is the ophthalmology clinics. Interestingly enough, glaucoma patients demonstrated far superior knowledge relative to the non-glaucoma ophthalmic patients, which could only let us speculate about that of the general public. In fact, an examination of the general public and their glaucoma knowledge is the phase II of this project.

Group 1 consisted of POAG and such patients are expected to be 40 years of age or above. However, the age limit included in the study is above 18 years of age. It appears those with Juvenile Open Angle Glaucoma may have been included. It is better to be clear about this.

**RESPONSE**: We thank the reviewer for their expert comment. However, no cases of Juvenile Open Angle Glaucoma were included. The 18 years age limit is concerned with the ethical considerations of the study and must be routinely included. If we are to include patients from under 18 years of age, a different consent form and ethical considerations will be applied. Therefore, the 18 years of age limit is only arbitrary and serves as a complementary statement to the ethical consideration section.

How could OCT alone establish the diagnosis of glaucoma?

**RESPONSE**: We appreciate the esteemed reviewer’s concern. Recruited glaucoma were not diagnosis based on OCT alone but rather the usual mix of optic nerve examination, clinical symptoms, perimetry, gonioscopy, and OCT/RNFL visualization. For the sake of ease, we included only the objective imaging modality. However, for the sake of completion, we have updated the statement to the following: “Diagnosis of glaucoma was established using slit lamp examination, visual field testing, gonioscopy, and imaging (i.e., Optical Coherence Tomography)”.

It is mentioned simple random sampling is utilized and every 4^th^ patient was recruited. There is inadequate information to explain why this method was used. For example, What was the sample size? Did you have a sampling frame?

**RESPONSE**: Dear reviewer, random sample is the best method to yield samples that do not have skewed characteristics. While recruiting such a sample may not be always the case due to methodological errors in data collection, random sampling is superior to convenient (a.k.a non-random) sampling. Moreover, when calculating sample sizes, the resultant minimum recommended sample size is expected to be recruited through random sampling.

With regards to our calculated sample size, we did not calculate a population-based sample size. There is no reliable data about the number of glaucoma patients in Jordan, therefore, we are sampling from an unknown population. However, we have conducted a test-oriented sample size calculation. The reviewer should be rest assured that to conduct power chi-square, t-test/ANOVA, and regression, a minimum of 80, 220 (110 per group), and 172 participants are required (G*Power, alpha error 5%, power 95%). Our sample satisfies the aforementioned requirements.

RESULTS

There is no operational definition given to identify the level of knowledge of participants. You mentioned in your discussion that your study demonstrated glaucoma patients are more knowledgeable about glaucoma than their non-glaucoma counterparts. Where is the cutoff score to say one is knowledgeable?

**RESPONSE**: We thank the reviewer for pointing out this particular issue. According to relevant literature not only limited to glaucoma, the concept of a “reliable self-reported knowledge” instrument might not be achievable in practice. This is simply due to the lack of a “gold standard” for comparison. What constitutes a “knowledgeable” patient/person varies from one physician to another.

In fact, the literature involving glaucoma knowledge questionnaire doesn’t have any cut-off points (DOI: 10.1186/s12886-022-02322-0; 10.4274/tjo.26576; 10.1080/08820538.2016.1247180; among others). Even when cut-off points are made, they are often arbitrary either based on quartiles or the median value for the entire score.

Not to deviate, our statement refers to the fact that glaucoma patients have “relatively” more knowledge than their non-glaucoma counterparts; a finding proven through statistical analysis (i.e., higher knowledge scores for the earlier group). The statement was altered to include “relatively” to avoid misconceptions.

There are some factors that were found to be positive predictors of higher glaucoma knowledge scores. However, it is not clear whether those factors were analyzed for the entire sample (two groups together) or for the glaucoma patients (group 1) alone. Those predictor factors may not be associated for the two groups if analyzed separately.

**RESPONSE**: We thank the esteemed reviewer for raising such an important point. The analysis was done for both groups combined. Unfortunately, this is a product of limitation as regression analysis requires large sample sizes to be statistically relevant. Our G*Power sample size calculation requires at least 170 participants for a linear regression to be relevant. This is only applicable to the sample as a whole and not its glaucoma non-glaucoma subgroups. Therefore, we only edited the table’s title to “Predictors of knowledge score for the studied sample (n = 256)” to avoid confusion and increase clarity. This limitation was accordingly added to the limitations section.

DISCUSSION

You included many literature references from Asia to Africa that mentioned the level of awareness of glaucoma but you have not mentioned the level of awareness you found amongst your participants.

**RESPONSE**: We understand the esteemed reviewer’s concern. Within our definitions, we state “It should be noted that awareness refers to the generalized or diffuse knowledge of a particular concept (e.g., have you heard of glaucoma?), while knowledge refers to any information that is factual in nature (e.g., prevalence, etiology, risk factors, etc.)[16].”. Knowledge was the primary aim of this questionnaire and study and not awareness. We did not have a “have you heard of glaucoma” or “have you heard of glaucoma’s treatments” kind of questions (which measure superficial knowledge = awareness) but rather had statements that measure deep knowledge which is referred to as knowledge.

However, our inclusion of studies that only measure awareness, such as those originating from Asia to Africa, is to provide a comprehensive view of all the literature that attempted to measure awareness or knowledge of glaucoma. What’s even more peculiar is that the use of awareness and knowledge is often interchangeable within the literature; an issue well documented across many topics and disciplines that aim to measure knowledge (DOI: 10.3389/fpubh.2017.00194).

**Reviewer 1**

(Comments highlighted on PDF)

1. Grammatical issues were fixed per the reviewer’s comments

2. The tables were altered per the reviewer’s comments

3. In response to “Were all these variables significant in univariate analysis? Only then should be added in multivariate model”.

**RESPONSE**: We fully understand the reviewer’s concern. When creating models of prediction, such as that of regression, the inclusion of variables can be made through a variety of methods. These methods include 1) significant variables on univariate analysis (which was pointed out by the respected reviewer, 2) variables with p-values less than 0.10 in the univariate analysis, 3) “a priori” inclusion of such variables during the pre-research conduction stage, or 4) variables that demonstrated association with the dependent outcomes (i.e., knowledge) throughout the literature. A mix of the aforementioned methods is feasible as long as it produces models that are free of collinearity and have the highest explanatory power. Our presented model had no collinearity based on the Durbin-Watson statistic and had the highest R2 value out of all the other attempted models.

4. The limitations section was edited per the reviewer’s comments
