# Supplementary material for: Predictors of glaucoma knowledge and its risk factors among Jordanian patients with primary open angle glaucoma at a tertiary teaching hospital: A cross-sectional survey
Source: PLoS One. 2023 May 18;18(5):e0285405. doi: 10.1371/journal.pone.0285405 (PMC10194903; doi:10.1371/journal.pone.0285405)
Supplement: Peer review history

# Peer review: Decision Letter 0

**DOI:** 10.1371/journal.pone.0285405.r001

**Authors:** Bharat Gurnani

17 Jan 2023

PONE-D-22-22392Predictors of glaucoma knowledge and its risk factors among Jordanian patients with primary open angle glaucoma at a tertiary teaching hospital: a cross-sectional surveyPLOS ONE

Dear Dr. Abdallah Al-Ani

Thank you for submitting your manuscript to PLOS ONE. After careful consideration, we feel that it has merit but does not fully meet PLOS ONE’s publication criteria as it currently stands. Therefore, we invite you to submit a revised version of the manuscript that addresses the points raised during the review process.

Please submit your revised manuscript by Mar 03 2023 11:59PM. If you will need more time than this to complete your revisions, please reply to this message or contact the journal office at plosone@plos.org. When you're ready to submit your revision, log on to https://www.editorialmanager.com/pone/ and select the 'Submissions Needing Revision' folder to locate your manuscript file.

Please include the following items when submitting your revised manuscript:

- A rebuttal letter that responds to each point raised by the academic editor and reviewer(s). You should upload this letter as a separate file labeled 'Response to Reviewers'.
- A marked-up copy of your manuscript that highlights changes made to the original version. You should upload this as a separate file labeled 'Revised Manuscript with Track Changes'.
- An unmarked version of your revised paper without tracked changes. You should upload this as a separate file labeled 'Manuscript'.

If you would like to make changes to your financial disclosure, please include your updated statement in your cover letter. Guidelines for resubmitting your figure files are available below the reviewer comments at the end of this letter.

If applicable, we recommend that you deposit your laboratory protocols in protocols.io to enhance the reproducibility of your results. Protocols.io assigns your protocol its own identifier (DOI) so that it can be cited independently in the future. For instructions see: https://journals.plos.org/plosone/s/submission-guidelines#loc-laboratory-protocols. Additionally, PLOS ONE offers an option for publishing peer-reviewed Lab Protocol articles, which describe protocols hosted on protocols.io. Read more information on sharing protocols at https://plos.org/protocols?utm_medium=editorial-email&utm_source=authorletters&utm_campaign=protocols.

We look forward to receiving your revised manuscript.

Kind regards,

Bharat Gurnani

Academic Editor

PLOS ONE

Journal Requirements:

When submitting your revision, we need you to address these additional requirements.

1. Please ensure that your manuscript meets PLOS ONE's style requirements, including those for file naming. The PLOS ONE style templates can be found at

https://journals.plos.org/plosone/s/file?id=wjVg/PLOSOne_formatting_sample_main_body.pdf and

https://journals.plos.org/plosone/s/file?id=ba62/PLOSOne_formatting_sample_title_authors_affiliations.pdf

2. Please provide additional details regarding participant consent. In the ethics statement in the Methods and online submission information, please ensure that you have specified (1) whether consent was informed and (2) what type you obtained (for instance, written or verbal, and if verbal, how it was documented and witnessed). If your study included minors, state whether you obtained consent from parents or guardians. If the need for consent was waived by the ethics committee, please include this information.

If you are reporting a retrospective study of medical records or archived samples, please ensure that you have discussed whether all data were fully anonymized before you accessed them and/or whether the IRB or ethics committee waived the requirement for informed consent. If patients provided informed written consent to have data from their medical records used in research, please include this information.

3. You indicated that you had ethical approval for your study. Please clarify whether minors (participants under the age of 18 years) were included in this study. If yes, in your Methods section, please ensure you have also stated whether you obtained consent from parents or guardians of the minors included in the study or whether the research ethics committee or IRB specifically waived the need for their consent.

4. Please review your reference list to ensure that it is complete and correct. If you have cited papers that have been retracted, please include the rationale for doing so in the manuscript text, or remove these references and replace them with relevant current references. Any changes to the reference list should be mentioned in the rebuttal letter that accompanies your revised manuscript. If you need to cite a retracted article, indicate the article’s retracted status in the References list and also include a citation and full reference for the retraction notice.

Additional Editor Comments (if provided):

The authors are required to answer the reviewer comments and submit the revised file in stipulated time period

[Note: HTML markup is below. Please do not edit.]

Reviewers' comments:

Reviewer's Responses to Questions

**Comments to the Author**

1. Is the manuscript technically sound, and do the data support the conclusions?

The manuscript must describe a technically sound piece of scientific research with data that supports the conclusions. Experiments must have been conducted rigorously, with appropriate controls, replication, and sample sizes. The conclusions must be drawn appropriately based on the data presented. 

Reviewer #1: Yes

Reviewer #2: Yes

**********

2. Has the statistical analysis been performed appropriately and rigorously? 

Reviewer #1: No

Reviewer #2: Yes

**********

3. Have the authors made all data underlying the findings in their manuscript fully available?

The PLOS Data policy requires authors to make all data underlying the findings described in their manuscript fully available without restriction, with rare exception (please refer to the Data Availability Statement in the manuscript PDF file). The data should be provided as part of the manuscript or its supporting information, or deposited to a public repository. For example, in addition to summary statistics, the data points behind means, medians and variance measures should be available. If there are restrictions on publicly sharing data—e.g. participant privacy or use of data from a third party—those must be specified.

Reviewer #1: Yes

Reviewer #2: Yes

**********

4. Is the manuscript presented in an intelligible fashion and written in standard English?

PLOS ONE does not copyedit accepted manuscripts, so the language in submitted articles must be clear, correct, and unambiguous. Any typographical or grammatical errors should be corrected at revision, so please note any specific errors here.

Reviewer #1: No

Reviewer #2: Yes

**********

5. Review Comments to the Author

Please use the space provided to explain your answers to the questions above. You may also include additional comments for the author, including concerns about dual publication, research ethics, or publication ethics. (Please upload your review as an attachment if it exceeds 20,000 characters)

Reviewer #1: Very comprehensive research performed on a topic that still has many unexplored aspects in the literature. The submission will definitely be helpful to answer many questions related to glaucoma knowledge and awareness in Asian population

Reviewer #2: The manuscript is well-written and the paper seems to be an interesting one which looks into glaucoma knowledge among patients with glaucoma and other ophthalmic conditions in Jordan. The study demonstrated the lack of knowledge among the people for one of the leading cause of blindness in the world and also reiterated the need for awareness to increase the knowledge and to decrease the disease burden.

**********

6. PLOS authors have the option to publish the peer review history of their article (what does this mean?). If published, this will include your full peer review and any attached files.

If you choose “no”, your identity will remain anonymous but your review may still be made public.

**Do you want your identity to be public for this peer review?** For information about this choice, including consent withdrawal, please see our Privacy Policy.

Reviewer #1: **Yes: **Mr. Mahmood Ali

Reviewer #2: No

**********

[NOTE: If reviewer comments were submitted as an attachment file, they will be attached to this email and accessible via the submission site. Please log into your account, locate the manuscript record, and check for the action link "View Attachments". If this link does not appear, there are no attachment files.]

While revising your submission, please upload your figure files to the Preflight Analysis and Conversion Engine (PACE) digital diagnostic tool, https://pacev2.apexcovantage.com/. PACE helps ensure that figures meet PLOS requirements. To use PACE, you must first register as a user. Registration is free. Then, login and navigate to the UPLOAD tab, where you will find detailed instructions on how to use the tool. If you encounter any issues or have any questions when using PACE, please email PLOS at figures@plos.org. Please note that Supporting Information files do not need this step.

Attachment Submitted filename: PONE-D-22-22392 review.pdf Click here for additional data file.

# Author comment: Author response to Decision Letter 0

**DOI:** 10.1371/journal.pone.0285405.r002

30 Jan 2023

Rebuttal Letter

On behalf of myself and all authors within this study, we thank the panel of reviewers for their extensive, shrewd, and informative comments. Any and all changes requested implied through your comments were considered and worked upon in order to strengthen the framework of our manuscript and render it suitable for publication in your respected journal. We would like to thank you for all of your efforts. The new manuscript file now has an expanded introduction, methodology, results, and discussion sections, all based on your comments. We hope that these expansions address your concerns and further validate our work. We immensely appreciate giving us the opportunity to revise our manuscript. We would like to thank the editorial team and the associated reviewers for their efforts. We hope that everyone is staying safe and well.

Editorial comments

1. Please ensure that your manuscript meets PLOS ONE's style requirements, including those for file naming.

a. Response: The manuscript style was edited to match PLOS ONE’s style requirements.

2. Please provide additional details regarding participant consent. In the ethics statement in the Methods and online submission information, please ensure that you have specified (1) whether consent was informed and (2) what type you obtained (for instance, written or verbal, and if verbal, how it was documented and witnessed). If your study included minors, state whether you obtained consent from parents or guardians. If the need for consent was waived by the ethics committee, please include this information.

a. Response: The ethical considerations section was updated to accommodate for the abovementioned concerns. The methodology section states that only participants 18 years and older were recruited; therefore, no minors were included in the study.

3. You indicated that you had ethical approval for your study. Please clarify whether minors (participants under the age of 18 years) were included in this study. If yes, in your Methods section, please ensure you have also stated whether you obtained consent from parents or guardians of the minors included in the study or whether the research ethics committee or IRB specifically waived the need for their consent.

a. Response: The ethical considerations section was updated to accommodate for the abovementioned concerns. The methodology section states that only participants 18 years and older were recruited; therefore, no minors were included in the study.

4. Please review your reference list to ensure that it is complete and correct. If you have cited papers that have been retracted, please include the rationale for doing so in the manuscript text, or remove these references and replace them with relevant current references. Any changes to the reference list should be mentioned in the rebuttal letter that accompanies your revised manuscript. If you need to cite a retracted article, indicate the article’s retracted status in the References list and also include a citation and full reference for the retraction notice.

a. Response: The reference list was checked and the any added references resulted from dealing with reviewers’ comments were noted at in the rebuttal letter.

 

Reviewer comments

Abstract

1. Why compare to ophthalmic patients? this demography is expected to demonstrate some knowledge a eye patients. so maybe the public at large would be a better option especially considering the use of self administered questionnaire

a. Response: Dear respected reviewer, the data collection was designed in such a fashion in order to concentrate efforts into the recruitment of patients within one space, that is the ophthalmology clinics. Interestingly enough, glaucoma patients demonstrated far superior knowledge relative to the ophthalmic patients, which could only let us speculate about that of the general public. In fact, an examination of the general public and their glaucoma knowledge is the phase II of this project.

2. Name of the test should be mentioned here. Like regression, correlation etc

a. Response: Type of statistical test was added to the abstract.

Introduction

3. Some explanation here as advanced age would be expected to have more knowledge? especially as it is a disease of older people usually

a. Response: We thank the reviewer for raising such an important point. Explanation to this point was added to the discussion as not to congest the introduction. The following was added: “Interestingly, age was predominantly a negative predictor of glaucoma knowledge within the literature. A variety of factors could’ve influenced such relationship such as the low levels of health literacy in older individuals, neurological disease progression, or the inherent technological bias exhibited by younger participants able to fully utilize the internet to gain disease-related knowledge [34,44].”

4. Some explanation here as well

a. Response: The statement was altered to showcase more clarity. The statement now reads: “Subsequently, those poor clinical presentations are associated with worse treatment outcomes as cases might be treated with more complex surgical options to halt the rapidly progressive and irreversible retinal damage [5,14].”

5. Difference between knowledge and awareness should be made clear

a. Response: We thank the review for their keen comment. The following was added to the introduction: “The literature evaluating glaucoma knowledge within the Middle East and therefore its treatment implications, is scarce. In fact, an accurate estimation of knowledge is tricky to calculate due to the juxtaposition with the concept of awareness. It should be noted that awareness refers to the generalized or diffuse knowledge of a particular concept (e.g., have you heard of glaucoma?), while knowledge refers to any information that is factual in nature (e.g., prevalence, etiology, risk factors, etc.)[16].”

Methodology

6. self-administered questionnaire should be enough, also since this includes patients, an interview with questionnaire could have been a better option

a. Response: We appreciate the reviewer’s keen points. Patients were given the questionnaire and a team of specialized data collectors contacted the patients to ensure that they understood all items provided in the questionnaire. In fact, some older patients with severe visual disability needed the questionnaire to be read for them as they couldn’t read it on their own.

7. Define type of glaucoma, how did you diagnose glaucoma.just mention e.g oct VF etc also exclude other types of glaucoma such as secondary causes

a. Response: The target population in question were those with primary glaucoma. Since closed-angle glaucoma is a medical emergency, it is only logical that all recruited patients had chronic primary open angle glaucoma. Diagnostic modality and an expanded exclusion criteria were added. The new paragraph reads: “Patients were enrolled into two groups: The first group consisted of participants with a clinically proven diagnosis of primary glaucoma of at least 6 months and are on medications. Diagnosis of glaucoma was established on Optical Coherence Tomography. Patients with secondary glaucoma (e.g., pseudoexfoliation syndrome, pigment dispersion syndrome, neovascular glaucoma, inflammatory glaucoma, and medication-induced glaucoma) were excluded. The second group of participants consisted of those who were diagnosed with any vision hindering ophthalmic conditions with the exception of long-standing glaucoma. Such conditions include cataracts, diabetic retinopathy, macular degeneration, optic neuritis among others. Participants that were younger than 18 years of age, suffer from mental/psychiatric illnesses, and are unable to consent to participation in the survey were excluded from the study. Moreover, participants that failed to complete at least 80% of the questionnaire were also excluded.”

8. vision hindering? define term..is it vision threating or just routine patients

a. Response: The term was illuminated on with the use of examples.

9. exclusion and inclusion should be more specific..like glaucoma patients should not have other pathology etc

a. Response: Refer to response to comment number 7

10. Non mutually exclusive? statement 28 through 30 not included in document so cant comment

a. Response: The full questionnaire is now added to the revised submission.

11. any scoring system or just simple addition? was reverse coding utilised as well? as these were self administered questionnaires. without interview, some pariticipants might have filled in a hurry. Reverse coding might have hinted at that

a. Response: Dear respected reviewer, the scoring was a simple addition of all correct items. The knowledge score wasn’t even grouped into poor or good knowledge as such grouping would require validation and diagnostic analysis by ROC curves which outside the scope of this study. As for the reverse coding, as the reviewer knows, participants, especially those with vision ailments, are very sensitive to the number of questions in a questionnaire. Therefore, if reverse-coding was used, a higher rate of participant attrition would occur. However, to combat the issue of participants finishing the questionnaire in a hurry, we implemented the following: 1) providing negative and positive statements throughout the questionnaire, 2) calculating the mean time to finish among participants which was about 8 minutes.

12. can be used for continuous data e.g mean between 2 groups, for categorical use chi square

a. Response: The addition of (for categorical variables with more than two groups) next to the ANOVA test and the t-test was in reference to the INDEPDENT variable. Indeed, the ANOVA and t-test are tests of mean differences by which difference in a dependent continuous variable is tested among 2 independent groups (t-test) or 3+ independent groups (ANOVA). Therefore, to avoid this confusion that we may caused, the brackets were removed.

13. Please confirm this mean is taken from continuuous data, and not categorical data in spss, if it is categorical and we take mean it will be wrong. Just a heads up

a. Response: All data presented as means ± SD were taken from continuous variables. Within the questionnaire number of medications, number of drops per day, and number of surgeries were asked.

14. mention which test is used here not just p value

a. Response: The test used here was the chi squared test. The notion (X2(df) = X, p-value) denotes chi-square and was added accordingly.

15. elaborate on seizures?

a. Response: Simply, we asked participants on what they thought are symptomatic complications of glaucoma. Options were reduced visual acuity, narrow visual field, vision loss, convulsive seizures, and symptoms of inflammation. Convulsive seizures refer to whole body seizures (grand mal seizures).

16. open or closed angle?these symptoms are usually in closed angle glaucoma , which might mean a difference in attitude towards adherence as it is more painful etc

a. Response: Indeed, such symptoms denote a diagnosis of a closed-angle glaucoma. However, participants were asked these questions to test their knowledge of primary glaucoma including both closed and open angle.

17. mention which test is used here. it canot be t test

a. Response: Indeed, each category was compared amongst the ophthalmic and glaucoma patients using the chi-square test. Mentioning of the chi-square test will be added in the footnote of the table.

18. which type of regression is used please mention here

a. Response: Dear respected reviewer, univariate analysis within the context of this paragraph refers to either t-test or ANOVA and not regression (which is a form of multivariate analysis). Continuous variables such as knowledge score, symptom recognition, and adverse effect recognition were examined for difference between 2 groups using the t-test (e.g., biological sex, disease status, or family history of glaucoma), and the between 3 or more groups using the ANOVA test (e.g., residence, education, or income). The notion of (t(df) = X, p-value) denotes the use of the student t-test and the notion of (F(df between groups, df within groups) = X, p-value) denotes the use of the ANOVA per APA guidelines. Both were added accordingly (when applicable).

19. linear regression cannot be used for categorical variables unless a dummy variable is made as a reference....or use logistic regression if the outcome is binary which again required reference variable...here the dummy or reference variable is not shown

a. Response: The reviewer is correct with their knowledge regarding linear regression; however, the linear regression model is found in table 5 and not table 4. Table 4 are mere univariate analysis consisting of t-test and ANOVA as described in the response above.

20. again family history i.e yes or no is categorical, either use dummy variable and show which is the reference variable

a. Response: Dear esteemed reviewer, indeed, family history is a categorical variable. The dummy variable in the model was no family history of glaucoma. The same applies for gender, smoking status (yes/no), and presence of comorbidities. Table 5, which contains the model is updated to contain the reference variables and reduce any confusion to readers.

21. Try to fit univariate and multivariate in 1 table....only show the significant results in the table to make it smaller. This allows easy comparison

a. Response: We appreciate the reviewer’s concern with that regard. However, table 4 (table of univariate analysis) has 3 outcome measures (i.e., knowledge, symptom recognition, and adverse effect recognition scores), while table 5 (table of multivariate analysis) has only knowledge as its outcome variable. Therefore, combining the two tables would create a congested mess of numbers that might be harder to consume by the readers.

Discussion

22. This part might be included introduction to avoid redundancy

a. Response: We thank the reviewer for raising such point. That part of the discussion was reorganized and place within the introduction.

23. how much % of glaucoma patients were aware? since the general population is aware 38.8% , it should be more than this theoretically

a. Response: Dear respected reviewer, indeed, knowledge of glaucoma should be higher among glaucoma patients than the general populace. However, the report which describes glaucoma knowledge among patients visiting Jordan University Hospital did not stratify knowledge per diagnosis. In fact, in their sample of 488, only 20 participants were diagnosed with glaucoma (Refer to: 10.4103/1319-4534.322618)

24. please clear the difference in introduction between awareness and knowledge as it seems to be mixed here/ confusing for the reader. Define it in objectives of study perhaps

a. Response: The following was added to the introduction to illuminate on the differences between awareness and knowledge: “The literature evaluating glaucoma knowledge within the Middle East and therefore its treatment implications, is scarce. In fact, an accurate estimation of knowledge is tricky to calculate due to the juxtaposition with the concept of awareness. It should be noted that awareness refers to the generalized or diffuse knowledge of a particular concept (e.g., have you heard of glaucoma?), while knowledge refers to any information that is factual in nature (e.g., prevalence, etiology, risk factors, etc.)[16].”

25. in my opinion theres is little need for mentioning so many countries.Just local studies and few western studies should be enough perhaps

a. Response: We thank the reviewer for their keen concern. We believe that the inclusion of such studies serves two purposes. Firstly, it shows that variability between country and continents in terms of glaucoma knowledge and/or awareness. Secondly, it serves to provide future researchers and readers with a significant body of literature that they can explore, refer to, and even expand within the context of our results. If the reviewer strongly believes that a reduction should be made, then we are more than happy to comply.

26. just 2 or 3 references should be enough

a. Response: The number of references was reduced to 3 per the reviewer’s advice.

27. Generally the discussion section is redundant in many places, confusing the reader.In my humble opinion it needs to be written coherently , in a stepwise manner and not repeat what has already been established in previous paragraphs. Also there seems to be a few grammatical mistakes. This can be solved by using software such as 'Grammarly'. Of course, Statistical analysis and results section first should be assured free of errors.

a. Response: Dear esteemed reviewer, all points raised with regards to the statistical analysis and results were addressed. Type of statistical testing was shown (resulted with expanded to show such a change) and every part of the analysis was described throughout our responses and changes within the manuscript. As for the discussion, it went through a grammatical check by a native English speaker and we cut at certain parts to avoid redundancy whenever applicable. The new discussion is now presented in the following themes: 1) Summary of findings, 2) Comparison with local literature, 3) Comparison with Western literature, 4) Illuminating on the reasons for variance between different countries/studies, 5) Factors associated with glaucoma knowledge, 6) Recommendations for better glaucoma knowledge and awareness, 7) Limitations.

28. Unclear ? please rephrase if possible

a. Response: The statement aims to stress on the fact that whatever educational intervention that will be given through concerned authorities, it must account that glaucoma have various types and might require different treatments for different risk groups. Therefore, an intervention must be clear, concise, and accommodating. To avoid redundancy, the statement was removed.

Attachment Submitted filename: Rebuttal Letter.docx Click here for additional data file.

# Peer review: Decision Letter 1

**DOI:** 10.1371/journal.pone.0285405.r003

**Authors:** Bharat Gurnani

4 Apr 2023

PONE-D-22-22392R1Predictors of glaucoma knowledge and its risk factors among Jordanian patients with primary open angle glaucoma at a tertiary teaching hospital: a cross-sectional surveyPLOS ONE

Dear Dr. Abdallah Al-Ani

Thank you for submitting your manuscript to PLOS ONE. After careful consideration, we feel that it has merit but does not fully meet PLOS ONE’s publication criteria as it currently stands. Therefore, we invite you to submit a revised version of the manuscript that addresses the points raised during the review process.

Please submit your revised manuscript by May 19 2023 11:59PM. If you will need more time than this to complete your revisions, please reply to this message or contact the journal office at plosone@plos.org. When you're ready to submit your revision, log on to https://www.editorialmanager.com/pone/ and select the 'Submissions Needing Revision' folder to locate your manuscript file.

Please include the following items when submitting your revised manuscript:

- A rebuttal letter that responds to each point raised by the academic editor and reviewer(s). You should upload this letter as a separate file labeled 'Response to Reviewers'.
- A marked-up copy of your manuscript that highlights changes made to the original version. You should upload this as a separate file labeled 'Revised Manuscript with Track Changes'.
- An unmarked version of your revised paper without tracked changes. You should upload this as a separate file labeled 'Manuscript'.

If you would like to make changes to your financial disclosure, please include your updated statement in your cover letter. Guidelines for resubmitting your figure files are available below the reviewer comments at the end of this letter.

If applicable, we recommend that you deposit your laboratory protocols in protocols.io to enhance the reproducibility of your results. Protocols.io assigns your protocol its own identifier (DOI) so that it can be cited independently in the future. For instructions see: https://journals.plos.org/plosone/s/submission-guidelines#loc-laboratory-protocols. Additionally, PLOS ONE offers an option for publishing peer-reviewed Lab Protocol articles, which describe protocols hosted on protocols.io. Read more information on sharing protocols at https://plos.org/protocols?utm_medium=editorial-email&utm_source=authorletters&utm_campaign=protocols.

We look forward to receiving your revised manuscript.

Kind regards,

Bharat Gurnani

Academic Editor

PLOS ONE

Journal Requirements:

Please review your reference list to ensure that it is complete and correct. If you have cited papers that have been retracted, please include the rationale for doing so in the manuscript text, or remove these references and replace them with relevant current references. Any changes to the reference list should be mentioned in the rebuttal letter that accompanies your revised manuscript. If you need to cite a retracted article, indicate the article’s retracted status in the References list and also include a citation and full reference for the retraction notice.

[Note: HTML markup is below. Please do not edit.]

Reviewers' comments:

Reviewer's Responses to Questions

**Comments to the Author**

1. If the authors have adequately addressed your comments raised in a previous round of review and you feel that this manuscript is now acceptable for publication, you may indicate that here to bypass the “Comments to the Author” section, enter your conflict of interest statement in the “Confidential to Editor” section, and submit your "Accept" recommendation.

Reviewer #1: All comments have been addressed

Reviewer #3: (No Response)

Reviewer #4: (No Response)

**********

2. Is the manuscript technically sound, and do the data support the conclusions?

The manuscript must describe a technically sound piece of scientific research with data that supports the conclusions. Experiments must have been conducted rigorously, with appropriate controls, replication, and sample sizes. The conclusions must be drawn appropriately based on the data presented. 

Reviewer #1: Partly

Reviewer #3: Yes

Reviewer #4: Yes

**********

3. Has the statistical analysis been performed appropriately and rigorously? 

Reviewer #1: Yes

Reviewer #3: Yes

Reviewer #4: (No Response)

**********

4. Have the authors made all data underlying the findings in their manuscript fully available?

The PLOS Data policy requires authors to make all data underlying the findings described in their manuscript fully available without restriction, with rare exception (please refer to the Data Availability Statement in the manuscript PDF file). The data should be provided as part of the manuscript or its supporting information, or deposited to a public repository. For example, in addition to summary statistics, the data points behind means, medians and variance measures should be available. If there are restrictions on publicly sharing data—e.g. participant privacy or use of data from a third party—those must be specified.

Reviewer #1: Yes

Reviewer #3: Yes

Reviewer #4: Yes

**********

5. Is the manuscript presented in an intelligible fashion and written in standard English?

PLOS ONE does not copyedit accepted manuscripts, so the language in submitted articles must be clear, correct, and unambiguous. Any typographical or grammatical errors should be corrected at revision, so please note any specific errors here.

Reviewer #1: Yes

Reviewer #3: Yes

Reviewer #4: Yes

**********

6. Review Comments to the Author

Please use the space provided to explain your answers to the questions above. You may also include additional comments for the author, including concerns about dual publication, research ethics, or publication ethics. (Please upload your review as an attachment if it exceeds 20,000 characters)

Reviewer #1: (No Response)

Reviewer #3: The authors describe in this report the predictors of glaucoma knowledge and its risk factors among a group of Jordanian patients attending a tertiary teaching hospital in Jordan. The manuscript is well written and detailed and I congratulate the authors on their extensive work.

Abstract:

1. In the conclusion, when you refer to ophthalmic patients, it is better to describe both glaucoma and non-glaucoma. E.g. We have demonstrated that both glaucoma and non-glaucoma ophthalmic patients display mediocre levels of glaucoma knowledge.

2. Also avoid the use of gross terms such as mediocre.

Introduction:

3. The authors state: “Vision loss associated with glaucoma can be prevented with early diagnosis and treatment; however, due to its asymptomatic nature, lack of awareness of risk, and low levels of referral, the incidence of glaucoma is rising within the population [4].”

The above statement is confusing. It may be better to state that “the incidence/prevalence of glaucoma is under-reported.”

Methods:

4. The word hindering should be substituted with a clearer and simpler term.

5. The authors state: “Such conditions include cataracts, diabetic retinopathy, macular degeneration, and optic neuritis among others.”

Please avoid using “among other”, be specific. Mention the common causes or use causes as they were presented in the results section.

6. In instrument development, the Arabic questionnaire (appendix S2 file) is missing question 11. I am sure this has been attended to, but the authors need to make sure that data have not been compromised by wrong or miss-arrangement order of the questions.

7. Also, 1 point was given to each correct answer for the first 27 questions, and then added together. The authors assume equal weight of each question in the first 27 questions, but this might not be the case. Please clarify why you think all the questions have equal weights?

8. Once again, 1 point is given for the correct answer for questions with a Likert scale, but can the authors explain what is the correct answer if one subject answered” strongly agree” and the another subject answered “ agree”. I assume that categorization into 3 categories eliminated this issue. Please clarify/confirm.

Discussion

9. The authors state: “Our study demonstrated that glaucoma knowledge among Jordanian ophthalmic patients is rather underwhelming”.

Once again, avoid the use of terms such as underwhelming and use only plain scientific terms.

10. The discussion section is rather long and readers can feel bored. It can be improved by shortening the text. Many of the comparisons with other studies can be summarized in a table as an example.

Reviewer #4: (No Response)

**********

7. PLOS authors have the option to publish the peer review history of their article (what does this mean?). If published, this will include your full peer review and any attached files.

If you choose “no”, your identity will remain anonymous but your review may still be made public.

**Do you want your identity to be public for this peer review?** For information about this choice, including consent withdrawal, please see our Privacy Policy.

Reviewer #1: **Yes: **Mahmood Ali

Reviewer #3: No

Reviewer #4: **Yes: **Dr Girum W Gebreal

**********

[NOTE: If reviewer comments were submitted as an attachment file, they will be attached to this email and accessible via the submission site. Please log into your account, locate the manuscript record, and check for the action link "View Attachments". If this link does not appear, there are no attachment files.]

While revising your submission, please upload your figure files to the Preflight Analysis and Conversion Engine (PACE) digital diagnostic tool, https://pacev2.apexcovantage.com/. PACE helps ensure that figures meet PLOS requirements. To use PACE, you must first register as a user. Registration is free. Then, login and navigate to the UPLOAD tab, where you will find detailed instructions on how to use the tool. If you encounter any issues or have any questions when using PACE, please email PLOS at figures@plos.org. Please note that Supporting Information files do not need this step.

Attachment Submitted filename: highlighted review.pdf Click here for additional data file.

Attachment Submitted filename: Predictors of glaucoma knowledge and its risk factors among Jordanian patients with primary open angle glaucoma at a tertiary teaching hospital.docx Click here for additional data file.

# Author comment: Author response to Decision Letter 1

**DOI:** 10.1371/journal.pone.0285405.r004

5 Apr 2023

PLEASE REFER TO THE ATTACHED FILE (COMMENTS ARE PASTED HERE JUST FOR REFERENCE)

Rebuttal Letter

On behalf of myself and all authors within this study, we thank the panel of reviewers for their extensive, shrewd, and informative comments. Any and all changes requested implied through your comments were considered and worked upon in order to strengthen the framework of our manuscript and render it suitable for publication in your respected journal. We would like to thank you for all of your efforts. We edited the manuscript based on your comments and illuminated on areas that were condemned to be vague or misunderstand. We hope that these edits address your concerns and further validate our work. We immensely appreciate giving us the opportunity to revise our manuscript. We would like to thank the editorial team and the associated reviewers for their efforts. We hope that everyone is staying safe and well.

Reviewer 3

Reviewer #3: The authors describe in this report the predictors of glaucoma knowledge and its risk factors among a group of Jordanian patients attending a tertiary teaching hospital in Jordan. The manuscript is well written and detailed and I congratulate the authors on their extensive work.

Abstract:

1. In the conclusion, when you refer to ophthalmic patients, it is better to describe both glaucoma and non-glaucoma. E.g. We have demonstrated that both glaucoma and non-glaucoma ophthalmic patients display mediocre levels of glaucoma knowledge.

RESPONSE: We thank the respected reviewer for their suggestion. The abovementioned edit was conduct accordingly.

2. Also avoid the use of gross terms such as mediocre.

RESPONSE: We thank the reviewer for their suggestions. Words such as “mediocre”, “underwhelming”, and “hindering” were replaced by “average”, “limiting”, and “defective”, respectively.

Introduction:

3. The authors state: “Vision loss associated with glaucoma can be prevented with early diagnosis and treatment; however, due to its asymptomatic nature, lack of awareness of risk, and low levels of referral, the incidence of glaucoma is rising within the population [4].” The above statement is confusing. It may be better to state that “the incidence/prevalence of glaucoma is under-reported.”

RESPONSE: We thank the respected reviewer for their suggestion. The abovementioned edit was conduct accordingly.

Methods:

4. The word hindering should be substituted with a clearer and simpler term.

RESPONSE: We thank the reviewer for their suggestions. Words such as “mediocre”, “underwhelming”, and “hindering” were replaced by “average”, “limiting”, and “defective”, respectively.

5. The authors state: “Such conditions include cataracts, diabetic retinopathy, macular degeneration, and optic neuritis among others.”

Please avoid using “among other”, be specific. Mention the common causes or use causes as they were presented in the results section.

RESPONSE: The statement was completed and altered to the following: “Such conditions include cataracts, diabetic retinopathy, macular degeneration, optic neuritis/atrophy, cerebrovascular disease, and hereditary retinal disorders.”

6. In instrument development, the Arabic questionnaire (appendix S2 file) is missing question 11. I am sure this has been attended to, but the authors need to make sure that data have not been compromised by wrong or miss-arrangement order of the questions.

RESPONSE: We thank the reviewer for their keen observation. The S2 arabic questionnaire file is now fixed. The item was not missing but rather the numbering was shifted and started from 12 instead of 11. The fidelity of the analysis and order of questions within the analysis sheet was rechecked accordingly.

7. Also, 1 point was given to each correct answer for the first 27 questions, and then added together. The authors assume equal weight of each question in the first 27 questions, but this might not be the case. Please clarify why you think all the questions have equal weights?

RESPONSE: We thank the reviewer for pointing out this particular conundrum. According to relevant literature not only limited to glaucoma, the concept of a “reliable self-reported knowledge” instrument might not be achievable in practice. This is simply due to the lack of a “gold standard” for comparison. What constitutes a “knowledgeable” patient/person varies from one physician to another. 

Interestingly, and in line with literature trying to produce glaucoma knowledge scales (e.g., National Eye Health Education Program, Hoevenaars et al., Gray et al.), every single one of them were scored with 1 point given to every correct included statement (DOI: 10.1186/s12886-022-02322-0; 10.4274/tjo.26576; 10.1080/08820538.2016.1247180). This brings us back to the point raised above, why would statement X hold more weight than statement Y but not statement Z. In this case, a true weighting of different statements can only be achieved by expert consensus of a large sample of field experts. 

While our scoring is in line with convenient pattern of the literature of similar glaucoma knowledge questionnaires, we did statistically examine the differences within each statement of the 27 among glaucoma vs. non-glaucoma patients (Refer to table 2).

8. Once again, 1 point is given for the correct answer for questions with a Likert scale, but can the authors explain what is the correct answer if one subject answered” strongly agree” and the another subject answered “ agree”. I assume that categorization into 3 categories eliminated this issue. Please clarify/confirm.

RESPONSE: We thank the reviewer for raising such a point. The 5-point likert scale was condensed as follows: (strongly agree + agree as agree), (neutral = neutral), (strongly disagree + disagree as disagree). We have included that in our statistical analysis part of the methodology.

Discussion

9. The authors state: “Our study demonstrated that glaucoma knowledge among Jordanian ophthalmic patients is rather underwhelming”.

Once again, avoid the use of terms such as underwhelming and use only plain scientific terms.

RESPONSE: We thank the reviewer for their suggestions. Words such as “mediocre”, “underwhelming”, and “hindering” were replaced by “average”, “limiting”, and “defective”, respectively.

10. The discussion section is rather long and readers can feel bored. It can be improved by shortening the text. Many of the comparisons with other studies can be summarized in a table as an example.

RESPONSE: We thank the reviewer for their comment. Indeed, the discussion is pretty verbose. While having a table to discuss differences in study findings may be more suitable for a systematic review, we have reduced the discussion from 1750 words to 1289 words. We have removed any and all redundancies. Moreover, any information that can be extracted from the original references were removed as to avoid repeating large sums of information.

 

Reviewer 4

ABSTRACT

The purpose of the study is mentioned to be to assess the glaucoma knowledge and adherence to care seeking behaviors. However, there is no mention of adherence related data included in the study. 

RESPONSE: We thank the reviewer for raising this point, the aim/objective part of the abstract now reads: “To assess and compare glaucoma knowledge between Jordanian patients with glaucoma and non-glaucoma ophthalmic patients.”.

INTRODUCTION

Paragraph 1- ‘due to its asymptomatic nature, lack of awareness of risk, and low levels of referral, the incidence of glaucoma is rising within the population’. Incidence refers to number of new cases in a certain period. The factors you mentioned may contribute to a rise of glaucoma blindness but not incidence of glaucoma.

RESPONSE: We thank the reviewer for their suggestion. Upon reviewing the original reference, those factors were associated with the increase in prevalence and not incidence. The statement was altered accordingly.

MATERIALS AND METHODS

What is the explanation to use these two groups as study method?

RESPONSE: The study adopts a cross-sectional design in which two groups (one with glaucoma vs. another without glaucoma) were examined for glaucoma-related differences. The data collection was designed in such a fashion in order to concentrate efforts into the recruitment of patients within one space, that is the ophthalmology clinics. Interestingly enough, glaucoma patients demonstrated far superior knowledge relative to the non-glaucoma ophthalmic patients, which could only let us speculate about that of the general public. In fact, an examination of the general public and their glaucoma knowledge is the phase II of this project.

Group 1 consisted of POAG and such patients are expected to be 40 years of age or above. However, the age limit included in the study is above 18 years of age. It appears those with Juvenile Open Angle Glaucoma may have been included. It is better to be clear about this. 

RESPONSE: We thank the reviewer for their expert comment. However, no cases of Juvenile Open Angle Glaucoma were included. The 18 years age limit is concerned with the ethical considerations of the study and must be routinely included. If we are to include patients from under 18 years of age, a different consent form and ethical considerations will be applied. Therefore, the 18 years of age limit is only arbitrary and serves as a complementary statement to the ethical consideration section.

How could OCT alone establish the diagnosis of glaucoma?

RESPONSE: We appreciate the esteemed reviewer’s concern. Recruited glaucoma were not diagnosis based on OCT alone but rather the usual mix of optic nerve examination, clinical symptoms, perimetry, gonioscopy, and OCT/RNFL visualization. For the sake of ease, we included only the objective imaging modality. However, for the sake of completion, we have updated the statement to the following: “Diagnosis of glaucoma was established using slit lamp examination, visual field testing, gonioscopy, and imaging (i.e., Optical Coherence Tomography)”.

It is mentioned simple random sampling is utilized and every 4th patient was recruited. There is inadequate information to explain why this method was used. For example, What was the sample size? Did you have a sampling frame?

RESPONSE: Dear reviewer, random sample is the best method to yield samples that do not have skewed characteristics. While recruiting such a sample may not be always the case due to methodological errors in data collection, random sampling is superior to convenient (a.k.a non-random) sampling. Moreover, when calculating sample sizes, the resultant minimum recommended sample size is expected to be recruited through random sampling.

With regards to our calculated sample size, we did not calculate a population-based sample size. There is no reliable data about the number of glaucoma patients in Jordan, therefore, we are sampling from an unknown population. However, we have conducted a test-oriented sample size calculation. The reviewer should be rest assured that to conduct power chi-square, t-test/ANOVA, and regression, a minimum of 80, 220 (110 per group), and 172 participants are required (G*Power, alpha error 5%, power 95%). Our sample satisfies the aforementioned requirements.

RESULTS

There is no operational definition given to identify the level of knowledge of participants. You mentioned in your discussion that your study demonstrated glaucoma patients are more knowledgeable about glaucoma than their non-glaucoma counterparts. Where is the cutoff score to say one is knowledgeable? 

RESPONSE: We thank the reviewer for pointing out this particular issue. According to relevant literature not only limited to glaucoma, the concept of a “reliable self-reported knowledge” instrument might not be achievable in practice. This is simply due to the lack of a “gold standard” for comparison. What constitutes a “knowledgeable” patient/person varies from one physician to another. 

In fact, the literature involving glaucoma knowledge questionnaire doesn’t have any cut-off points (DOI: 10.1186/s12886-022-02322-0; 10.4274/tjo.26576; 10.1080/08820538.2016.1247180; among others). Even when cut-off points are made, they are often arbitrary either based on quartiles or the median value for the entire score. 

Not to deviate, our statement refers to the fact that glaucoma patients have “relatively” more knowledge than their non-glaucoma counterparts; a finding proven through statistical analysis (i.e., higher knowledge scores for the earlier group). The statement was altered to include “relatively” to avoid misconceptions.

There are some factors that were found to be positive predictors of higher glaucoma knowledge scores. However, it is not clear whether those factors were analyzed for the entire sample (two groups together) or for the glaucoma patients (group 1) alone. Those predictor factors may not be associated for the two groups if analyzed separately.

RESPONSE: We thank the esteemed reviewer for raising such an important point. The analysis was done for both groups combined. Unfortunately, this is a product of limitation as regression analysis requires large sample sizes to be statistically relevant. Our G*Power sample size calculation requires at least 170 participants for a linear regression to be relevant. This is only applicable to the sample as a whole and not its glaucoma non-glaucoma subgroups. Therefore, we only edited the table’s title to “Predictors of knowledge score for the studied sample (n = 256)” to avoid confusion and increase clarity. This limitation was accordingly added to the limitations section. 

DISCUSSION

You included many literature references from Asia to Africa that mentioned the level of awareness of glaucoma but you have not mentioned the level of awareness you found amongst your participants. 

RESPONSE: We understand the esteemed reviewer’s concern. Within our definitions, we state “It should be noted that awareness refers to the generalized or diffuse knowledge of a particular concept (e.g., have you heard of glaucoma?), while knowledge refers to any information that is factual in nature (e.g., prevalence, etiology, risk factors, etc.)[16].”. Knowledge was the primary aim of this questionnaire and study and not awareness. We did not have a “have you heard of glaucoma” or “have you heard of glaucoma’s treatments” kind of questions (which measure superficial knowledge = awareness) but rather had statements that measure deep knowledge which is referred to as knowledge.

However, our inclusion of studies that only measure awareness, such as those originating from Asia to Africa, is to provide a comprehensive view of all the literature that attempted to measure awareness or knowledge of glaucoma. What’s even more peculiar is that the use of awareness and knowledge is often interchangeable within the literature; an issue well documented across many topics and disciplines that aim to measure knowledge (DOI: 10.3389/fpubh.2017.00194).

 

Reviewer 1

(Comments highlighted on PDF)

1. Grammatical issues were fixed per the reviewer’s comments

2. The tables were altered per the reviewer’s comments

3. In response to “Were all these variables significant in univariate analysis? Only then should be added in multivariate model”. 

RESPONSE: We fully understand the reviewer’s concern. When creating models of prediction, such as that of regression, the inclusion of variables can be made through a variety of methods. These methods include 1) significant variables on univariate analysis (which was pointed out by the respected reviewer, 2) variables with p-values less than 0.10 in the univariate analysis, 3) “a priori” inclusion of such variables during the pre-research conduction stage, or 4) variables that demonstrated association with the dependent outcomes (i.e., knowledge) throughout the literature. A mix of the aforementioned methods is feasible as long as it produces models that are free of collinearity and have the highest explanatory power. Our presented model had no collinearity based on the Durbin-Watson statistic and had the highest R2 value out of all the other attempted models.

4. The limitations section was edited per the reviewer’s comments

Attachment Submitted filename: Rebuttal Letter.docx Click here for additional data file.

# Editor report: Decision Letter 2

**DOI:** 10.1371/journal.pone.0285405.r005

**Authors:** Bharat Gurnani

24 Apr 2023

Predictors of glaucoma knowledge and its risk factors among Jordanian patients with primary open angle glaucoma at a tertiary teaching hospital: a cross-sectional survey

PONE-D-22-22392R2

Dear Dr. %Abdallah Al-Ani%,

We’re pleased to inform you that your manuscript has been judged scientifically suitable for publication and will be formally accepted for publication once it meets all outstanding technical requirements.

Within one week, you’ll receive an e-mail detailing the required amendments. When these have been addressed, you’ll receive a formal acceptance letter and your manuscript will be scheduled for publication.

An invoice for payment will follow shortly after the formal acceptance. To ensure an efficient process, please log into Editorial Manager at http://www.editorialmanager.com/pone/, click the 'Update My Information' link at the top of the page, and double check that your user information is up-to-date. If you have any billing related questions, please contact our Author Billing department directly at authorbilling@plos.org.

If your institution or institutions have a press office, please notify them about your upcoming paper to help maximize its impact. If they’ll be preparing press materials, please inform our press team as soon as possible -- no later than 48 hours after receiving the formal acceptance. Your manuscript will remain under strict press embargo until 2 pm Eastern Time on the date of publication. For more information, please contact onepress@plos.org.

Kind regards,

Bharat Gurnani

Academic Editor

PLOS ONE

Additional Editor Comments (optional):

Reviewers' comments:

# Editor report: Acceptance letter

**DOI:** 10.1371/journal.pone.0285405.r006

**Authors:** Bharat Gurnani

10 May 2023

PONE-D-22-22392R2 

Predictors of glaucoma knowledge and its risk factors among Jordanian patients with primary open angle glaucoma at a tertiary teaching hospital: a cross-sectional survey 

Dear Dr. Al-Ani:

I'm pleased to inform you that your manuscript has been deemed suitable for publication in PLOS ONE. Congratulations! Your manuscript is now with our production department. 

If your institution or institutions have a press office, please let them know about your upcoming paper now to help maximize its impact. If they'll be preparing press materials, please inform our press team within the next 48 hours. Your manuscript will remain under strict press embargo until 2 pm Eastern Time on the date of publication. For more information please contact onepress@plos.org.

If we can help with anything else, please email us at plosone@plos.org. 

Thank you for submitting your work to PLOS ONE and supporting open access. 

Kind regards, 

PLOS ONE Editorial Office Staff

on behalf of

Dr. Bharat Gurnani 

Academic Editor

PLOS ONE
